# Fibroblast growth factor signals regulate transforming growth factor‐β‐induced endothelial‐to‐myofibroblast transition of tumor endothelial cells via Elk1

**DOI:** 10.1002/1878-0261.12504

**Published:** 2019-06-19

**Authors:** Yuichi Akatsu, Naoya Takahashi, Yasuhiro Yoshimatsu, Shiori Kimuro, Tomoki Muramatsu, Akihiro Katsura, Nako Maishi, Hiroshi I. Suzuki, Johji Inazawa, Kyoko Hida, Kohei Miyazono, Tetsuro Watabe

**Affiliations:** ^1^ Department of Molecular Pathology Graduate School of Medicine The University of Tokyo Japan; ^2^ Biomedicine Group Pharmaceutical Research Laboratories Pharmaceutical Group Nippon Kayaku Co., Ltd. Tokyo Japan; ^3^ Department of Biochemistry Graduate School of Medical and Dental Sciences Tokyo Medical and Dental University (TMDU) Japan; ^4^ Department of Molecular Cytogenetics Medical Research Institute Tokyo Medical and Dental University (TMDU) Japan; ^5^ Department of Vascular Biology and Molecular Pathology Graduate School of Dental Medicine Hokkaido University Sapporo Japan; ^6^ David H. Koch Institute for Integrative Cancer Research Massachusetts Institute of Technology Cambridge MA USA

**Keywords:** Elk1, EndMT, End‐MyoT, End‐N‐MyoT, FGF2, TGF‐β2

## Abstract

The tumor microenvironment contains various components, including cancer cells, tumor vessels, and cancer‐associated fibroblasts, the latter of which are comprised of tumor‐promoting myofibroblasts and tumor‐suppressing fibroblasts. Multiple lines of evidence indicate that transforming growth factor‐β (TGF‐β) induces the formation of myofibroblasts and other types of mesenchymal (non‐myofibroblastic) cells from endothelial cells. Recent reports show that fibroblast growth factor 2 (FGF2) modulates TGF‐β‐induced mesenchymal transition of endothelial cells, but the molecular mechanisms behind the signals that control transcriptional networks during the formation of different groups of fibroblasts remain largely unclear. Here, we studied the roles of FGF2 during the regulation of TGF‐β‐induced mesenchymal transition of tumor endothelial cells (TECs). We demonstrated that auto/paracrine FGF signals in TECs inhibit TGF‐β‐induced endothelial‐to‐myofibroblast transition (End‐MyoT), leading to suppressed formation of contractile myofibroblast cells, but on the other hand can also collaborate with TGF‐β in promoting the formation of active fibroblastic cells which have migratory and proliferative properties. FGF2 modulated TGF‐β‐induced formation of myofibroblastic and non‐myofibroblastic cells from TECs via transcriptional regulation of various mesenchymal markers and growth factors. Furthermore, we observed that TECs treated with TGF‐β were more competent in promoting *in vivo* tumor growth than TECs treated with TGF‐β and FGF2. Mechanistically, we showed that Elk1 mediated FGF2‐induced inhibition of End‐MyoT via inhibition of TGF‐β‐induced transcriptional activation of α‐smooth muscle actin promoter by myocardin‐related transcription factor‐A. Our data suggest that TGF‐β and FGF2 oppose and cooperate with each other during the formation of myofibroblastic and non‐myofibroblastic cells from TECs, which in turn determines the characteristics of mesenchymal cells in the tumor microenvironment.

**Abbreviations**
CAF: cancer‐associated fibroblast
EMT: epithelial‐to‐mesenchymal transition
EMyoT: epithelial‐to‐myofibroblast transition
EndMT: endothelial‐to‐mesenchymal transition
End‐MyoT: endothelial‐to‐myofibroblast transition
End‐N‐MyoT: endothelial‐to‐non‐myofibroblast transition
ERK: extracellular signal‐regulated kinase
FGF: fibroblast growth factor
HB‐EGF: heparin‐binding EGF‐like growth factor
MEK: mitogen‐activated protein kinase kinase
MRTF: myocardin‐related transcription factor
NEC: normal endothelial cells
Nox4: NADPH oxidase 4
PDGF: platelet‐derived growth factor
qRT‐PCR: quantitative reverse transcription‐PCR
Rgs4: regulator of G protein signaling 4
SM22α: smooth muscle 22α
SMC: smooth muscle cell
SRF: serum response factor
TEC: tumor endothelial cell
TGF‐β: transforming growth factor‐β
α‐SMA: α‐smooth muscle actin

## 1 Introduction

The tumor microenvironment is composed of tumor cells as well as other components, including tumor vessels and cancer‐associated fibroblasts (CAFs). Multiple lines of evidence suggest that CAFs can be defined as a heterogenous cell population based on the expression profile of several mesenchymal markers including α‐smooth muscle actin (α‐SMA), a prototypical marker for myofibroblasts (Augsten, 2014; Madar *et al*., 2013). CAFs are subgrouped into tumor‐suppressing (type I) and tumor‐promoting (type II) populations. Type I CAFs are characterized by the lack of the expression of α‐SMA and have been shown to suppress tumor growth (Augsten, 2014). In contrast, expression of an array of mesenchymal markers including α‐SMA, fibroblast specific protein 1, and vimentin is upregulated in type II CAFs. Type II CAFs promote tumor progression via interaction with cancer cells and other stromal cells present within the tumor microenvironment to orchestrate tumor angiogenesis and metastasis (Augsten, 2014).

Heterogeneity of CAFs is determined by various factors including the tissue type in which the cancer grows, the local paracrine cues, and the cell type of origin of CAFs. CAFs can be originated from local fibroblasts and tissue‐resident fibroblast precursor cells that become incorporated into the growing tumor. Recent reports have shown that a part of CAFs arise from tumor vessels via endothelial‐to‐mesenchymal transition (EndMT; Fig. S1). During this process, endothelial cells lose their characteristics such as cell–cell contact and expression of endothelial cell‐specific markers, including vascular endothelial cadherin, and acquire mesenchymal phenotypes leading to the expression of various mesenchymal markers (Solit *et al*., 2008; Yoshimatsu and Watabe, 2011).

Recent lines of evidence suggest that mesenchymal cells originated from endothelial cells are heterogeneous in terms of the expression of specific markers as well as biological properties (Fig. S1: Xiao and Dudley, 2017). While some mesenchymal cells originated from endothelial cells express α‐SMA and possess characteristics of myofibroblasts, others derived from endothelial cells do not show such features. These two groups of the cells are likely formed via two different types of mesenchymal transitions. Here, we define the mesenchymal transition which gives rise to myofibroblasts as an endothelial‐to‐myofibroblast transition (End‐MyoT) and those giving rise to non‐myofibroblastic mesenchymal cells as an endothelial‐to‐non‐myofibroblast transition (End‐N‐MyoT; Fig. S1). EndMT, that is subgrouped into End‐MyoT and End‐N‐MyoT, does not only result in the formation of CAFs but also weakens the endothelial barrier of the tumor vasculature facilitating metastatic dissemination. Thus, EndMT is now considered as potential novel targets for anticancer therapies (Gasparics *et al*., 2016). Many tumor types are characterized by a high concentration of transforming growth factor‐βs (TGF‐βs), which are produced by cancer cells and infiltrating inflammatory cells. Previous studies have shown that TGF‐β promotes epithelial‐to‐mesenchymal transition (EMT) of normal and transformed epithelial cells, as well as EndMT of endothelial cells (Miyazono *et al*., 2018; Yoshimatsu and Watabe, 2011).

We have previously reported that TGF‐β promotes different types of EMT resulting in the formation of both myogenic (myofibroblast) and non‐myogenic mesenchymal cells (Shirakihara *et al*., 2011). Long‐term exposure of epithelial cells to TGF‐β induces epithelial‐to‐myofibroblastic transition (EMyoT) by inactivating the MAPK/ERK kinase (MEK)–ERK pathway. Addition of fibroblast growth factor 2 (FGF2) perturbs EMyoT by reactivating the MEK–ERK pathway and subsequently enhances non‐myogenic EMT, which gives rise to more migratory and invasive mesenchymal‐like cell type. Recent reports have shown that signals mediated by FGF2 also inhibit TGF‐β‐induced EndMT (Chen *et al*., 2012, 2014, 2015; Correia *et al*., 2016; Xiao *et al*., 2015).

Recent reports suggest that FGF2 stimulates several distinct pathways to modulate EndMT. FGF2 induces the expression of miRNA such as let‐7 and miR‐20a, which target various components of TGF‐β signaling cascades (Chen *et al*., 2012, 2014, 2015; Correia *et al*., 2016). In addition, activation of MEK/ERK by FGF2 in lymphatic endothelial cells downregulates TGF‐β‐induced phosphorylation of SMAD2, a downstream component of TGF‐β signals, which finally leads to the suppression of EndMT (Ichise *et al*., 2014). However, it has not been clear whether FGF2 inhibits End‐MyoT or End‐N‐MyoT. Furthermore, the molecular mechanisms underlying the action of FGF2 have not yet been fully elucidated.

We have previously reported that TGF‐β2 induces EndMT of various types of endothelial cells (Katsura *et al*., 2016; Kokudo *et al*., 2008; Mihira *et al*., 2012; Suzuki *et al*., 2017). We have also shown that TGF‐β2 stimulates EndMT via regulation of various transcription factors, including Snail and myocardin‐related transcription factor‐A (MRTF‐A). MRTF‐A is a member of the myocardin family of transcription factors (Hinson *et al*., 2007; Morita *et al*., 2007). During the activation of smooth muscle cells (SMCs), myocardin regulates the expression of SMC‐related genes, including α‐SMA by binding to serum response factor (SRF), followed by association with the serum response element DNA consensus site. This interaction is competed with activated Elk1, a ternary complex factor, that belongs to a subclass of the ETS family of transcription factors (Besnard *et al*., 2011), and acts as a competitor of the myocardin family transcription factors (Wang *et al*., 2004). The activation of Elk1 in SMCs is induced by MEK signals upon the stimulation with platelet‐derived growth factor (PDGF). While myocardin is not expressed by endothelial cells, we have previously found that TGF‐β2 increases the expression of MRTF‐A in endothelial cells and that this MRTF‐A expression is required for TGF‐β2‐induced EndMT (Mihira *et al*., 2012). Furthermore, our other study showed Elk1 to be a target of microRNA‐27, which is involved in TGF‐β2‐induced EndMT (Suzuki *et al*., 2017), suggesting Elk1 to participate in EndMT.

Since TGF‐β enriched in tumor environment induces mesenchymal transition of endothelial cells, tumor endothelial cells (TECs) need to protect themselves from TGF‐β via distinct mechanisms. We have reported that TECs differ from normal endothelial cells (NECs) (Hida *et al*., 2013). TECs proliferate more rapidly and are more resistant to starvation or chemotherapeutic drugs than NECs, partially due to a higher level of secretion of vascular endothelial growth factor (VEGF) (Kurosu *et al*., 2011). In addition, Xiao and colleagues reported that TGF‐β‐induced EndMT was modulated by FGF2 secreted from TECs isolated from tumor tissues formed in the transgenic mouse models (Xiao *et al*., 2015). However, the molecular mechanisms underlying FGF signal‐dependent regulation of transcriptional networks during the modulation of End‐MyoT and End‐N‐MyoT of TECs largely remain unclear. In the present study, we attempted to elucidate the roles of FGF2 and Elk1 in the regulation of TGF‐β‐induced End‐MyoT and End‐N‐MyoT of TECs derived from xenograft model of human cancer cells.

## 2 Materials and methods

### 2.1 Cell culture and reagents

MS‐1, mouse pancreatic microvascular endothelial cells, and NIH/3T3 mouse embryo fibroblast cells were obtained from American Type Culture Collection (Manassas, VA, USA). MC3T3‐E1 osteoblast precursor cells were purchased from the RIKEN Bioresource Center (RCB1126; Tsukuba, Japan). MS‐1 and MC3T3‐E1 were maintained in minimum essential medium‐α (Thermo Fisher Scientific, Waltham, MA, USA) supplemented with 10% FBS, 50 U·mL^−1^ penicillin, and 50 μg·mL^−1^ streptomycin. NIH/3T3 cells were maintained in Dulbecco's modified Eagle's medium (DMEM): high glucose (4.5 g·L^−1^; Nacalai Tesque, Kyoto, Japan). Mouse TECs were isolated from human A375SM melanoma tumor xenografted in nude mice as previously described (Hida *et al*., 2004) and grown in basal EGM‐2MV medium (CC‐3202; Lonza, Basel, Switzerland) containing 5% FBS. The A375 cells were maintained in DMEM: high glucose (08458‐16; Nacalai Tesque) supplemented with 10% FBS (Sigma‐Aldrich, St. Louis, MO, USA). Human TGF‐β2 (302‐B2‐010; R&D systems, Minneapolis, MN, USA: 1 ng·mL^−1^), human FGF2 (233‐FB‐010; R&D systems: 50 ng·mL^−1^ or Peprotech; Rocky Hill, NJ, USA), Infigratinib (I‐1500; LC Laboratories, Woburn, MA, USA: 3 μm), VEGF‐A (30 ng·mL^−1^; R&D), and SB431542 (10 μm; Wako, Saitama, Japan) were used in each experiment. Anti‐FGF2 neutralizing antibody and control IgG1 (immunoglobulin G1) were purchased from Merck Millipore (05‐117, Billerica, MA, USA) and Cell Signaling Technology (5415, Danvers, MA, USA), respectively. Recombinant mouse FGF2 (450‐33) was obtained from Peprotech.

### 2.2 RNA interference

siRNA were introduced into cells as described previously (Yoshimatsu *et al*., 2013). Stealth siRNA against mouse Elk1 (siElk1#1: MSS203835, 5′‐TCACGGGATGGTGGTGAGTTCAAGT‐3′ and siElk1#2: MSS203834, 5′‐GGGCCTCTATTCTACCTTCACAATA‐3′) were predesigned at BLOCK‐iT RNAi Express (Thermo Fisher Scientific). Control Stealth siRNA was purchased from Thermo Fisher Scientific (12935‐300, sequence not available). The siRNA were introduced into TECs using Lipofectamine™ RNAiMAX Transfection Reagent (13778‐150; Thermo Fisher Scientific) according to the protocol suggested by the manufacturer.

### 2.3 Lentiviral production and infection

A lentiviral vector encoding Elk1 was generated by Gateway Technology (Thermo Fisher Scientific) as described previously (Suzuki *et al*., 2010). Briefly, Elk1 cDNA (a gift from H. Sugimoto, Dokkyo Medical University) was subcloned into the pENTR vector (Thermo Fisher Scientific) and subsequently transferred into the pCSII‐EF‐RfA lentiviral expression vector (a gift from H. Miyoshi, Keio University) by the LR recombination reaction (Thermo Fisher Scientific). GFP‐bearing CS‐CDF‐CG‐PRE plasmid was used as a control lentiviral vector. The 293FT cells were co‐transfected with the expression plasmids and packaging plasmids (pCMV‐VSV‐G‐RSV‐Rev and pCAG‐HIVgp) using Lipofectamine 2000 (11668019; Thermo Fisher Scientific). The viral supernatants were collected 48 h after transfection. For viral infection, 5.0 × 10^4^ TEC cells per well in 12‐well tissue culture plates were infected with lentiviral particles.

### 2.4 Immunoblot analysis

Immunoblot analysis was performed as described previously (Goto *et al*., 2007). Antibodies to α‐SMA (D4K9N; 19245; Cell Signaling Technology), smooth muscle 22α (SM22α; ab14106; Abcam, Cambridge, UK), β‐actin (A1978; SIGMA), FGF2 (5039‐100; Biovision, Milpitas, CA, USA), phospho‐ERK1/2 (137F5; 4695; Cell Signaling Technology), and ERK‐1/2 (610124; BD Biosciences, San Jose, CA, USA) were used.

### 2.5 Immunocytochemistry

Immunocytochemistry of culture cells was performed as described previously (Shirakihara *et al*, 2007), using monoclonal antibodies to Tie2 (bs‐1300R; Bioss Antibodies, Woburn, MA, USA) and SMA‐Cy3 (clone 1A4; SIGMA). Samples were incubated with secondary antibodies: Alexa Fluor 488 Donkey Anti‐Rabbit IgG (H+L; A‐21206; Thermo Fisher Scientific). Stained cells were photographed under a fluorescence microscope (BZ‐X710; KEYENCE, Osaka, Japan) with 20× objective lenses. Nuclei were counterstained with Hoechst 33342 (4082; Cell Signaling Technology).

### 2.6 RNA isolation and quantitative RT‐PCR

Total RNA was prepared with RNeasy reagent (74160; QIAGEN, Hilden, Germany) or Nucleospin RNA (740902; TaKaRa Bio, Otsu, Japan) and was reverse transcribed by random priming using a PrimeScript II 1st strand cDNA Synthesis Kit (6210B; TaKaRa Bio). Quantitative reverse transcription‐PCR (qRT‐PCR) analysis was performed using StepOne Plus Real‐Time PCR System (Applied Biosystems, Foster City, CA, USA). All expression data were normalized to the expression of glyceraldehyde‐3‐phosphate dehydrogenase. The primer sequences are available in Table S1.

### 2.7 cDNA microarray

A cDNA microarray study was done using cDNA isolated from TECs treated with TGF‐β2, FGF2, or combination of TGF‐β2 and FGF2 for 72 h (Fig. 5); from TECs treated with TGF‐β2, FGF2, or combination of TGF‐β2 and FGF2 for 72 h (Figs S6 and S10); and from TECs treated with TGF‐β2, combination of TGF‐β2 and FGF2, or combination of TGF‐β2 and Infigratinib for 72 h (Fig. S7). The array was performed by TaKaRa Bio, using the Agilent Expression Array service (SurePrint G3 Mouse GE 8 × 60K Ver2.0; Agilent, Santa Clara, CA, USA). Raw data were normalized and log‐transformed with genespring gx 14.9 (Agilent). Transcripts upregulated by TGF‐β2 were selected using fold change cutoffs of ≧ 3.5. Furthermore, the transcripts altered by FGF2 were determined based on fold changes in the range of ≧ 6 and ≦ −6 (Fig. 5). Transcripts downregulated by TGF‐β2 were also selected using fold change cutoffs of ≧ −3.5. Then, the selected transcripts altered by combinatory treatment of TGF‐β2 and FGF2 were determined based on fold changes in the range of ≧ 6 and ≦ −6 (Fig. S6). Transcripts upregulated by TGF‐β2 were selected using fold change cutoffs of ≧ 1.5. Then, the selected transcripts altered by combinatory treatment of TGF‐β2 and FGF2, or TGF‐β2 and Infigratinib were determined based on fold changes by comparing TGF‐β2‐treated samples with both TGF‐β2‐ and FGF2‐treated ones in the range of ≧ 6 and ≦ −6 (Fig. S7). Transcripts upregulated or downregulated by FGF2 were also selected using fold change cutoffs of ≧ 8.7 or ≦ −8.7, respectively. Then, the selected transcripts altered by TGF‐β2 were determined based on fold changes in the range of ≧ 10 and ≦ −10 (Fig. S10).

### 2.8 Chamber migration assay

Modified Boyden chamber migration assay was used for the measurements of migratory ability of TECs. Briefly, TECs were cultured in the absence or presence of TGF‐β2 in combination with FGF2 for 72 h and seeded (6.0 × 10^4^ cells/0.2 mL EGM‐2MV medium) into the upper chamber of a 24‐well transwell with 8 μm pore filter (353097; Corning, New York, NY, USA). Cells were allowed to migrate for 6 h. Nonmigrated cells were removed from the upper chamber by using a cotton swab, and the filter from each treatment was individually stained with Diff‐Quick (16920; SYSMEX, Kobe, Japan). Migrated cells adhering to the bottom side of the chamber were photographed and counted using a light microscope at 10× magnification.

### 2.9 Tube formation assay

A 12‐well plate was precoated with matrigel (BD Biosciences; 0.4 mL·well^−1^). After polymerization of matrigel at 37 °C for 1 h, TECs were seeded into each well with a density of 2 × 10^5^ cells/500 μL medium per well. After 8 h of incubation in EGM‐2MV medium supplemented with 5% FBS, tube‐like structures were photographed under phase‐contrast microscopy (BZ‐X710; KEYENCE) with 10× objective lenses. Tube length was quantified using imagej (US National Institutes of Health, Bethesda, MD, USA).

### 2.10 Collagen gel contraction assay

Collagen gel contraction assay was performed as described previously (Mihira *et al*., 2012). Briefly, 1 mL of the mixture containing type I collagen (180425, Cellmatrix I‐P; Nitta Gelatin, Osaka, Japan) and TECs (1.0 × 10^6^ cells) was added to each well of 12‐well culture plates, allowed to solidify at 37 °C for 2 h, and overlaid with 1 mL of EGM‐2MV medium to float the gel. The floating gels were incubated at 37 °C in 5% CO_2_ for 2 days. The relative changes in a gel surface area were quantified using imagej software (US National Institutes of Health).

### 2.11 Phalloidin staining

F‐actin was stained by fixing the TECs cultured with or without TGF‐β2 and/or FGF2 for 72 h in 4% paraformaldehyde and treating with 0.1% Triton X‐100, followed by the incubation with Alexa Fluor 647‐Phalloidin (A22287; Thermo Fisher Scientific). Stained cells were photographed using a fluorescence microscope (BZ‐X710; KEYENCE) with 40× objective lenses. Nuclei were counterstained with Hoechst 33342 (4082; Cell Signaling Technology).

### 2.12 Cell proliferation assay

Tumor endothelial cells were seeded into six‐well plate and grown for an indicated period, followed by direct cell counting with hemocytometer. The experiments were performed in triplicate.

### 2.13 Luciferase assay

Luciferase assay was performed as described elsewhere (Mihira *et al*., 2012). Briefly, MS‐1 cells were seeded into 24‐well plates and transiently transfected with luciferase reporter plasmid carrying the promoter sequence of the α‐SMA gene (−724/+51 sequence) with or without expression plasmids encoding MRTF‐A and Elk1 using ViaFect Transfection Reagent (E4981; Promega). Cells were cultured in the absence or presence of TGF‐β2 or FGF2 for 20 h. The cell lysates were then prepared, followed by a measurement of luciferase activities in the lysates with the Dual‐Luciferase Reporter System (E‐1910; Promega, Fitchburg, WI, USA) using a luminometer (Lumat^3^ LB9508; Berthold Technologies, Bad Wildbad, Germany). Values were normalized to the Renilla luciferase activity driven by thymidine kinase promoter.

### 2.14 Subcutaneous xenograft model

Animal experiments were approved by Tokyo Medical and Dental University (registration number: A2018‐210C) and were done according to the guidelines of the Animal Care Standards. The A375 cells and TECs that were treated with TGF‐β2 or combination of TGF‐β2 and FGF2 were inoculated subcutaneously (s.c.) into the left flank of BALB/c 7 or 8‐week‐old female immunodeficient nude mice by injection of 1 × 10^6^ cells of A375 cells and 3 × 10^5^ cells of TECs in 200 μL of PBS‐diluted Matrigel (354234; Corning), respectively. Tumor formation was examined at the indicated time points. Tumor volume (*V*) was calculated using the formula: *V* (mm^3^) = *L* × *W* ^2^ × 0.5, in which *L* corresponds to the length of the tumor in mm and *W* to the width of the tumor in mm, respectively.

### 2.15 Immunohistochemistry

Immunofluorescent staining of human melanoma xenografts was done as described previously (Suzuki *et al*., 2010). Briefly, blood vessels in xenografted tumors were evaluated after the tumors were harvested on day 24 by immunostaining using anti‐Ki67 antibody (ab15580; Abcam) and anti‐platelet and endothelial cell adhesion molecule (PECAM‐1) antibody (55370; BD Biosciences). Samples were incubated with secondary antibodies: Alexa Fluor 488 Donkey anti‐Rat IgG (H+L; A‐21208; Thermo Fisher Scientific) and Alexa Fluor 594 Donkey anti‐Rabbit IgG (H+L; R37119; Thermo Fisher Scientific).

### 2.16 Statistical analyses

Values are presented as mean ± standard deviation or standard error. Significant differences between means were determined using one‐tailed unpaired Student's *t*‐test or one‐way ANOVA followed by the Student–Newman–Keuls test. Differences between means were considered statistically significant at *P < 0.05.

### 2.17 Data deposition

The microarray data produced in our study have been submitted to the GEO database (http://www.ncbi.nlm.nih.gov/geo/) and have been assigned the identifier ‘GSE129048’.

## 3 Results

### 3.1 FGF2 negatively regulates TGF‐β2‐induced expression of myofibroblast markers in tumor endothelial cells

**Figure 1:**
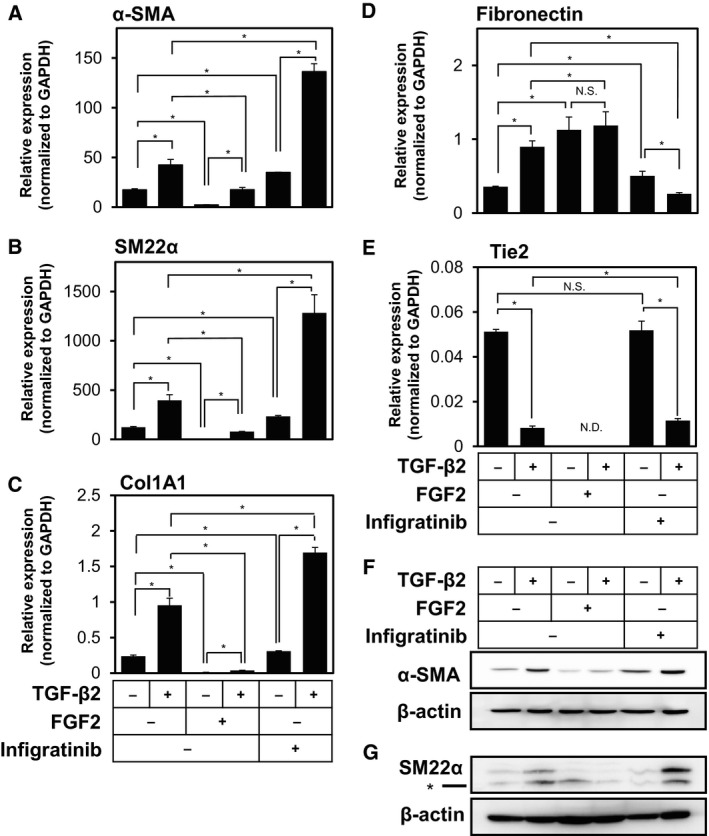
Effects of TGF‐β2, FGF2, and Infigratinib on the expression of mesenchymal markers in TECs. TECs were cultured in the absence (−) or presence (+) of 1 ng·mL^−1^ of TGF‐β2 in combination with 50 ng·mL^−1^ of FGF2 or 3 μm of Infigratinib (a pan‐inhibitor of FGF receptors) for 72 h, followed by qRT‐PCR analysis for the expression of α‐SMA (A), SM22α (B), Col1A1 (C), Fibronectin (D), and Tie2 (E) and immunoblotting analysis for the expression of α‐SMA (F), SM22α (G), and β‐actin (F, G). Concentrations of TGF‐β2 and FGF2 used were determined based on their effects on the expression of various markers (data not shown). Asterisk indicates nonspecific bands generated by anti‐SM22α antibody (G). Error bars represent standard deviation. Student's *t*‐test with two biological independent replicates was used to determine statistical significance; *P <* *0.05; N.S., not significant; N.D., not detectable.

We have previously reported that various types of endothelial cells undergo mesenchymal transition in response to TGF‐β2 signals (Kokudo *et al*., 2008; Mihira *et al*., 2012). In order to study whether TGF‐β2 induces mesenchymal transition of the primary TECs derived from human tumor xenograft model, we isolated endothelial cells from the tumors arisen from the highly metastatic A375SM human melanoma cells subcutaneously transplanted to immune‐deficient nude mice, followed by establishment of TECs (Maishi *et al*., 2016). In order to examine whether these TECs possess endothelial characteristics, we tested the levels of expression of various endothelial and mesenchymal markers and compared with those in mouse pancreatic microvascular endothelial cells (MS‐1), osteoblast precursor cells (MC3T3‐E1), and embryonic fibroblast cells (NIH/3T3; Fig. S2). Both TECs and MS‐1 cells expressed significant levels of various endothelial markers including Tie2 (Fig. S2A), VEGF receptor 2 (VEGFR2; Fig. S2B), and endoglin (Fig. S2C), which could not be observed in MC3T3‐E1 or NIH/3T3 cells. In contrast, the expression levels of mesenchymal markers, α‐SMA (Fig. S2D) and SM22α (Fig. S2E), in both TECs and MS‐1 cells were significantly lower than those in MC3T3‐E1 cells. Interestingly, expression levels of Tie2 (Fig. S2A) and VEGFR2 (Fig. S2B) in TECs were lower than those in MS‐1 cells. Furthermore, the expression levels of α‐SMA (Fig. S2D) and SM22α (Fig. S2E) in TECs were higher than those in MS‐1 cells and comparable with their expression in NIH/3T3 cells, suggesting that TECs have mesenchymal characteristics to a certain extent. While we isolated two batches of TECs, both of them exhibited similar characteristics (data not shown). Treatment of TECs with TGF‐β2 for 72 h increased the expression of myofibroblast and mesenchymal markers including α‐SMA (Figs 1A,F and S3), SM22α (Fig. 1B,G), Col1A1 (Fig. 1C), and Fibronectin (Fig. 1D), and decreased the expression of Tie2 (Figs 1E and S3), suggesting that TGF‐β signals enhanced mesenchymal transition of TECs. Of note, immunofluorescence analysis of the TECs treated with TGF‐β2 revealed that only a small subset of TECs express α‐SMA and exhibit large cell morphology (Fig. S3), suggesting that TECs consist of heterogeneous populations of endothelial cells consisting of at least two different subgroups which gives rise to α‐SMA‐positive myofibroblast‐like cells and α‐SMA‐negative non‐myofibroblastic cells (Fig. S1).

Previous reports have shown that FGF2 counteracts TGF‐β‐induced mesenchymal transition of various types of endothelial cells including primary cultured mouse endothelial cells from heart and liver (Chen *et al*., 2012, 2014, 2015), HUVECs (Correia *et al*., 2016), and TECs isolated from tumor tissues formed in the transgenic mouse models of various types of cancer (Xiao *et al*., 2015). In order to examine the effects of FGF2 on the TGF‐β‐induced EndMT in TECs, we treated them with FGF2 and Infigratinib, a pan‐inhibitor of FGF receptor kinases. As shown in Fig. 1, TGF‐β2‐induced expression of α‐SMA (Figs 1A,F and S3), SM22α (Fig. 1B,G), and Col1A1 (Fig. 1C) was suppressed by the addition of FGF2 and enhanced in the presence of Infigratinib. In contrast, TGF‐β2‐induced expression of Fibronectin was not suppressed by FGF2 (Fig. 1D). While FGF2 decreased the expression of Tie2 in TECs, Infigratinib did not affect TGF‐β2‐induced Tie2 repression (Fig. 1E). In addition, TECs exhibited tube (cord)‐forming ability, which is characteristic of endothelial cells (Fig. S4). While TGF‐β2 did not affect the tube‐forming ability of TECs, FGF2 increased these endothelial characteristics (Fig. S4). We have thus concluded that FGF2 does not counteract the TGF‐β2‐induced gain of mesenchymal characteristics or loss of endothelial characteristics. Instead, it rather inhibits TGF‐β‐induced gain of myofibroblastic characteristics (End‐MyoT), because α‐SMA, SM22α, and Col1A1 are markers of myofibroblast cells.

### 3.2 FGF2 produced by TECs suppresses the expression of α‐SMA in an autocrine/paracrine manner

**Figure 2:**
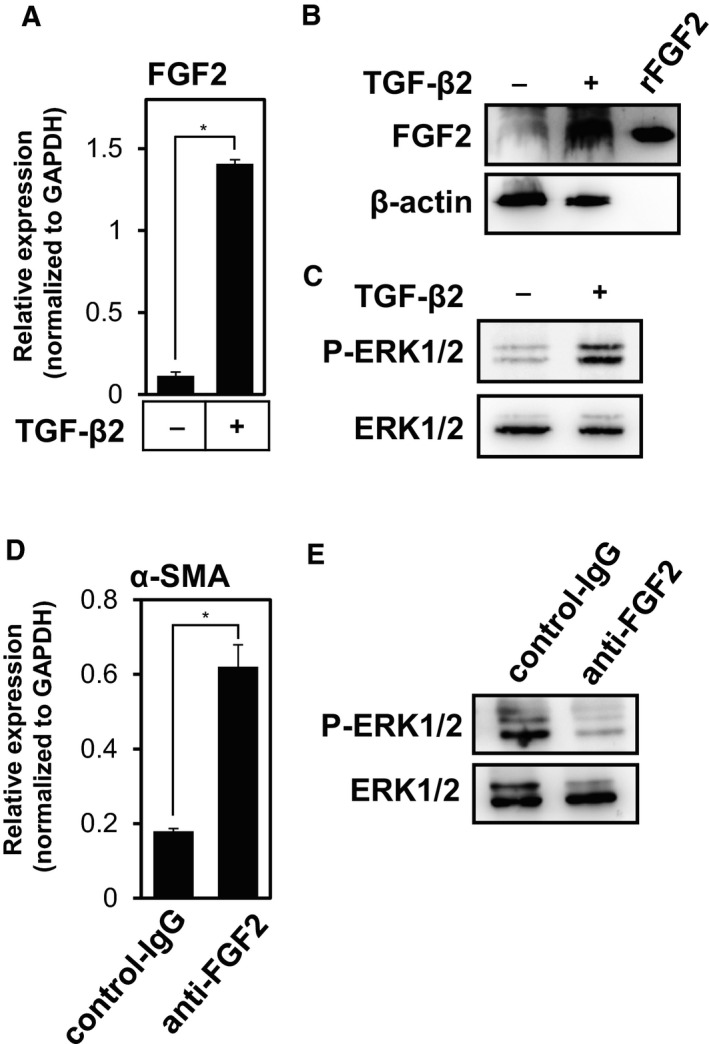
Regulation of FGF signals by TGF‐β2 in TECs. (A, B) Effect of TGF‐β2 on the expression of FGF2 in TECs. TECs were cultured in the absence (−) or presence (+) of TGF‐β2 for 72 h, followed by qRT‐PCR (A) and immunoblot (B) analyses for the expression of FGF2. rFGF2, recombinant FGF2. (C) Effect of TGF‐β2 on the ERK1/2 phosphorylation in TECs. TECs were cultured in the absence (−) or presence (+) of TGF‐β2 for 72 h, followed by immunoblot analysis using phospho‐(P)‐ERK1/2, and total ERK1/2 antibodies. (D, E) Effect of endogenous FGF2 on α‐SMA expression and the ERK1/2 phosphorylation in TECs. TECs were cultured with TGF‐β2 in combination with control‐IgG or anti‐FGF2 antibodies for 72 h, followed by qRT‐PCR analysis for α‐SMA expression (D) and immunoblot analysis using phospho‐(P)‐ERK1/2, and total ERK1/2 antibodies (E). Error bars represent standard deviation. Student's *t*‐test with two biological independent replicates was used to determine statistical significance; *P <* *0.05.

Endothelial cells secrete various soluble factors, known as angiocrine factors, and establish specialized vascular niches (Rafii *et al*., 2016). We found that TECs express a higher level of VEGF‐A than MS‐1 endothelial cells (Fig. S2F) as previously reported (Kurosu *et al*., 2011). While VEGF‐A expression was elevated by TGF‐β2 (Fig. S5A), VEGF‐A inhibited the TGF‐β2‐induced End‐MyoT and characterized by the expression of α‐SMA (Fig. S5B) and SM22α (Fig. S5C), but the effect of VEGF‐A was limited if compared to that of FGF2. In order to examine whether FGF2 acts as an angiocrine factor, we quantified the expression of FGF2 in TECs. While we were not capable of detecting the significant level of FGF2 proteins in the conditioned medium of TECs (data not shown) possibly due to unconventional fashion of its secretion (Steringer *et al*., 2015), we found that the endogenous level of FGF2 transcripts expressed by TECs was significantly elevated upon TGF‐β treatment (Fig. 2A,B), which was consistent with a previous report by Xiao and colleagues (Xiao *et al*., 2015).

In the next step, we studied whether the increased FGF2 expression induced by TGF‐β2 treatment would affect FGF signaling. We stimulated TECs with TGF‐β2 for 72 h and analyzed the phosphorylation level of ERK, a downstream signal component of the FGF signaling pathway using immunoblotting. Consistent with our present finding that TGF‐β2 increases FGF2 expression, phosphorylation of ERK1/2 was enhanced by pretreatment with TGF‐β2 (Fig. 2C).

While conditioned medium from TECs treated with TGF‐β2 for 72 h slightly decreased the TGF‐β2‐induced α‐SMA expression (data not shown), it was unclear whether endogenous FGF2 contributes to this effect. In order to examine the physiological roles of FGF2 secreted by TECs, we next treated TECs with anti‐FGF2 neutralizing antibodies (Fig. 2D). Blocking the endogenous FGF2 signals with anti‐FGF2 antibodies significantly enhanced the α‐SMA expression in TECs (Fig. 2D) and decreased the phosphorylation level of ERK (Fig. 2E), suggesting that autocrine/paracrine FGF2 signals suppressed the expression of α‐SMA in TECs.

### 3.3 FGF2 antagonizes TGF‐β2 to confer TECs with myofibroblastic properties

**Figure 3:**
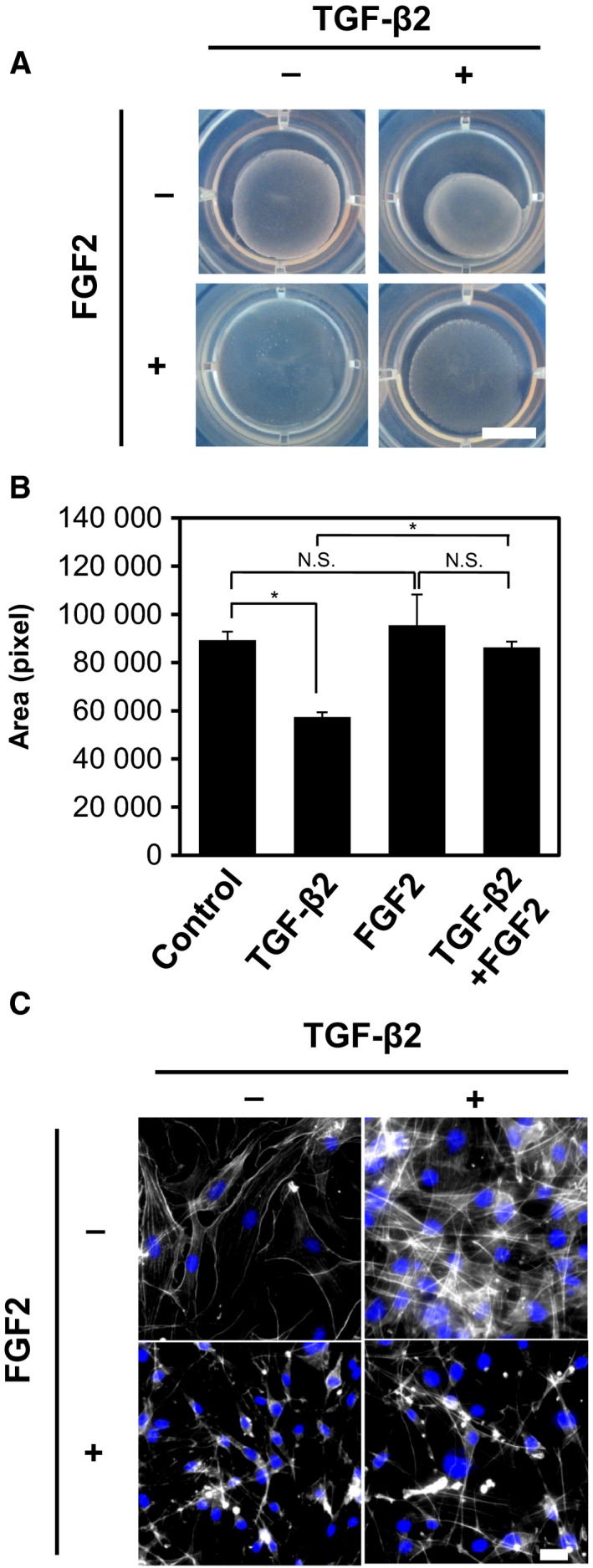
Effects of TGF‐β2 and FGF2 on the myofibroblastic properties of TECs. (A) TECs were preincubated with or without TGF‐β2, FGF2, or combination of both factors for 72 h and embedded into collagen matrices. The mixtures were released from the culture dishes, incubated for additional 2 days, and photographed. Experiments were performed in duplicate. Scale bar: 10 mm. (B) Graphic representation of the relative gel areas quantified by imagej. Error bars represent standard deviation. Student's *t*‐test with three biological independent replicates was used to determine statistical significance; *P <* *0.05; N.S., not significant. (C) TECs were preincubated with (+) or without (−) TGF‐β2, FGF2, or combination of both factors for 72 h, followed by fluorescence immunostaining for F‐actin (white) and nuclei (blue). Scale bars: 10 mm (A) and 50 μm (C).

We next attempted to examine the effects of FGF2 on the TGF‐β2‐induced acquisition of mesenchymal and myofibroblastic phenotypes. Since myofibroblasts exhibit higher contractile forces and abundant extracellular matrix production, we performed collagen gel contraction assay to study the effects of FGF2 on TGF‐β2‐induced contractile ability of TECs. A significant gel contraction was observed in TGF‐β2‐treated TECs if compared to the control cells (Fig. 3A,B). However, treatment of TECs with combination of TGF‐β2 and FGF2 significantly reduced contraction induced by TGF‐β2, while FGF2 alone did not alter their contractile ability (Fig. 3A,B). Furthermore, we found that TGF‐β2 induced a change in morphology of TECs toward a more widespread shape along with the formation of pronounced actin stress fibers, characteristics of myofibroblasts (Fig. 3C). These pro‐myofibroblastic effects of TGF‐β2 were partially inhibited by treatment with FGF2 (Fig. 3C). Taken together with the present finding that FGF2 suppresses TGF‐β2‐induced expression of myofibroblast markers (Fig. 1), these results suggest that FGF2 counteracts the TGF‐β2‐induced myofibroblast transition of TECs.

### 3.4 FGF2 cooperates with TGF‐β2 to enhance the motility and proliferation of TECs

**Figure 4:**
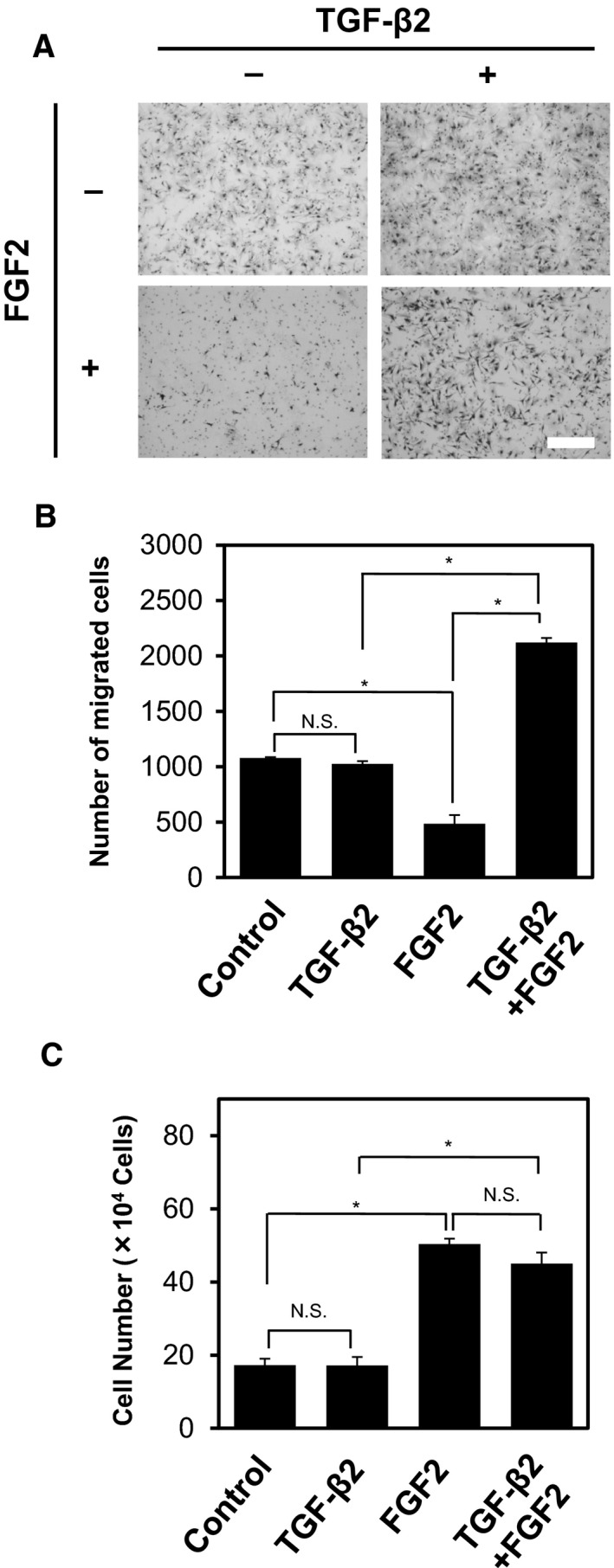
Effects of TGF‐β2 and FGF2 on the migration and proliferation of TECs. (A, B) TECs were preincubated with (+) or without (−) TGF‐β2, FGF2, or combination of both factors for 72 h, followed by chamber migration assay. Cells were allowed to migrate for 6 h, and cells migrated to the bottom side of the chamber were stained (A) and counted under a phase‐contrast microscope (B). Scale bar: 300 μm. (C) TECs were cultured with or without TGF‐β2, FGF2, or combination of both factors for 72 h, followed by direct counting of cell number. Note that treatment of TECs with TGF‐β2, FGF2, or combination of both factors for 6 h did not affect their number (data not shown). Error bars represent standard deviation. Student's *t*‐test with three biological independent replicates was used to determine statistical significance; *P <* *0.05; N.S., not significant.

Transforming growth factor‐β induces mesenchymal transition of epithelial cells, termed EMT. One of the mesenchymal characteristics that is conferred to the TGF‐β‐treated epithelial cells is an increased cell motility. Since we have previously reported that FGF2 enhanced the TGF‐β‐induced motility of NMuMG mouse mammary gland epithelial cells (Shirakihara *et al*., 2011), we examined whether FGF2 elicits similar effects on TECs. TECs were exposed to TGF‐β2, FGF2, or both factors for 72 h, and were subjected to chamber migration assay. TECs treated with TGF‐β2 alone did not show significantly enhanced cell motility, if compared with nontreated cells (Fig. 4A,B). On the other hand, treatment of TECs with TGF‐β2 and FGF2 strongly promoted the motility of the cells, while treatment with FGF2 alone decreased TEC motility. In addition, treatment of TECs with FGF2 increased their number, while TGF‐β2 did not affect their proliferation (Fig. 4C), suggesting that FGF2 induced the formation of active fibroblasts. These findings suggest that the cells exposed to TGF‐β2 and FGF2 exhibit phenotypes that are shared by active fibroblasts, but not by myofibroblasts.

### 3.5 FGF2 differentially regulates the expression of TGF‐β2‐regulated genes in TECs

Our data suggested that FGF2 differentially regulates the TGF‐β2‐induced mesenchymal phenotypes. While FGF2 suppresses the TGF‐β2‐induced myofibroblast transition marked by increased expression of myofibroblast markers (Fig. 1) and contractile activity (Fig. 3), FGF2 enhances TEC motility and proliferation (Fig. 4). Based on these observations, we expected that FGF2 confers migratory phenotypes to TGF‐β2‐treated TECs by suppressing their myofibroblastic phenotypes.

**Figure 5:**
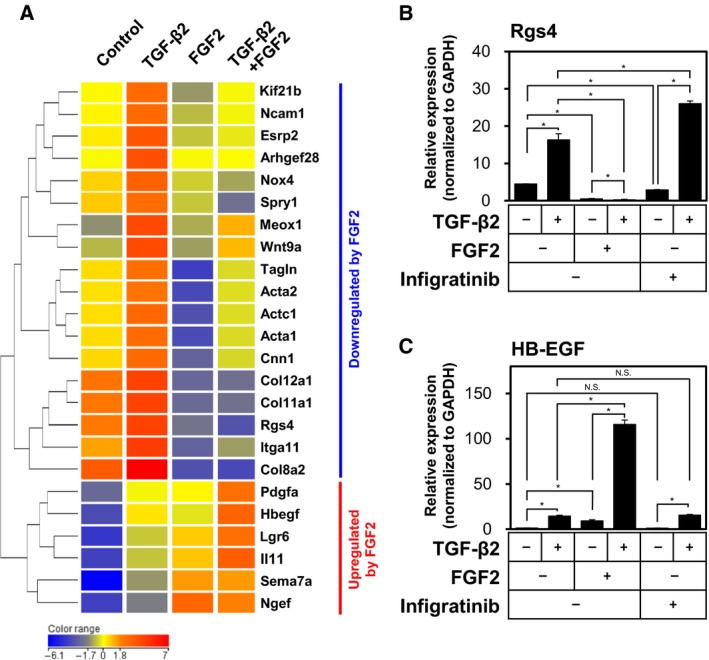
Differential effects of FGF2 on the TGF‐β2‐mediated expression of various markers in TECs. (A) Heatmap and hierarchical clustering of gene expression in TECs treated with TGF‐β2, FGF2, or combination of both factors and analyzed using the Agilent Expression Array data. Results were normalized and log‐transformed. Genes were clustered using the hierarchical method. (B, C) TECs were cultured in the absence (−) or presence (+) of TGF‐β2 in combination with FGF2 or Infigratinib for 72 h, followed by qRT‐PCR analysis for the expression of Rgs4 (B) and HB‐EGF (C). Error bars represent standard deviation. Student's *t*‐test with two biological independent replicates was used to determine statistical significance; *P <* *0.05; N.S., not significant.

To comprehensively elucidate the molecular mechanisms underlying this regulation, we performed cDNA microarray analysis using cDNA samples from TECs treated with TGF‐β2, FGF2, or both factors for 72 h. As shown in Tables S2, S3 and Figs 5A and S6, FGF2 decreased the expression of a group of TGF‐β‐upregulated genes, but at the same time increased the expression of other group of genes. FGF2 counteracted TGF‐β2‐induced expression of various myofibroblast markers including α‐SMA (Acta2), calponin (Cnn1), SM22α (Tagln), NADPH oxidase 4 (Nox4), and regulator of G protein signaling 4 (Rgs4; Fig. 5A) (Augsten, 2014; Sampson *et al*., 2011). These results were confirmed by an additional set of cDNA microarray analysis using cDNA samples from TECs treated with TGF‐β2 in combination with FGF2 or Infigratinib for 72 h (Fig. S7 and Table S4). TGF‐β2‐induced expression of Rgs4 was suppressed by FGF2 and enhanced by Infigratinib (Figs 5B and S7), which was similar to the effect of FGF2 on α‐SMA expression (Fig. 1A). In contrast, FGF2 augmented TGF‐β2‐induced expression of various growth factors including PDGF‐A (Pdgfa) and heparin‐binding EGF‐like growth factor (HB‐EGF; Hbegf), which have been implicated in the activation of angiogenesis (Fig. S7). These results were confirmed by qRT‐PCR; TGF‐β2‐induced expression of HB‐EGF was enhanced by FGF2 and suppressed by Infigratinib (Fig. 5C). These results suggest that FGF2 elicits dual effects on TGF‐β2‐induced EndMT, that is, suppression of myofibroblast differentiation and augmentation of migration, through differential regulation of distinct groups of TGF‐β‐regulated genes.

### 3.6 TECs treated with TGF‐β2 are more competent in enhancing tumor growth than TECs treated with TGF‐β2 and FGF2

**Figure 6:**
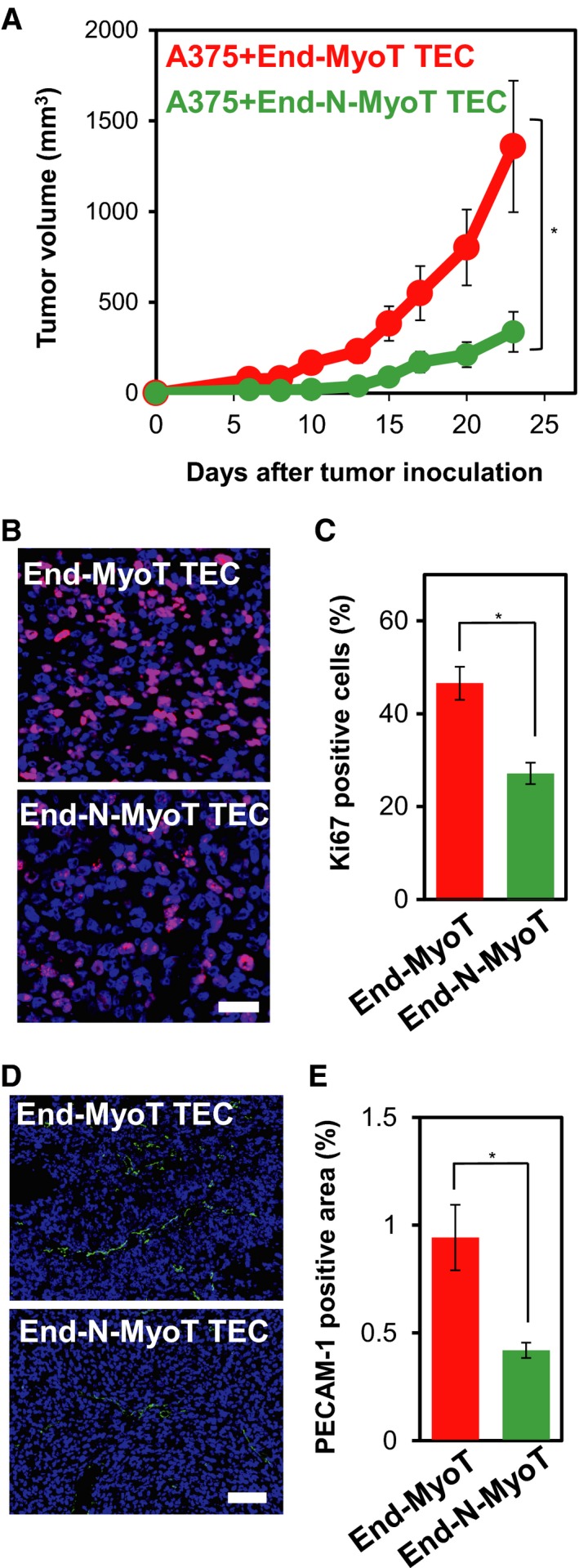
Roles of TECs treated with TGF‐β and FGF2 in the *in vivo* tumor formation of the A375 human melanoma cell. (A) TECs pretreated either with TGF‐β2 (End‐MyoT TECs) or combination of TGF‐β2 and FGF2 (End‐N‐MyoT TECs) for 72 h were mixed with A375 human melanoma cells in a 3 : 10 ratio and subcutaneously inoculated into immunodeficient mice. Tumor growth was measured using calipers and calculated from minor axis and major radius. One‐way ANOVA followed by the Student–Newman–Keuls test with four (End‐MyoT TEC group) and six (End‐N‐MyoT TEC group) biological independent replicates was used to determine statistical significance (A); *P <* *0.05. (B–E) Sections of tumors were subjected to immunofluorescence staining with the anti‐Ki67 (purple: B) and anti‐PECAM‐1 antibodies (D: green). Nuclei were counterstained with Hoechst 33342 (blue). Scale bars: 50 μm (B) and 100 μm (D). Levels of proliferation (C) and angiogenesis (E) were quantified. Error bars represent standard error. Student's *t*‐test with twelve (C) and twenty (E) biological independent replicates was used to determine statistical significance (C, E); *P <* *0.05.

Stromal compartment of many types of malignant human cancers contains a large number of myofibroblasts, which express α‐SMA (Mezawa and Orimo, 2016). In order to examine the contribution of TEC‐derived myofibroblasts and active fibroblasts to *in vivo* tumor growth, we mixed the TECs treated with TGF‐β2 (termed End‐MyoT TECs) or those treated with TGF‐β2 and FGF2 (termed End‐N‐MyoT TECs) with A375 human melanoma cells in a 3 : 10 ratio and grafted these mixtures subcutaneously into the immunodeficient mice. As shown in Fig. 6A, the A375 cells mixed with End‐MyoT TECs formed the tumors of greater volume than those mixed with End‐N‐MyoT TECs. The tumors that developed in the presence of End‐MyoT TECs also contained more proliferating carcinoma cells than the tumors developing in the presence of End‐N‐MyoT TECs (Fig. 6B,C), indicating increased tumor cell proliferation.

Previous reports showed that α‐SMA‐expressing CAFs derived from invasive human breast cancer promote the growth of tumors via inducing tumor angiogenesis using a human breast cancer xenograft model (Orimo *et al*., 2005). In order to examine the possibility that End‐MyoT TECs stimulated tumor angiogenesis, we studied the formation of tumor‐associated vasculature by staining the sections from xenograft tumors using anti‐PECAM‐1 antibody (Fig. 6D,E). While extensive vascular formation was observed in tumors containing End‐MyoT TECs, capillaries in tumors containing End‐N‐MyoT TECs were far less developed (Fig. 6D,E). Taken together, these results suggested that End‐MyoT TECs enhanced tumor growth, at least in part, by promoting tumor angiogenesis.

### 3.7 Elk1 transcription factor suppresses TGF‐β2‐induced expression of myofibroblast markers in TECs

**Figure 7:**
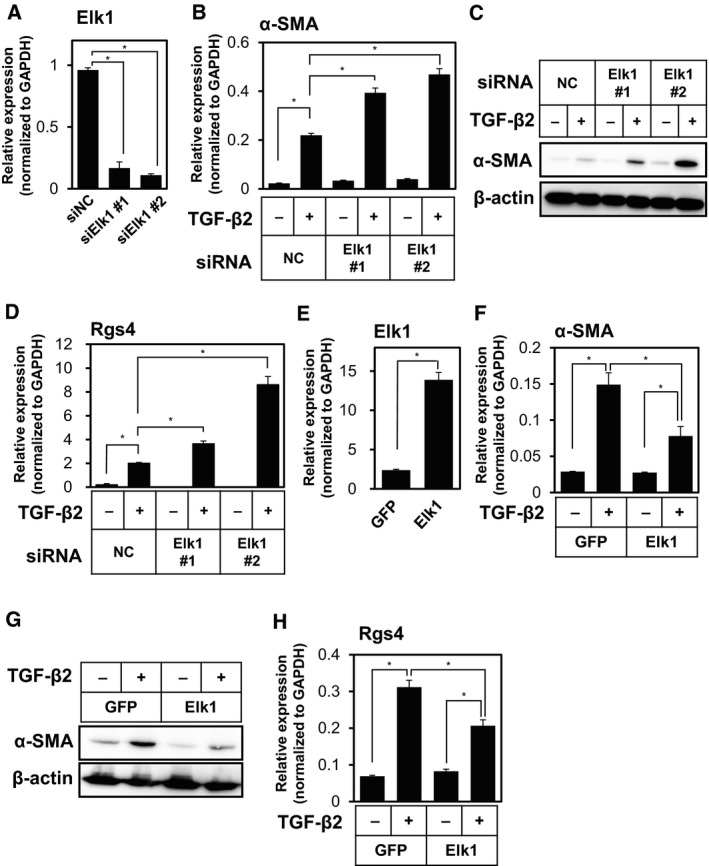
Effects of Elk1 on the TGF‐β2‐induced expression of myofibroblast markers in TECs. (A) TECs were transfected with negative control siRNA (NC) or siRNA for Elk1 (siElk1 #1 and #2). The expression of Elk1 was determined by qRT‐PCR analysis. (B–D) TECs transfected with siRNA were cultured in the absence (−) or presence (+) of TGF‐β2 for 72 h, followed by qRT‐PCR analysis for the expression of α‐SMA (B) and Rgs4 (D), and immunoblot analysis for the expression of α‐SMA and β‐actin (C). (E–H) TECs were infected with lentivirus encoding GFP as control or Elk1, followed by qRT‐PCR analysis for the expression of Elk1 (E). Elk1‐expressing or control TECs were cultured in the absence (−) or presence (+) of TGF‐β2 for 72 h, followed by qRT‐PCR analysis for the expression of α‐SMA (F) and Rgs4 (H), and immunoblot analysis for the expression of α‐SMA and β‐actin (G). Error bars represent standard deviation. Student's *t*‐test with two biological independent replicates was used to determine statistical significance; *P <* *0.05.

In order to elucidate the mechanisms by which FGF2 regulates the TGF‐β2‐induced α‐SMA expression in TECs, we searched for candidate molecules that could be involved in regulation of TGF‐β2‐induced End‐N‐MyoT in response to FGF2 signals. Our cDNA microarray analysis led us to the identification of a group of genes (α‐SMA, Calponin, SM22α, and Nox4) whose expression is directly regulated by SRF and the myocardin family transcription factors (Hecker *et al*., 2009; Sampson *et al*., 2011). Since transcriptional activation of SMC‐related genes by the myocardin family transcription factors is competed by Elk1 (Wang *et al*., 2004), we examined whether TGF‐β2‐induced expression of myofibroblast markers in endothelial cells could be affected by the altered expression of Elk1 transcription factor. To study the effects of loss of function of Elk1 on TGF‐β‐induced expression of α‐SMA and Rgs4, we knocked down Elk1 expression by specific siRNA (Fig. 7A) and examined the expression of α‐SMA (Fig. 7B,C) and Rgs4 (Fig. 7D). TGF‐β2‐induced expression of α‐SMA and Rgs4 was enhanced by decreased Elk1 expression in TECs, suggesting that endogenous Elk1 is required for the suppression of TGF‐β‐induced mesenchymal marker expression in endothelial cells. In contrast, the effects of decreased Elk1 expression on TGF‐β2‐induced expression of HB‐EGF varied depending on the siRNA (data not shown), suggesting that endogenous Elk1 is not likely involved in the regulation of the other group of TGF‐β target genes. Furthermore, when Elk1 was ectopically expressed in TECs (Fig. 7E), TGF‐β2‐induced expression of α‐SMA (Fig. 7F,G) and Rgs4 (Fig. 7H) in TECs decreased. These results suggest that Elk1 is necessary and sufficient for the suppression of TGF‐β2‐induced expression of myofibroblast markers but not of active fibroblast markers.

### 3.8 FGF2 facilitates Elk1 to antagonize the transcriptional activity of MRTF‐A to regulate TGF‐β2‐induced α‐SMA expression

**Figure 8:**
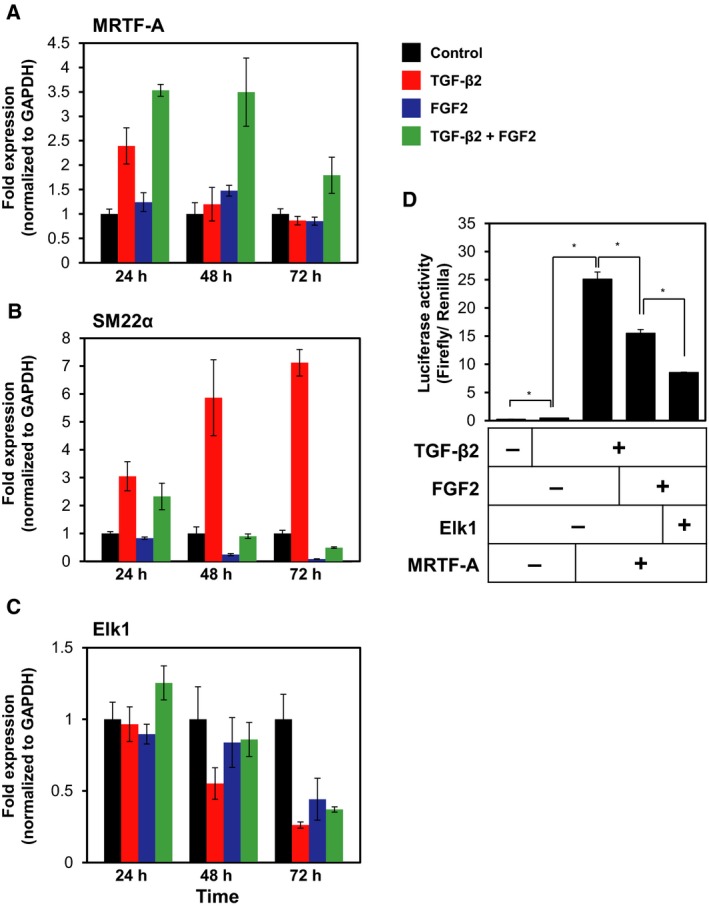
Roles of TGF‐β2, FGF2, Elk1, and MRTF‐A during myofibroblast transition of TECs. (A–C) TECs were cultured with or without TGF‐β2, FGF2, or combination of both factors for the indicated periods, followed by qRT‐PCR analysis for the expression of MRTF‐A (A), α‐SMA (B), and Elk1 (C). (D) MS‐1 cells were transfected with a luciferase reporter construct containing the α‐SMA promoter fragment along with expression construct encoding MRTF‐A and/or Elk1, in the presence or absence of TGF‐β2 and FGF2, followed by the measurement of luciferase activity. Error bars represent standard deviation. Student's *t*‐test with two biological independent replicates was used to determine statistical significance; *P <* *0.05.

We previously reported that TGF‐β2‐induced expression of MRTF‐A was necessary and sufficient to induce α‐SMA expression in MS‐1 endothelial cells (Mihira *et al*., 2012). In order to examine the causal relationship between TGF‐β2‐induced expression of MRTF‐A and various myofibroblast markers, we performed kinetic study of the effects of TGF‐β2 and FGF2 on the expression of the factors involved in End‐MyoT and End‐N‐MyoT (Figs 8A–C and S8). Induction of MRTF‐A expression (Fig. 8A) by TGF‐β2 preceded the expression of various myofibroblast markers, including SM22α (Fig. 8B), α‐SMA (Fig. S8A), and Rgs4 (Fig. S8B), suggesting that the expression of myofibroblast markers was upregulated by TGF‐β2‐induced MRTF‐A. Furthermore, we found that TGF‐β2 decreased the expression of Elk1 (Fig. 8C), which may lead to the increase in the expression of myofibroblast markers (Fig. 8B). Of note, FGF2 expression was induced by TGF‐β2 in a similar manner with that of MRTF‐A (Fig. S8C).

Fibroblast growth factors, like PDGFs, transduce their signals by activation of MEK/ERK pathways, which leads to activation of Elk1 as previously reported (Morita *et al*., 2007). In order to examine whether the FGF2‐mediated Elk1 activation interferes with the transcriptional activation of α‐SMA gene by TGF‐β2‐induced MRTF‐A expression, we performed reporter activity assays using the promoter region (the 724 bp fragment of 5′‐flanking region) of α‐SMA gene consisting of two CArG boxes that contribute to its MRTF‐A‐mediated transcription (Mihira *et al*., 2012). When MS‐1 endothelial cells, transfected with a luciferase reporter construct containing the α‐SMA promoter fragment, were cultured in the absence or presence of TGF‐β2, the reporter activity was enhanced by TGF‐β2 (Fig. 8D). This TGF‐β2‐induced activation of α‐SMA promoter was further enhanced by MRTF‐A, suggesting that TGF‐β2–MRTF‐A axis enhances α‐SMA transcription. However, the α‐SMA promoter activity decreased by the treatment of MS‐1 cells with FGF2 and expression of Elk1 (Fig. 8D). These results suggest that two transcription factors, MRTF‐A and Elk1, are activated by TGF‐β2 and FGF2, respectively, and compete with each other in forming a complex with SRF to regulate the expression of myofibroblast markers in endothelial cells.

## 4 Discussion

We have previously reported that TGF‐β‐induced EMT results in the formation of fibroblastic cells from epithelial cells. Such fibroblastic cells can further undergo EMyoT and differentiate into myofibroblast cells by long exposure to TGF‐β, or in the presence of both FGF2 and TGF‐β undergo EMT resulting in the formation of activated fibroblastic cells (Shirakihara *et al*., 2011). In the present study, we have demonstrated that auto/paracrine FGF2 signals in TECs suppress TGF‐β‐induced End‐MyoT (Figs 1, 2, 3) and promote the formation of migratory mesenchymal cells (Fig. 4). Xiao and Dudley (2017)recently proposed a model in which heterogeneous populations of endothelial cells consisting of at least two different subgroups undergo distinct types of TGF‐β‐stimulated EndMT. One subpopulation of endothelial cell gives rise to α‐SMA‐positive myofibroblastic cells, while the other one produces α‐SMA‐negative active fibroblastic cells. TGF‐β induces the formation of both α‐SMA‐positive myofibroblasts (End‐MyoT) and α‐SMA‐negative active fibroblasts (End‐N‐MyoT; Fig. S9A), whereas FGF2 suppresses the effects of TGF‐β during these processes and enhances the migration of activated fibroblastic cells (Fig. S9B). While Xiao *et al*. (2015) used TECs isolated from tumor tissues formed in the transgenic mouse models, the presence of heterogeneous populations in TECs derived from xenograft model of human cancer cells used in the present study needs to be studied by single cell analyses in the future.

In the present study, we showed that TGF‐β and FGF2 either cooperate with each other or oppose each other during End‐N‐MyoT and End‐MyoT through transcriptional regulation of arrays of various mesenchymal/myofibroblast markers and growth factors (Fig. 5). We also attempted to identify the FGF2‐regulated genes whose expression levels are modulated by TGF‐β2 using the microarray data (Fig. S10 and Table S6). We found that the expression of a group of genes including MMP8 and Netrin1, that have been implicated in the maintenance of endothelial cell characteristics (Castets *et al*., 2009; Fang *et al*., 2013), was increased by FGF2 but decreased in the presence of TGF‐β2. In contrast, we found that the expression of a group of genes including DOCK8, that has been implicated in cell migration (Harada *et al*., 2012), increased by combined treatment with FGF2 and TGF‐β2 (Fig. S10). Among them, the expression pattern of HB‐EGF is intriguing. While FGF2 alone upregulated HB‐EGF expression, FGF2 together with TGF‐β2 further induced HB‐EGF expression (Fig. 5C). In order to further study the regulation of HB‐EGF expression, we treated TECs with TGF‐β2 or FGF2 in the absence or presence of SB431542, an inhibitor of TGF‐β type I receptor. While the TGF‐β2‐induced SM22α expression was abrogated by SB431542 (Fig. S11A), the FGF2‐induced HB‐EGF expression was not decreased by SB431542 (Fig. S11B), suggesting that the expression of HB‐EGF in TECs depends mainly on FGF2 signals and becomes enhanced by TGF‐β2. We also found that the expression of Sprouty1 (Spry1) is increased by TGF‐β2 but decreased by FGF2 in TECs (Fig. 5A and Table S2). Since Spry1 is an inhibitor of FGF signaling (Mason *et al*., 2006), these results, together with present finding that TGF‐β2 increases FGF2 expression (Fig. 2), suggest that the interplay between TGF‐β and FGF2 signals may play important roles during mesenchymal transition of TECs.

In addition, by performing loss‐ and gain‐of‐function studies, we identified Elk1 as a factor responsible for FGF2‐induced inhibition of End‐MyoT (Fig. 7). Furthermore, we found that Elk1 repressed the α‐SMA promoter activity in the presence of FGF2 (Fig. 8D). Previous reports revealed that Elk1 becomes activated by PDGF signals and competes with myocardin family transcription factors for the interaction with SRF during the induction of α‐SMA expression (Besnard *et al*., 2011). Since Elk1 contains an evolutionarily conserved repression domain (Yang *et al*., 2002), Elk1 may also play as a transcriptional repressor. Molecular mechanisms how Elk1 regulates the expression of myofibroblast markers remain to be elucidated in the future. We also previously identified miR‐27 as a positive regulator of TGF‐β‐induced EndMT (Suzuki *et al*., 2017). Because one of the targets of miR‐27 is Elk1, miR‐27 appears to play important roles in TGF‐β‐induced EndMT by downregulating the function of Elk1. Taken together with our previous findings that TGF‐β‐dependent activation of Rho signals induces EndMT via MRTF‐A, TGF‐β/Rho/MRTF‐A and FGF2/MEK–ERK/Elk1 axes compete with SRF to control expression of mesenchymal markers in endothelial cells (Fig. S9).

Recent studies have shown that cross talk between tumor cells and stromal cells plays an important role in tumor progression. We have previously reported that TECs isolated from highly metastatic tumors have more pro‐angiogenic characteristics than those isolated from low metastatic tumors (Ohga *et al*., 2012). Furthermore, TECs isolated from highly metastatic tumors actively promoted tumor metastasis through mutual interaction with tumor cells (Maishi *et al*., 2016). These characteristics of TECs are mediated by biglycan, whose expression is upregulated by DNA demethylation. These results suggest that TECs are epigenetically altered in their microenvironment to produce TEC‐specific angiocrine factors including FGF2. We also found that the co‐transplantation of the TGF‐β2‐treated TECs (End‐MyoT TECs) with A375 human melanoma cells to immunodeficient mice resulted in the formation of tumors with a greater size than the TECs treated with TGF‐β2 and FGF2 (End‐N‐MyoT TECs; Fig. 6), suggesting that TECs that are conferred with myofibroblastic properties behave as the tumor‐promoting CAFs. Elucidating the mechanisms by which TECs acquire their phenotypes will lead to a better understanding of the mechanisms of tumor progression and the development of new therapeutic strategies.

Endothelial‐to‐mesenchymal transition has been implicated in various steps of tumor progression (Xiao and Dudley, 2017). For example, EndMT contributes to the formation of CAFs, which has been known to facilitate the proliferation of cancer cells and the progression of multiple types of cancer. Zeisberg *et al*. (2007) reported that up to 40% of CAFs originated through EndMT in mouse cancer models. Recent reports have shown that it plays important roles in tumor metastasis (Gasparics *et al*., 2016). Mouse tumor models deprived of endoglin, a co‐receptor for TGF‐β in endothelial cells, exhibited increased metastatic ability accompanied by the EndMT, suggesting that elevated vascular permeability during this process allows extravasation and intravasation of tumor cells (Anderberg *et al*., 2013). Furthermore, an increasing line of evidence has demonstrated the importance of EndMT in other pathological situations including organ fibrosis (Li *et al*., 2009; Widyantoro *et al*., 2010; Zeisberg *et al*., 2007, 2008), atherosclerosis (Chen *et al*., 2015), pulmonary arterial hypertension (Jimenez and Piera‐Velazquez, 2016), and cerebral cavernous malformation (Maddaluno *et al*., 2013). In wound‐healing models and in clinical studies, administration of FGF2 reduced scar tissue and inhibited the pro‐fibrogenic effect of TGF‐β (Akita *et al*., 2008; Eto *et al*., 2012; Ono *et al*., 2007), indicating that the therapeutic benefits of FGF2 are likely due to the inhibition of TGF‐β‐induced formation of myofibroblastic fibroblasts which correspond to type II CAF in case of tumorigenesis. Therefore, elucidation of the molecular mechanisms of FGF2 action during End‐MyoT and End‐N‐MyoT may eventually contribute to the development of therapeutic strategies for cancer and other pathological situations.

## 5 Conclusions

The present study, for the first time, demonstrated that TGF‐β and FGF2 oppose and cooperate with each other during the formation of various types of mesenchymal cells from TECs, which contributes to the characteristics of tumor microenvironment.

## Conflict of interest

YA is an employee of Nippon Kayaku, Co., Ltd. All other authors declare that they have no competing interests.

## Author contributions

TW and KM conceived, designed, and analyzed experiments and wrote the manuscript; YA, NT, YY, SK, and AK performed experiments; YA, YY, NM, KH, TM, HIS, and JI helped in conceiving and/or analyzing the experiments and provided reagents.

## Supporting information


**Fig. S1**. Two modes of TGF‐β‐induced mesenchymal transition of TECs.
**Fig. S2**. Expression of various endothelial and mesenchymal markers in multiple types of endothelial and mesenchymal cells.
**Fig. S3**. Effects of TGF‐β2, FGF2 and Infigratinib on the expression of endothelial and myofibroblast markers in TECs.
**Fig. S4**. Effects of TGF‐β2 and FGF2 on the tube forming ability of TECs.
**Fig. S5**. Effects of TGF‐β2 and VEGF‐A on the expression of mesenchymal markers in TECs.
**Fig. S6**. Differential effects of FGF2 on the TGF‐β2‐mediated expression of various markers in TECs.
**Fig. S7**. Differential effects of FGF2 and Infigratinib on the TGF‐β2‐mediated expression of various markers in TECs.
**Fig. S8**. Effects of TGF‐β2 and FGF2 on the expression of mesenchymal markers and FGF2 in TECs.
**Fig. S9**. Roles of TGF‐β2 and FGF2 signals in the regulation of End‐MyoT and End‐N‐MyoT of TECs.
**Fig. S10**. Differential effects of TGF‐β2 on the FGF2‐mediated expression of various markers in TECs.
**Fig. S11**. Effects of TGF‐β2, FGF2, and SB431542 on the expression of mesenchymal markers in TECs.

**Table S1**. Primers used for RT‐PCR.
**Table S2**. List of genes whose expression is upregulated by TGF‐β2 and further modulated by FGF2 in combination with TGF‐β2.
**Table S3**. List of genes whose expression is downregulated by TGF‐β2 and further modulated by FGF2 in combination with TGF‐β2.
**Table S4**. List of genes whose expression is upregulated by TGF‐β2 and further modulated by Infigratinib in combination with TGF‐β2.
**Table S5**. List of genes whose expression is downregulated by TGF‐β2 and further modulated by Infigratinib in combination with TGF‐β2.
**Table S6.** List of genes whose expression is regulated by FGF2 and further modulated by TGF‐β2 in combination with FGF2.
