# Supplementary figures and images for: Fibroblast growth factor signals regulate transforming growth factor‐β‐induced endothelial‐to‐myofibroblast transition of tumor endothelial cells via Elk1
Source: Mol Oncol. 2019 Jun 19;13(8):1706–24. doi: 10.1002/1878-0261.12504 (PMC6670013; doi:10.1002/1878-0261.12504)

Fig. S1

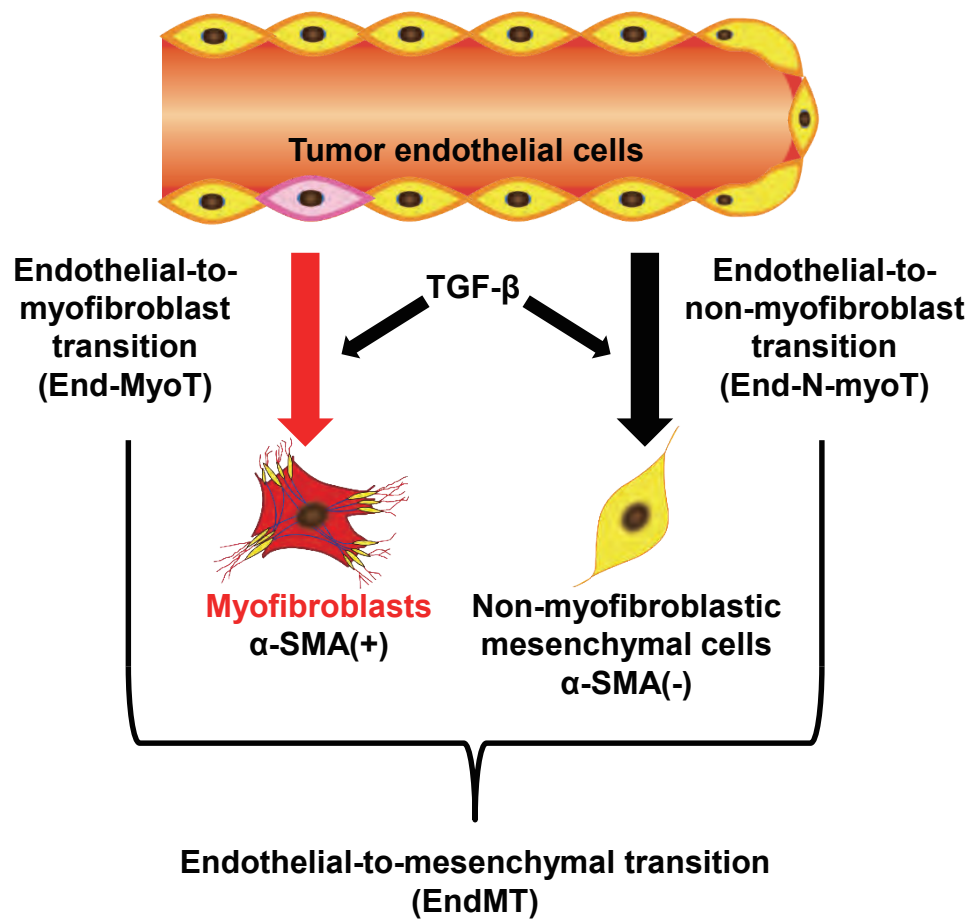

**Fig. S2**

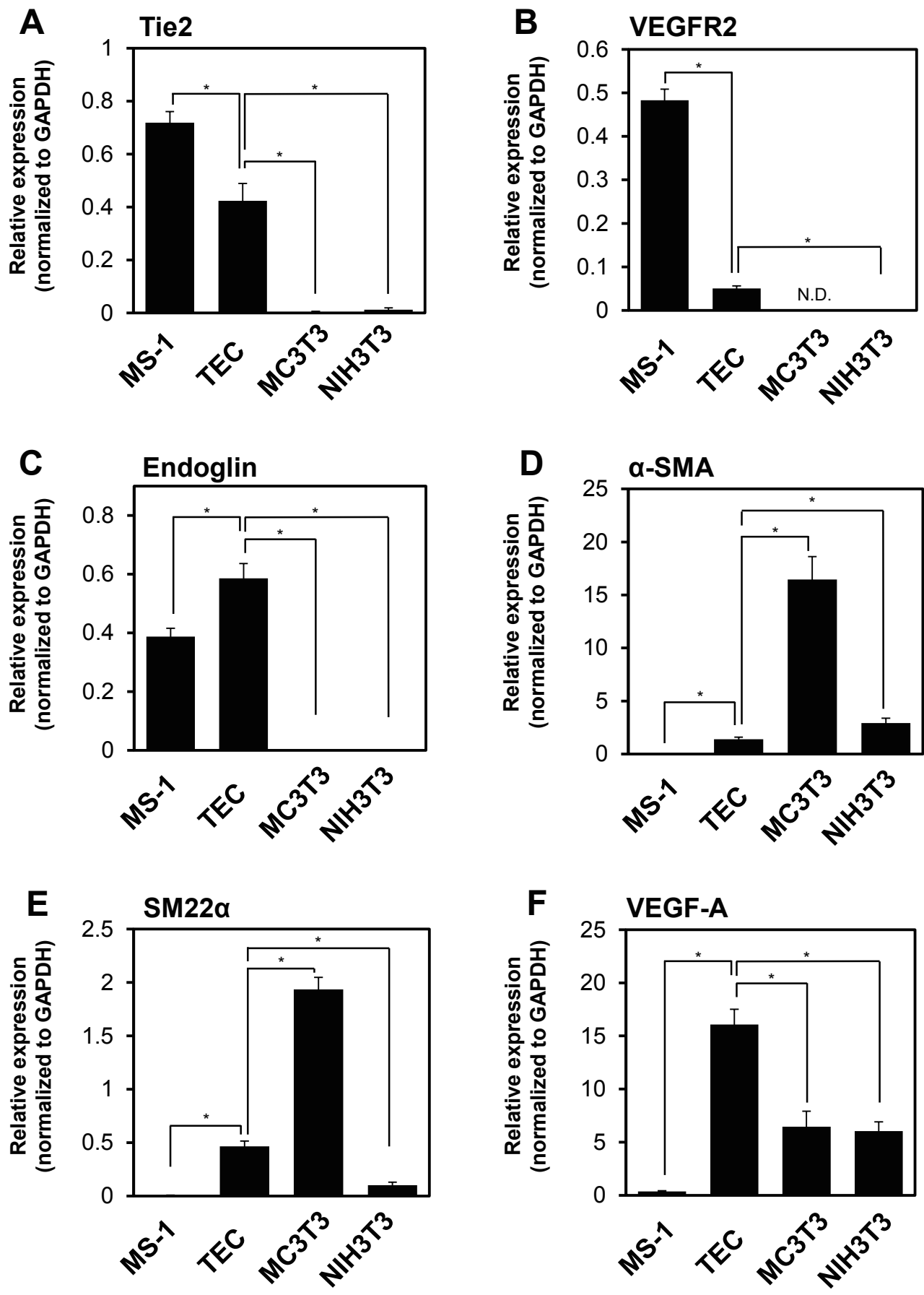

Fig. S3

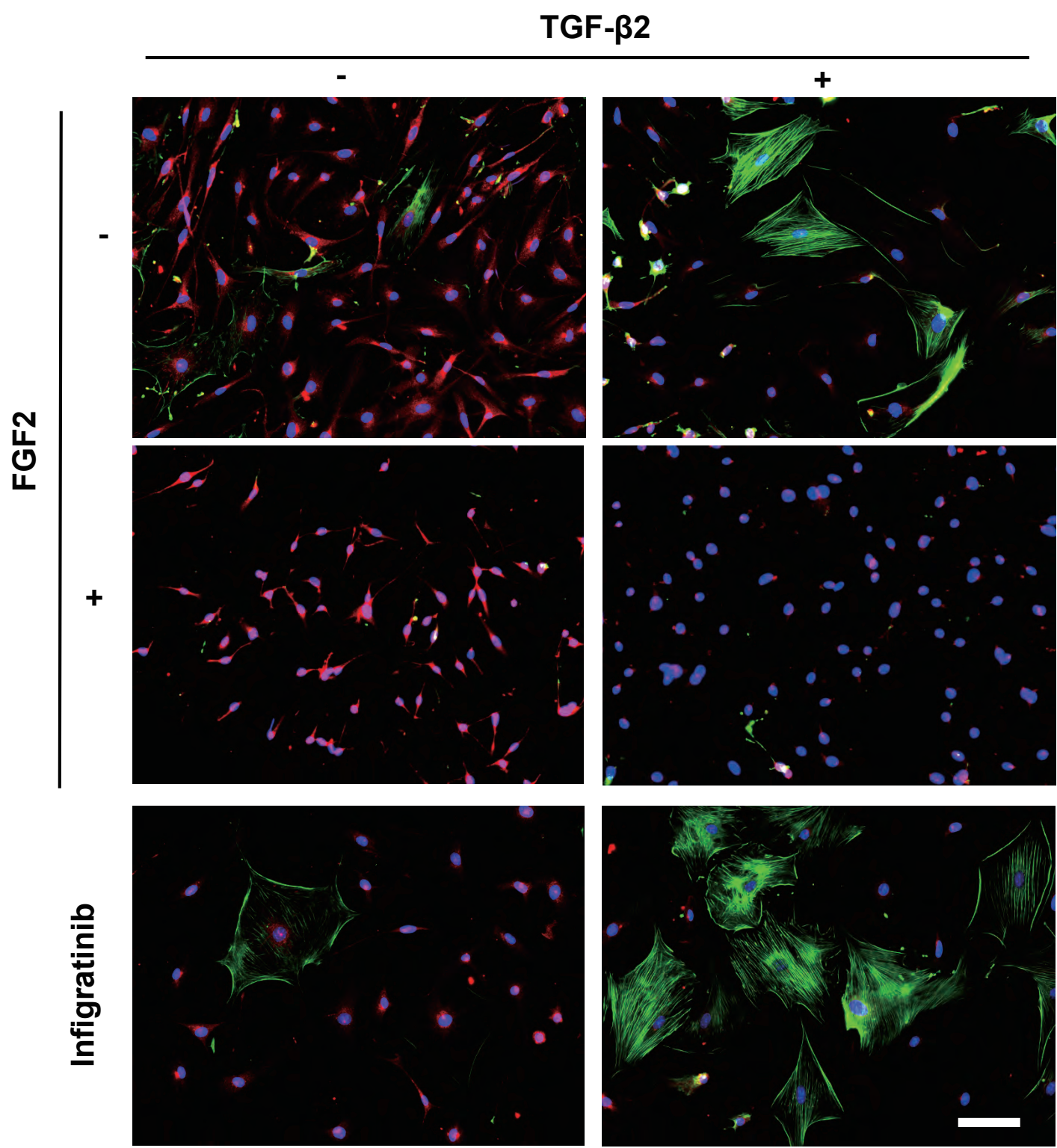

Fig. S4

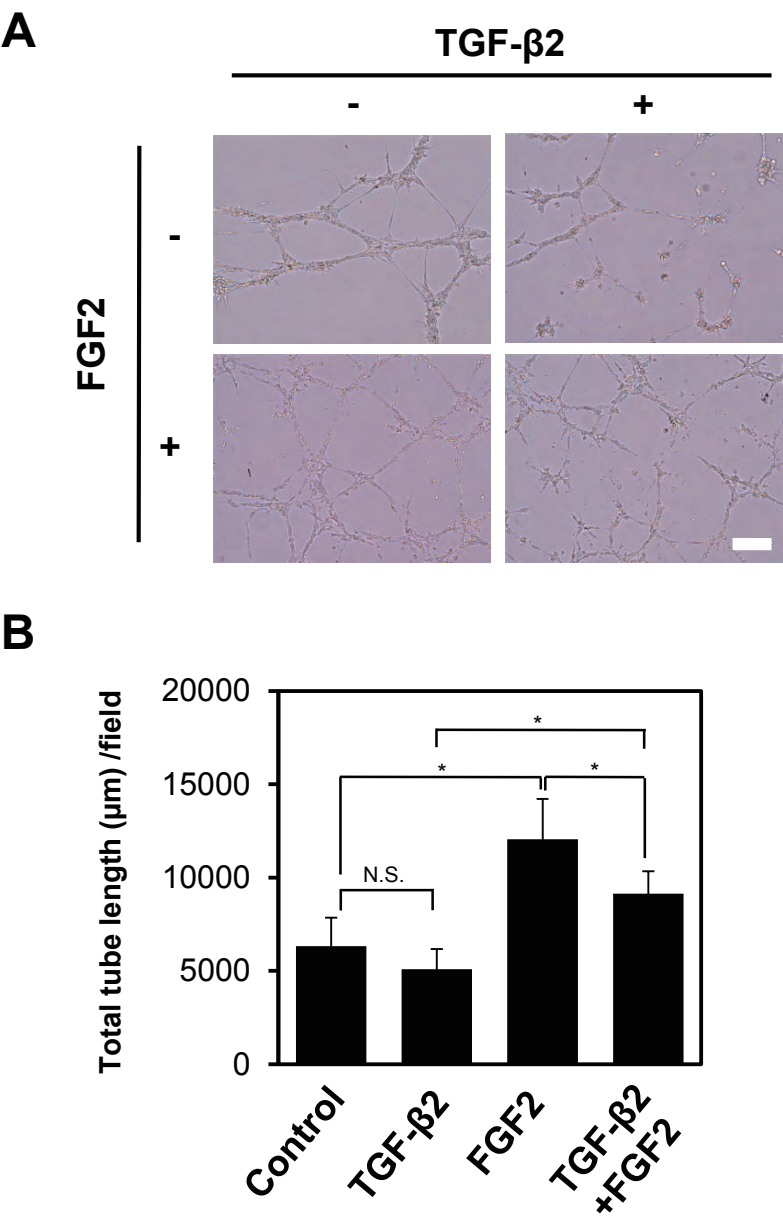

Fig. S5

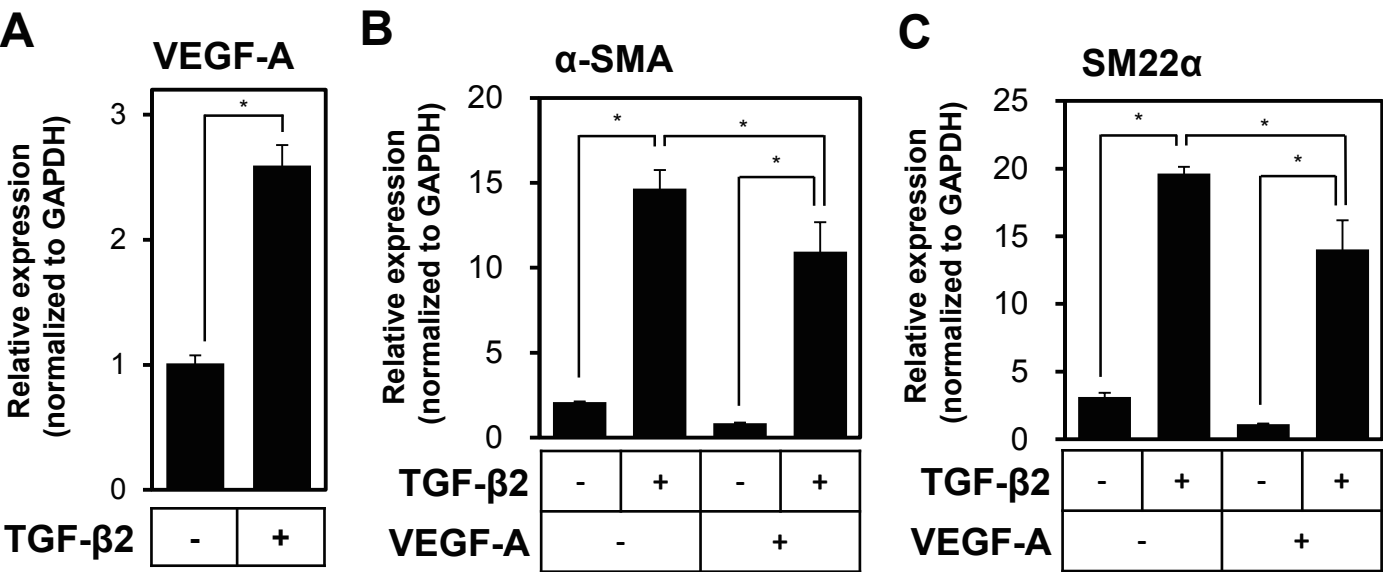

Fig. S6

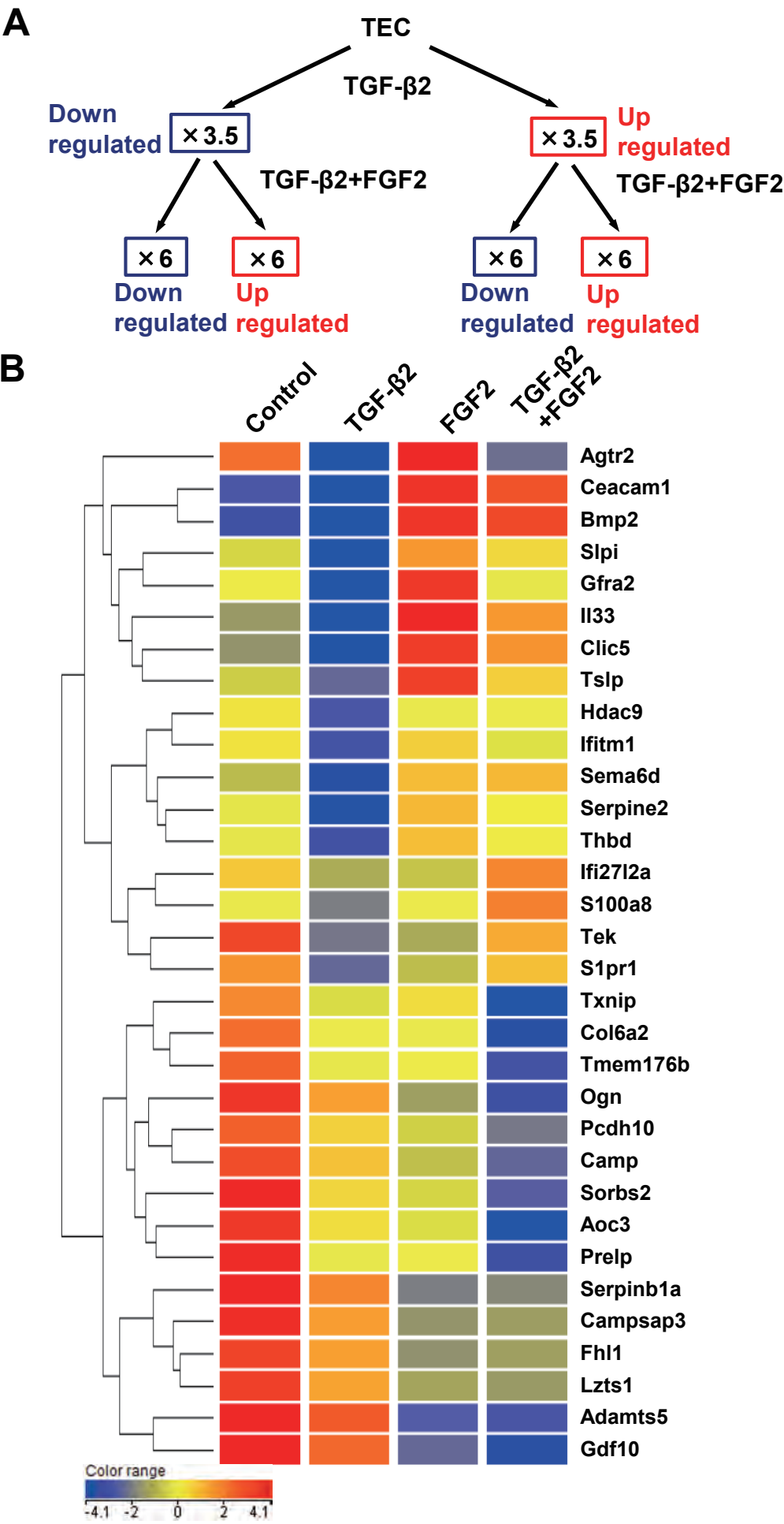

Fig. S7

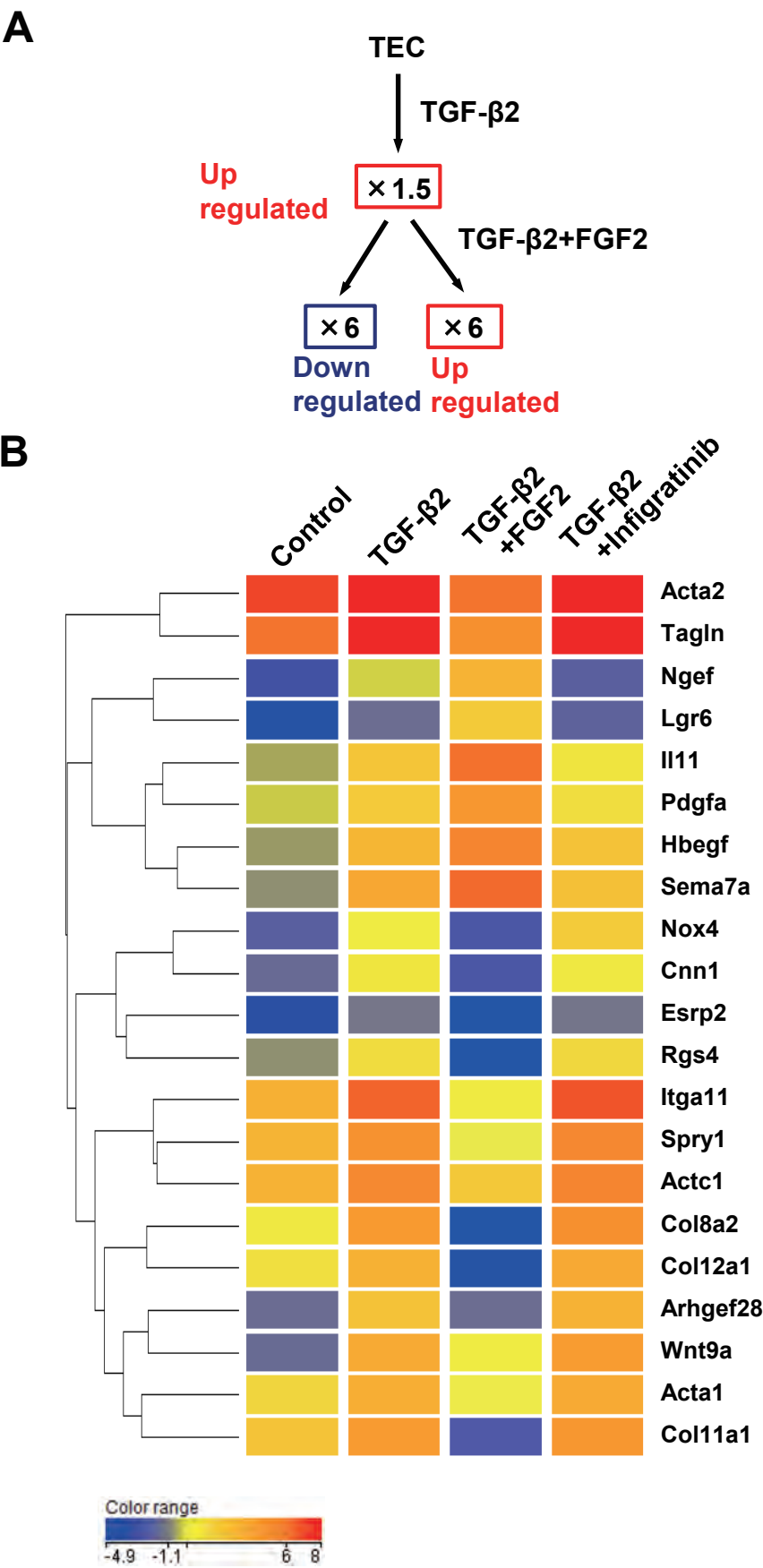

Fig. S8

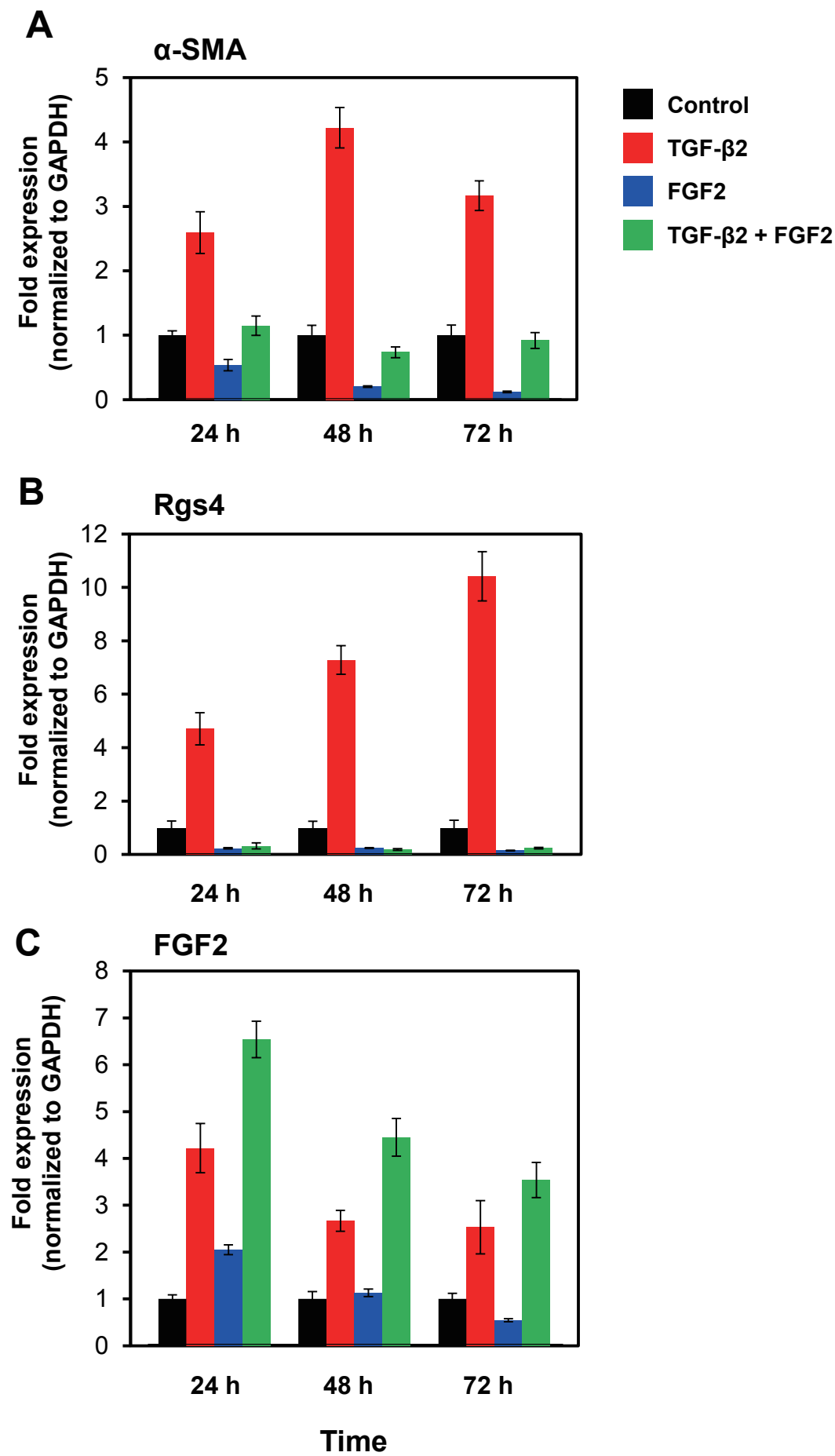

Fig. S9

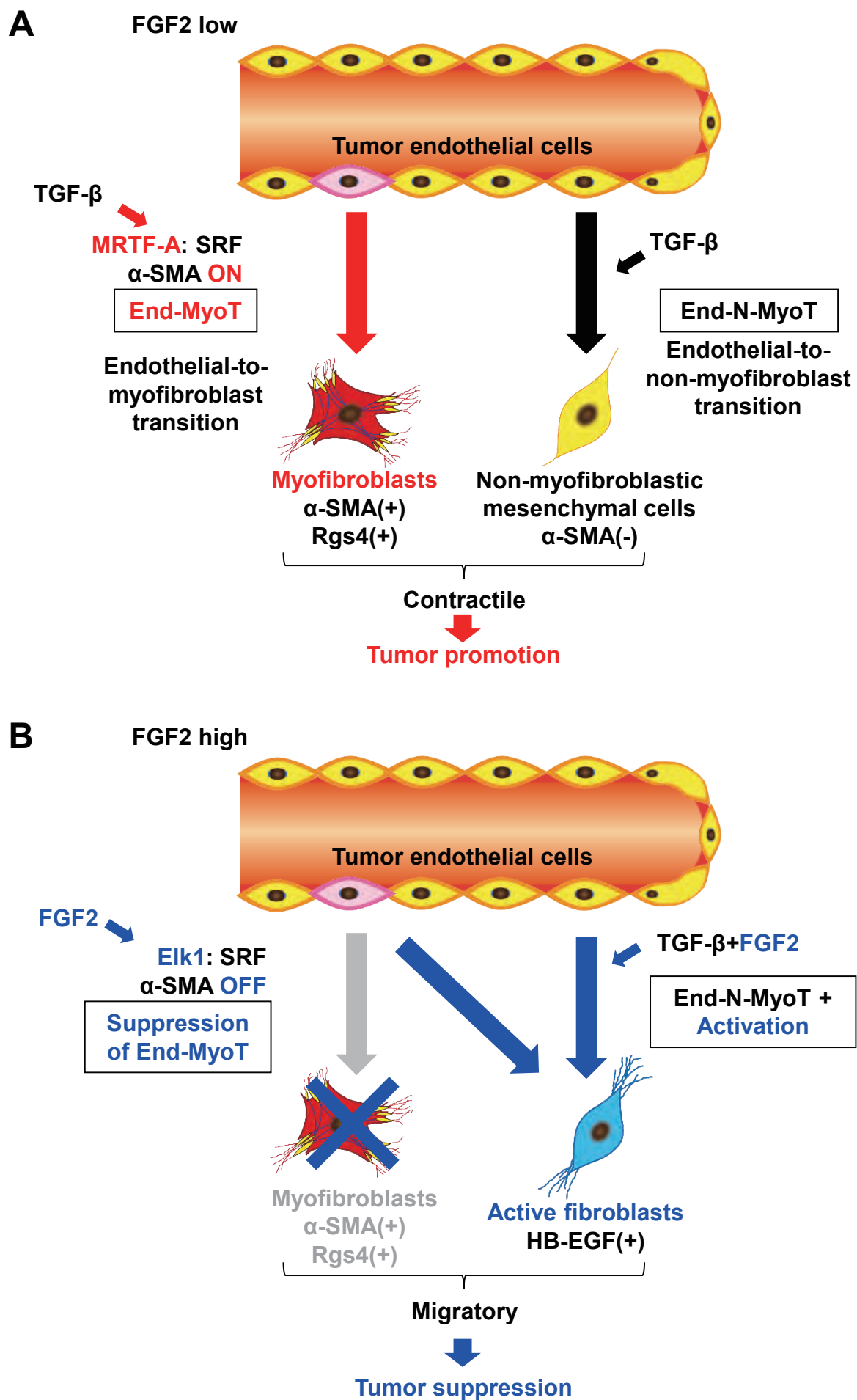

Fig. S10

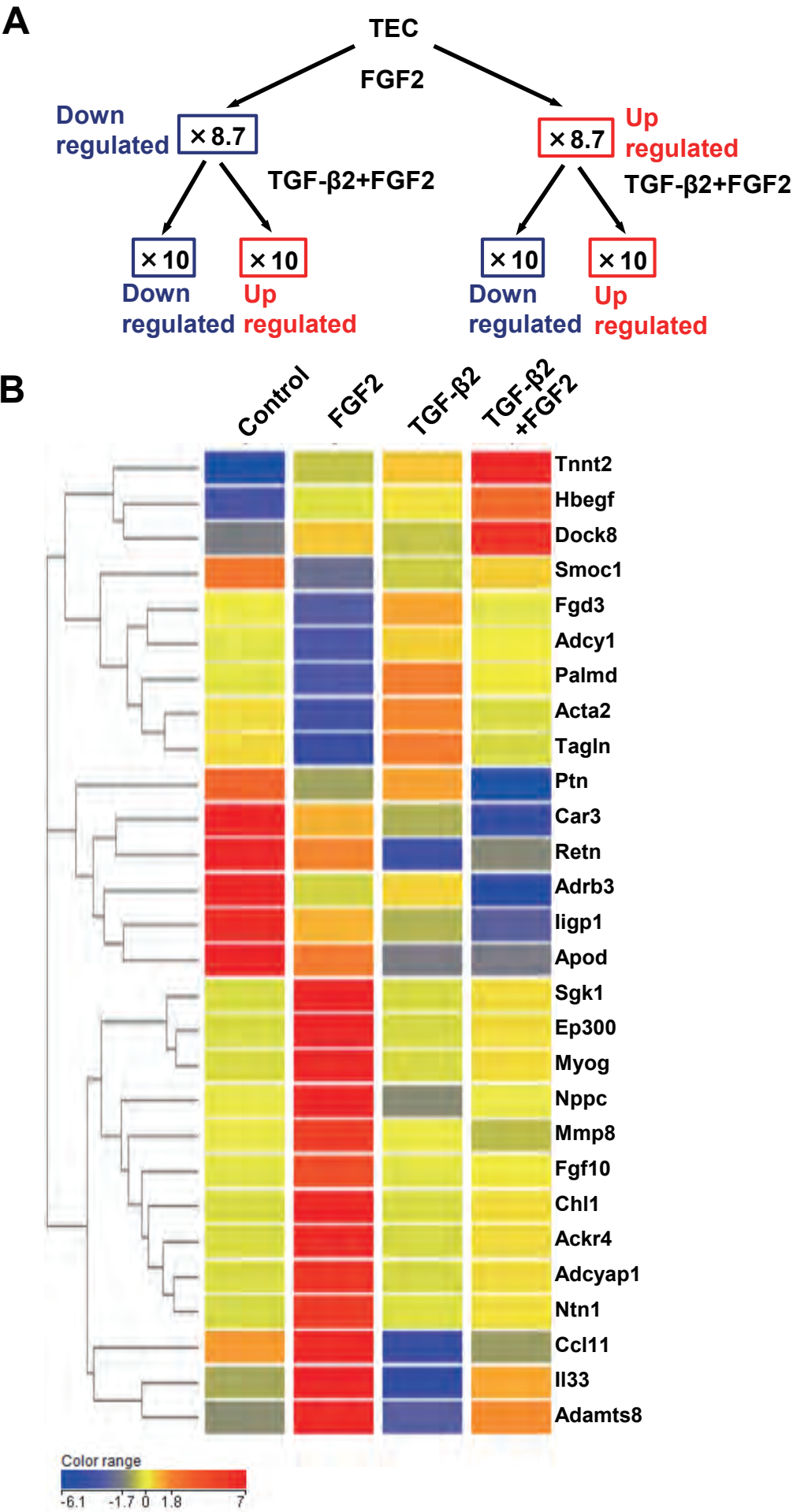

Fig. S11

A

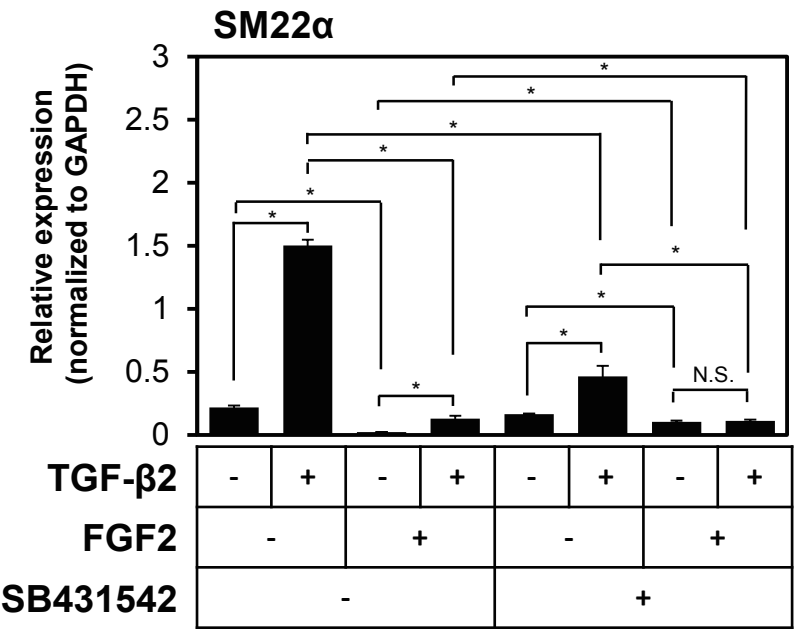

B

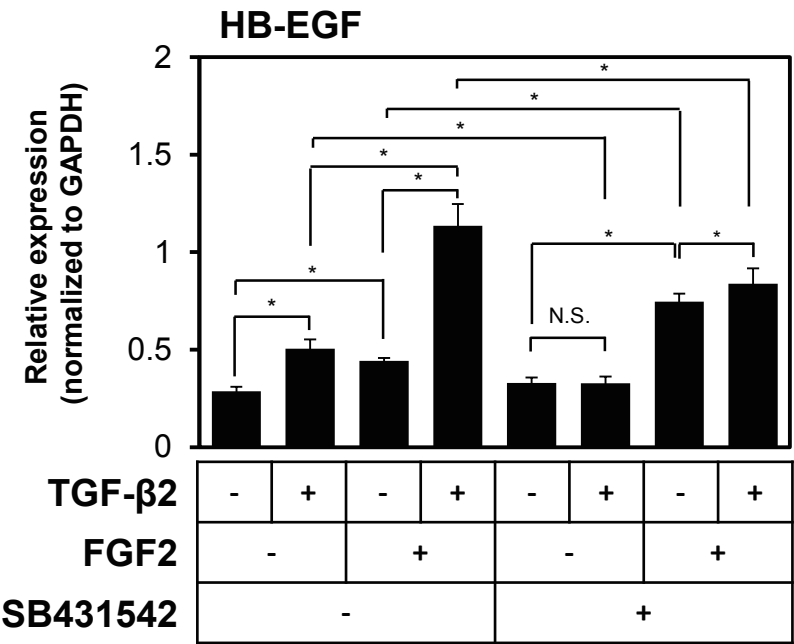

Supplement: Supplementary file 1 — Fig. S1. Two modes of TGF‐β‐induced mesenchymal transition of TECs. Fig. S2. Expression of various endothelial and mesenchymal markers in multiple types of endothelial and mesenchymal cells. Fig. S3. Effects of TGF‐β2, FGF2 and Infigratinib on the expression of endothelial and myofibroblast markers in TECs. Fig. S4. Effects of TGF‐β2 and FGF2 on the tube forming ability of TECs. Fig. S5. Effects of TGF‐β2 and VEGF‐A on the expression of mesenchymal markers in TECs. Fig. S6. Differential effects of FGF2 on the TGF‐β2‐mediated expression of various markers in TECs. Fig. S7. Differential effects of FGF2 and Infigratinib on the TGF‐β2‐mediated expression of various markers in TECs. Fig. S8. Effects of TGF‐β2 and FGF2 on the expression of mesenchymal markers and FGF2 in TECs. Fig. S9. Roles of TGF‐β2 and FGF2 signals in the regulation of End‐MyoT and End‐N‐MyoT of TECs. Fig. S10. Differential effects of TGF‐β2 on the FGF2‐mediated expression of various markers in TECs. Fig. S11. Effects of TGF‐β2, FGF2, and SB431542 on the expression of mesenchymal markers in TECs. [file MOL2-13-1706-s001.pdf]
