# Supplementary material for: Fibroblast growth factor signals regulate transforming growth factor‐β‐induced endothelial‐to‐myofibroblast transition of tumor endothelial cells via Elk1
Source: Mol Oncol. 2019 Jun 19;13(8):1706–24. doi: 10.1002/1878-0261.12504 (PMC6670013; doi:10.1002/1878-0261.12504)
Supplement: Supplementary file 2 — Table S1. Primers used for RT‐PCR. Table S2. List of genes whose expression is upregulated by TGF‐β2 and further modulated by FGF2 in combination with TGF‐β2. Table S3. List of genes whose expression is downregulated by TGF‐β2 and further modulated by FGF2 in combination with TGF‐β2. Table S4. List of genes whose expression is upregulated by TGF‐β2 and further modulated by Infigratinib in combination with TGF‐β2. Table S5. List of genes whose expression is downregulated by TGF‐β2 and further modulated by Infigratinib in combination with TGF‐β2. Table S6. List of genes whose expression is regulated by FGF2 and further modulated by TGF‐β2 in combination with FGF2. [file MOL2-13-1706-s002.pdf]

Supplemental Tables

Table S1. Primers used for RT-PCR

Transcript	F/R	Sequence (5' to 3')
Acta2 (α-SMA)	forward	AGCGTGAGATTGTCCGTGACAT
	reverse	GCGTTCGTTTCCAATGGTGA
Col1a1	forward	CTCCATGGCCTCTGCAACA
	reverse	GCATGTCCGATGTTTCCAGT
Elk1	forward	AGCGGCCAGAAGTTTGTCTA
	reverse	ATGGCCGAGGTTACAGACAC
Eng (Endoglin)	forward	TGCGTGAAGTCCACGTTCTCTT
	reverse	TTCGAAACGAGGACCAGGAA
Fgf2	forward	AGCGGCTCTACTGCAAGAAC
	reverse	GCCGTCCATCTTCCTTCATA
Fn1 (Fibronectin 1)	forward	GACAGGAGGAAATAGCCC
	reverse	CATCGTGCAAGGCAACCAC
Gapdh	forward	TGCAGTGGCAAAGTGGAGATT
	reverse	TGCCGTTGAATTTGCCGT
Hbegf	forward	CGGGGAGTGCAGATACCTG
	reverse	TTCTCCACTGGTAGAGTCAGC
Kdr (Vegfr2)	forward	GATGCAGGAAACTACACGGTCA
	reverse	TCCATAGGCGAGATCAAGGCT
Mkl1 (MRTFA)	forward	GCCTAAGCAGCAGGAAAATG
	reverse	GGTGGCTCTTTGAAATCTGC
Nrp1 (Neuropilin-1)	forward	GTGGGAAGATTGCACCTTCTC
	reverse	CCCCATGTGTCTCATAGTCAGAG
Rgs4	forward	GAGTGCAAAGGACATGAAACATC
	reverse	TTTTCCAACGATTCAGCCCAT
Tagln (SM22α)	forward	GTGTGGCTGAAGAATGGTGTGA
	reverse	GCCACCTGTTCCATCTGCTTAA
Tek (Tie2)	forward	GAAACATCCCTCACCTGCATTG
	reverse	TTTCGCCCATTCTCTGGTCA
Vegfa	forward	GCAGGCTGCTGTAACGATGAA
	reverse	ATGTGCTGGCTTTGGTGAGGT

Table S2. List of genes whose expression is upregulated by TGF-β2 and further modulated by FGF2 in combination with TGF-β2

Gene Symbol	[control] vs [TGF-β2] log FC	[control] vs [FGF2] log FC	[TGF-β2] vs [TGF-β2 +FGF2] log FC	Description
Acta1	2.14	-3.99	-3.15	actin, alpha 1, skeletal muscle (Acta1)
Acta2	1.81	-4.04	-2.70	actin, alpha 2, smooth muscle, aorta (Acta2)
Actc1	2.29	-3.80	-3.23	actin, alpha, cardiac muscle 1 (Actc1)
Ak5	1.89	-1.77	-2.96	adenylate kinase 5 (Ak5)
Arhgef28	3.86	-0.03	-3.73	Rho guanine nucleotide exchange factor (GEF) 28 (Arhgef28)
Avil	4.58	-1.56	-4.92	advillin (Avil)
Bcl6b	2.28	6.64	3.16	B cell CLL/lymphoma 6, member B (Bcl6b)
C1qtnf3	6.60	-4.02	-10.8	C1q and tumor necrosis factor related protein 3 (C1qtnf3)
C1qtnf3	6.97	-5.77	-11.63	C1q and tumor necrosis factor related protein 3 (C1qtnf3)
Cacna1g	2.11	-2.22	-6.99	calcium channel, voltage-dependent, T type, alpha 1G subunit (Cacna1g)
Capn6	1.80	-0.27	-5.45	calpain 6 (Capn6)
Car6	4.66	-0.63	-2.64	carbonic anhydrase 6 (Car6)
Ch25h	2.30	-1.23	-3.33	cholesterol 25-hydroxylase (Ch25h)
Cnn1	2.14	-3.20	-3.25	calponin 1 (Cnn1)
Col11a1	2.15	-4.62	-6.54	collagen, type XI, alpha 1 (Col11a1)
Col12a1	2.06	-4.80	-6.62	collagen, type XII, alpha 1 (Col12a1)
Col8a2	3.71	-6.66	-10.62	collagen, type VIII, alpha 2 (Col8a2)
Cox6a2	2.80	-0.66	-2.76	cytochrome c oxidase subunit VIa polypeptide 2 (Cox6a2)
Dner	3.12	0.03	-3.52	delta/notch-like EGF repeat containing (Dner)
Esrp2	3.28	-1.11	-3.91	epithelial splicing regulatory protein 2 (Esrp2)
Eya4	2.30	-1.24	-3.64	EYA transcriptional coactivator and phosphatase 4 (Eya4)
Fndc1	4.09	-1.15	-5.42	fibronectin type III domain containing 1 (Fndc1)
Gm16159	2.27	-1.72	-3.33	predicted gene 16159 (Gm16159), long non-coding RNA
Hbegf	3.88	3.13	2.59	heparin-binding EGF-like growth factor (Hbegf)
Hhipl1	2.40	0.90	-2.90	hedgehog interacting protein-like 1

Il11	3.01	4.63	4.01	interleukin 11 (Il11)
Itga11	3.26	-4.04	-5.95	integrin alpha 11 (Itga11)
Kcnn4	4.76	7.47	3.22	potassium intermediate/small conductance calcium-activated channel, subfamily N, member 4 (Kcnn4)
Kcnn4	4.87	7.68	3.32	potassium intermediate/small conductance calcium-activated channel, subfamily N, member 4 (Kcnn4)
Kif21b	2.59	-1.51	-2.79	kinesin family member 21B (Kif21b)
Lgr5	4.09	-2.47	-6.74	leucine rich repeat containing G protein coupled receptor 5 (Lgr5)
Lgr6	3.44	4.97	3.27	leucine-rich repeat-containing G protein-coupled receptor 6 (Lgr6)
Mamdc2	4.74	2.86	-3.2	MAM domain containing 2 (Mamdc2)
Mbp	2.30	-3.60	-5.14	myelin basic protein (Mbp)
Meox1	5.60	0.38	-2.96	mesenchyme homeobox 1 (Meox1)
Myo7a	3.26	-1.26	-3.31	myosin VIIA (Myo7a)
Ncam1	2.57	-1.07	-2.87	neural cell adhesion molecule 1 (Ncam1)
Ngef	2.08	6.74	3.62	neuronal guanine nucleotide exchange factor (Ngef)
Nox4	2.39	-1.33	-4.3	NADPH oxidase 4 (Nox4), transcript variant 1
Nox4	2.48	-1.59	-3.3	NADPH oxidase 4 (Nox4), transcript variant 2
Ostn	2.51	-3.64	-6.33	osteocrin (Ostn)
Pdgfa	2.33	2.58	2.62	platelet derived growth factor, alpha (Pdgfa)
Pkp2	1.89	-2.31	-2.66	plakophilin 2 (Pkp2)
Ptpru	4.54	-2.34	-6.16	protein tyrosine phosphatase, receptor type, U (Ptpru)
Rasl11b	2.09	-2.77	-5.07	RAS-like, family 11, member B (Rasl11b)
Rgs4	2.21	-4.23	-7.56	regulator of G-protein signaling 4 (Rgs4)
Sema7a	4.63	7.49	2.88	sema domain, immunoglobulin domain (Ig), and GPI membrane anchor, (semaphorin) 7A (Sema7a)
Spry1	1.83	-1.56	-4.82	sprouty homolog 1 (Drosophila) (Spry1)
Tagln	1.99	-4.43	-3.01	transgelin (Tagln)
Tnnt2	5.67	3.93	4.42	troponin T2, cardiac (Tnnt2)
Wisp1	2.89	-1.45	-3.39	WNT1 inducible signaling pathway protein 1 (Wisp1)
Wnt9a	4.99	-0.33	-3.01	wingless-type MMTV integration site family, member 9A (Wnt9a)
Wt1	4.39	-2.68	-6.52	Wilms tumor 1 homolog (Wt1)

Table S3. List of genes whose expression is downregulated by TGF-β2 and further modulated by FGF2 in combination with TGF-β2

Gene Symbol	[control] vs [TGF-β2] log FC	[control] vs [FGF2] log FC	[TGF-β2] vs [TGF-β2 +FGF2] log FC	Description
Abca9	-2.09	-3.86	-3.99	ATP-binding cassette, sub-family A (ABC1), member 9 (Abca9)
Abcb11	-1.98	-0.15	4.46	ATP-binding cassette, sub-family B (MDR/TAP), member 11 (Abcb11)
Adamts2	-2.27	-2.65	-3.13	a disintegrin-like and metallopeptidase (reprolysin type) with thrombospondin type 1 motif, 2 (Adamts2)
Adamts5	-1.91	-7.34	-5.61	a disintegrin-like and metallopeptidase (reprolysin type) with thrombospondin type 1 motif, 5 (aggrecanase-2) (Adamts5)
Adamtsl1	-1.97	-4.88	-3.34	ADAMTS-like 1 (Adamtsl1)
Adgrd1	-1.86	-6.74	-3.19	adhesion G protein-coupled receptor D1
Adgrl3	-1.81	1.27	3.26	adhesion G protein-coupled receptor L3
Adm	-2.95	-4.44	-4.58	adrenomedullin (Adm)
Adrb3	-1.85	-4.81	-3.14	adrenergic receptor, beta 3 (Adrb3)
Adrb3	-4.44	-5.62	-4.82	adrenergic receptor, beta 3 (Adrb3)
Aff3	-1.85	-4.94	-3.27	AF4/FMR2 family, member 3 (Aff3)
Aff3	-2.14	-6.86	-4.80	AF4/FMR2 family, member 3 (Aff3)
Agtr2	-8.19	2.00	3.55	angiotensin II receptor, type 2 (Agtr2)
Als2cr12	-4.70	-3.53	3.62	amyotrophic lateral sclerosis 2 (juvenile) chromosome region, candidate 12 (human) (Als2cr12)
Amy1	-2.82	-1.13	-3.54	amylase 1, salivary (Amy1), transcript variant 1
Amy1	-2.56	-5.69	-4.60	amylase 1, salivary (Amy1), transcript variant 1
Aoc3	-3.16	-3.82	-4.61	amine oxidase, copper containing 3
Aqp3	-4.38	-0.36	3.28	aquaporin 3 (Aqp3)
Arhgap6	-3.05	2.80	4.91	Rho GTPase activating protein 6 (Arhgap6)
Aspa	-3.63	-1.57	2.60	aspartoacylase (Aspa)
Bmp2	-2.68	6.65	8.90	bone morphogenetic protein 2 (Bmp2)
C1ra	-3.68	-2.95	-3.01	complement component 1, r subcomponent A (C1ra)
C1rb	-3.80	-3.28	-2.94	complement component 1, r subcomponent B (C1rb)
C3	-7.94	-5.86	-2.64	complement component 3 (C3)
Calca	-2.93	-1.80	5.74	calcitonin-related polypeptide alpha
Camp	-2.23	-3.83	-3.28	cathelicidin antimicrobial peptide (Camp)

Camsap3	-2.37	-5.40	-2.85	calmodulin regulated spectrin-associated protein family, member 3 (Camsap3)
Car3	-10.19	-7.54	-2.64	carbonic anhydrase 3
Cbfa2t3	-2.68	-0.20	4.52	core-binding factor, runt domain, alpha subunit 2, translocated to, 3 (human) (Cbfa2t3)
Ccl8	-2.35	2.71	4.12	chemokine (C-C motif) ligand 8 (Ccl8)
Cd55	-4.85	-1.07	3.99	CD55 molecule, decay accelerating factor for complement (Cd55)
Cd55	-4.22	-1.19	3.51	CD55 molecule, decay accelerating factor for complement
Cd55	-4.11	-0.95	3.35	CD55 molecule, decay accelerating factor for complement
Cd55	-3.94	-1.35	3.43	CD55 molecule, decay accelerating factor for complement
Cd55b	-4.45	-1.08	3.71	CD55 molecule, decay accelerating factor for complement B (Cd55b)
Cdh13	-2.11	3.80	5.61	cadherin 13 (Cdh13)
Cdrt4	-2.10	-0.63	5.85	CMT1A duplicated region transcript 4 (Cdrt4)
Ceacam1	-2.59	6.50	8.32	carcinoembryonic antigen-related cell adhesion molecule 1 (Ceacam1)
Chrm1	-3.05	1.24	3.33	cholinergic receptor, muscarinic 1, CNS (Chrm1)
Clic5	-2.45	5.00	5.69	chloride intracellular channel 5 (Clic5)
Col28a1	-6.94	-3.69	2.91	collagen, type XXVIII, alpha 1 (Col28a1)
Col6a1	-1.95	-1.66	-3.39	collagen, type VI, alpha 1 (Col6a1)
Col6a2	-2.33	-2.38	-3.70	collagen, type VI, alpha 2
Cpb1	-5.09	3.55	4.03	carboxypeptidase B1 (tissue) (Cpb1)
Cxcl12	-5.97	-5.91	-2.61	chemokine (C-X-C motif) ligand 12 (Cxcl12)
Cyp7b1	-4.16	-5.26	-4.41	cytochrome P450, family 7, subfamily b, polypeptide 1 (Cyp7b1)
Cyp7b1	-4.21	-5.27	-4.19	cytochrome P450, family 7, subfamily b, polypeptide 1 (Cyp7b1)
Disp2	-4.19	3.37	7.08	dispatched RND tramsporter family member 2 (Disp2)
Entpd2	-2.43	-4.19	-2.89	ectonucleoside triphosphate diphosphohydrolase 2 (Entpd2)
Fhl1	-1.86	-4.91	-2.75	four and a half LIM domains 1 (Fhl1)
Flrt3	-2.66	1.82	3.36	fibronectin leucine rich transmembrane protein 3 (Flrt3)
Gas6	-2.49	-4.81	-3.86	growth arrest specific 6 (Gas6)
Gdf10	-3.41	-8.30	-6.07	growth differentiation factor 10 (Gdf10)
Gfra2	-5.29	3.38	5.08	glial cell line derived neurotrophic factor family receptor alpha 2 (Gfra2)
Gprc5b	-2.01	0.56	2.69	G protein-coupled receptor, class C, group 5, member B
Gria4	-2.47	-2.39	-3.43	glutamate receptor, ionotropic, AMPA4 (alpha 4) (Gria4)
H19	-5.33	-5.68	-3.58	H19, imprinted maternally expressed transcript (H19)
H2-M1	-4.48	0.28	6.83	histocompatibility 2, M region locus 1 (H2-M1)
Hbb-y	-5.35	-4.19	5.85	hemoglobin Y, beta-like embryonic chain (Hbb-y)
Hcar2	-3.52	-3.15	4.77	hydroxycarboxylic acid receptor 2 (Hcar2)

Hdac9	-3.13	-0.28	2.89	histone deacetylase 9 (Hdac9)
Hhip	-2.19	2.75	5.97	Hedgehog-interacting protein (Hhip)
Ifi27l2a	-1.85	-1.40	3.01	interferon, alpha-inducible protein 27 like 2A (Ifi27l2a)
Ifitm1	-3.25	0.28	2.69	interferon induced transmembrane protein 1 (Ifitm1)
Igf1	-1.82	-6.79	-7.59	insulin-like growth factor 1 (Igf1)
Il33	-2.60	7.34	5.60	interleukin 33 (Il33)
Kcnk3	-5.86	0.03	4.24	potassium channel, subfamily K, member 3 (Kcnk3)
Kitl	-2.72	1.77	2.65	KIT ligand
Kitl	-2.98	1.49	2.68	KIT ligand
Klb	-5.54	-4.39	2.61	klotho beta (Klb)
Krt90	-4.02	-2.87	2.61	keratin 90 (Krt90)
Lama4	-2.55	-3.28	-3.09	laminin, alpha 4 (Lama4)
Lama4	-2.61	-3.77	-3.30	laminin, alpha 4 (Lama4)
Lamb3	-4.32	-2.48	5.69	laminin, beta 3 (Lamb3)
Lamb3	-5.66	-2.52	7.06	laminin, beta 3 (Lamb3)
Lancl3	-2.42	2.78	4.90	LanC lantibiotic synthetase component C-like 3 (bacterial) (Lancl3)
Lbp	-5.83	-4.11	-2.79	lipopolysaccharide binding protein (Lbp)
Lgi4	-2.47	1.39	3.77	leucine-rich repeat LGI family, member 4 (Lgi4)
Lpl	-3.11	-3.96	-4.07	lipoprotein lipase
Lpl	-3.06	-3.03	-4.57	lipoprotein lipase
Lpl	-2.87	-3.85	-4.08	lipoprotein lipase
Lpl	-2.67	-3.24	-3.77	lipoprotein lipase
Lpl	-3.35	-3.20	-4.34	lipoprotein lipase
Lurap1l	-1.83	-6.52	-4.63	leucine rich adaptor protein 1-like (Lurap1l)
Lyz1	-2.55	-3.53	-2.65	lysozyme 1 (Lyz1)
Lyz2	-2.77	-7.21	-4.61	lysozyme 2 (Lyz2)
Lzts1	-2.03	-4.63	-2.79	leucine zipper, putative tumor suppressor 1
Maf	-3.75	-5.70	-3.57	avian musculoaponeurotic fibrosarcoma oncogene homolog (Maf)
Mib2	-3.06	1.46	3.71	mindbomb E3 ubiquitin protein ligase 2
Ms4a4b	-2.11	-3.01	-3.05	membrane-spanning 4-domains, subfamily A, member 4B
Ms4a4d	-2.63	-2.97	-5.25	membrane-spanning 4-domains, subfamily A, member 4D (Ms4a4d)
Nefl	-4.10	5.16	5.78	neurofilament, light polypeptide
Nefl	-2.15	7.31	6.58	neurofilament, light polypeptide
Nefm	-3.19	3.36	3.11	neurofilament, medium polypeptide (Nefm)

Nkain2	-2.10	-0.09	2.70	Na+/K+ transporting ATPase interacting 2
Nnat	-1.89	-0.30	4.20	neuronatin
Nov	-2.66	-2.87	-4.48	nephroblastoma overexpressed gene (Nov)
Ogn	-2.15	-4.94	-4.57	osteoglycin (Ogn)
Olfr63	-3.03	1.37	3.75	olfactory receptor 63 (Olfr63)
Omd	-3.59	-5.25	-5.68	osteomodulin (Omd)
Pcdh10	-2.08	-3.14	-2.67	protocadherin 10 (Pcdh10)
Pcdh7	-2.51	2.31	4.48	protocadherin 7
Plekha6	-3.34	2.26	3.56	pleckstrin homology domain containing, family A member 6
Plpp3	-4.31	-0.25	3.42	phospholipid phosphatase 3 (Plpp3)
Ppp1r3c	-5.47	-5.32	-2.96	protein phosphatase 1, regulatory (inhibitor) subunit 3C (Ppp1r3c)
Prelp	-4.04	-3.88	-3.07	proline arginine-rich end leucine-rich repeat (Prelp)
Prelp	-3.74	-3.79	-2.69	proline arginine-rich end leucine-rich repeat (Prelp)
Prl2c1	-3.82	4.56	6.42	Prolactin family 2, subfamily c, member 1 (Prl2c1)
Prl2c3	-2.85	5.60	6.47	prolactin family 2, subfamily c, member 3 (Prl2c3)
Prl2c5	-4.16	5.00	7.19	prolactin family 2, subfamily c, member 5 (Prl2c5)
Rgs16	-2.37	0.49	3.98	regulator of G-protein signaling 16 (Rgs16)
Rnf144a	-1.91	-3.24	-4.81	ring finger protein 144A (Rnf144a)
Rspo2	-3.85	-7.40	-3.72	R-spondin 2
S100a8	-2.01	0.08	4.05	S100 calcium binding protein A8
S1pr1	-4.10	-2.47	3.29	sphingosine-1-phosphate receptor 1 (S1pr1)
Scara5	-5.39	-2.22	-3.71	scavenger receptor class A, member 5
Sdr42e1	-2.75	-5.00	-2.73	short chain dehydrogenase/reductase family 42E, member 1
Sema6d	-2.76	1.76	4.59	sema domain, transmembrane domain (TM), and cytoplasmic domain, (semaphorin) 6D (Sema6d)
Serpina3g	-3.29	-2.93	-2.87	serine (or cysteine) peptidase inhibitor, clade A, member 3G (Serpina3g)
Serpinb1a	-2.48	-6.40	-3.72	serine (or cysteine) peptidase inhibitor, clade B, member 1a (Serpinb1a)
Serpine2	-3.74	1.11	4.07	serine (or cysteine) peptidase inhibitor, clade E, member 2
Slc14a1	-2.26	4.93	5.30	solute carrier family 14 (urea transporter), member 1 (Slc14a1)
Slpi	-5.29	1.97	6.15	secretory leukocyte peptidase inhibitor (Slpi)
Smpd3	-2.14	-6.76	-6.40	sphingomyelin phosphodiesterase 3, neutral (Smpd3)
Sorbs2	-4.54	-5.43	-3.09	sorbin and SH3 domain containing 2 (Sorbs2)
Sox9	-2.39	0.70	2.94	SRY (sex determining region Y)-box 9 (Sox9)
Sox9	-2.04	1.09	2.92	SRY (sex determining region Y)-box 9 (Sox9)
Sult1a1	-5.97	-3.88	2.82	sulfotransferase family 1A, phenol-preferring, member 1 (Sult1a1)

Syn2	-2.63	-4.24	-2.91	synapsin II (Syn2), transcript variant Iia
Tek	-5.38	-4.37	3.37	endothelial-specific receptor tyrosine kinase (Tek)
Thbd	-2.90	0.99	3.18	thrombomodulin (Thbd)
Tmem176b	-2.66	-2.49	-2.91	transmembrane protein 176B (Tmem176b)
Tmem179	-2.68	0.83	4.76	transmembrane protein 179 (Tmem179)
Tmod1	-2.38	2.47	4.61	tropomodulin 1 (Tmod1)
Tmod1	-2.79	2.29	4.78	tropomodulin 1 (Tmod1)
Trib1	-1.95	0.37	2.87	tribbles pseudokinase 1
Tslp	-1.88	3.90	3.03	thymic stromal lymphopoietin
Tspan11	-3.78	2.79	5.97	thioredoxin interacting protein (Txnip)
Txnip	-2.15	-1.46	-3.99	thioredoxin interacting protein
Unc5a	-2.15	2.79	3.93	unc-5 netrin receptor A (Unc5a)
Xaf1	-2.37	-1.55	2.78	XIAP associated factor 1 (Xaf1)

Table S4. List of genes whose expression is upregulated by TGF-β2 and further modulated by Infigratinib in combination with TGF-β2

Gene Symbol	[control] vs [TGF-β2] log FC	[TGF-β2]vs [TGF-β2 +FGF2] log FC	[TGF-β2] vs [TGFβ2 +Infigratinib] log FC	Description
Aass	3.4	-3.08	-3.53	aminoadipate-semialdehyde synthase (Aass)
Abca6	0.83	-3.31	0.11	ATP-binding cassette, sub-family A (ABC1), member 6 (Abca6)
Abcb1a	0.67	3.8	-1.29	ATP-binding cassette, sub-family B (MDR/TAP), member 1A (Abcb1a)
Abcb7	2.75	-3.35	-2.17	ATP-binding cassette, sub-family B (MDR/TAP), member 7 (Abcb7)
Acta1	2	-3	0.18	actin, alpha 1, skeletal muscle (Acta1)
Acta2	1.7	-3.32	0.48	actin, alpha 2, smooth muscle, aorta (Acta2)
Actc1	2.18	-3.27	0.25	actin, alpha, cardiac muscle 1 (Actc1)
Adamts16	2.88	-3.23	-1.25	a disintegrin-like and metallopeptidase (reprolysin type) with thrombospondin type 1 motif, 16 (Adamts16)
Adamtsl3	2.76	-3.11	-0.85	ADAMTS-like 3 (Adamtsl3)
Add2	6.55	4.87	-5.28	adducin 2 (beta) (Add2)
Adgrb2	2.73	3.67	-1.59	adhesion G protein-coupled receptor B2 (Adgrb2)
Adgrf3	4.52	-3.38	-2.78	adhesion G protein-coupled receptor F3 (Adgrf3)
Adgrg5	0.99	3.51	0.56	adhesion G protein-coupled receptor G5 (Adgrg5)
Adgrv1	2.87	-3.21	-3.1	adhesion G protein-coupled receptor V1
Adssl1	1.11	3.43	0.95	adenylosuccinate synthetase like 1 (Adssl1)
Agbl4	0.95	3.4	-0.98	ATP/GTP binding protein-like 4 (Agbl4)
Ak5	2.54	-3.7	-0.06	adenylate kinase 5 (Ak5)
Akr1c14	1	-6	-3.64	aldo-keto reductase family 1, member C14 (Akr1c14)
Akr1cl	2.17	-2.82	-2.61	aldo-keto reductase family 1, member C-like
Alcam	1.84	3.23	-0.43	activated leukocyte cell adhesion molecule (Alcam)
Ambn	5.25	-5.59	-5.48	ameloblastin (Ambn)
Ank2	2.51	-2.84	-1.5	ankyrin 2, brain
Ankrd35	1.21	-4.3	0.01	ankyrin repeat domain 35 (Ankrd35)
Arhgap22	0.93	4.01	-1.29	Rho GTPase activating protein 22 (Arhgap22)

Arhgap45	2.13	3.33	-0.26	Rho GTPase activating protein 45 (Arhgap45)
Arhgef28	3.91	-3.88	0.82	Rho guanine nucleotide exchange factor (GEF) 28 (Arhgef28)
Arhgef4	2.91	-3.23	-1.57	Rho guanine nucleotide exchange factor (GEF) 4
Atg9b	1.18	3.5	-1.39	autophagy related 9B (Atg9b)
Atp1a3	1.37	4	-0.49	ATPase, Na+/K+ transporting, alpha 3 polypeptide (Atp1a3)
Atp6v0d2	2.71	4.25	-1.97	ATPase, H+ transporting, lysosomal V0 subunit D2 (Atp6v0d2)
Avil	4.42	-5.08	0.22	advillin (Avil)
Awat2	4.78	-6.43	-3.51	acyl-CoA wax alcohol acyltransferase 2 (Awat2)
B3galnt2	4.92	-5.07	-5.16	UDP-GalNAc:betaGlcNAc beta 1,3-galactosaminyltransferase, polypeptide 2
B4galnt2	4.3	-6.03	0.64	beta-1,4-N-acetyl-galactosaminyl transferase 2 (B4galnt2)
Baiap2l1	0.68	2.93	0.35	BAI1-associated protein 2-like 1 (Baiap2l1)
Banf2	2.34	-3.37	-2.45	barrier to autointegration factor 2 (Banf2)
Bcl6b	2.5	3.26	-1.63	B cell CLL/lymphoma 6, member B (Bcl6b)
Bdh1	1.05	-2.67	0.34	3-hydroxybutyrate dehydrogenase, type 1 (Bdh1)
Bdkrb2	5.24	3.45	-2.85	bradykinin receptor, beta 2
Becn2	3.63	-3.97	-3.84	beclin 2 (Becn2)
Bnc1	2.25	3.94	1.6	basonuclin 1 (Bnc1)
C1qtnf3	6.94	-12.18	0.04	C1q and tumor necrosis factor related protein 3 (C1qtnf3)
Cacna1g	2	-5.47	-0.37	calcium channel, voltage-dependent, T type, alpha 1G subunit (Cacna1g)
Cadps	3.27	-3.61	-3.48	Ca2+-dependent secretion activator (Cadps)
Cald1	2.38	-2.71	0.08	caldesmon 1
Capn6	1.79	-4.9	0.19	calpain 6 (Capn6)
Car6	5.01	-2.65	0	carbonic anhydrase 6 (Car6)
Carmil1	3.64	-3.97	-3.86	leucine rich repeat containing 16A (Lrrc16a)
Casp14	2.98	-2.85	-1.8	caspase 14 (Casp14)
Ccdc113	2.25	-3.19	-3.18	coiled-coil domain containing 113 (Ccdc113),
Ccdc116	2.46	-2.66	-0.44	coiled-coil domain containing 116 (Ccdc116)
Ccdc155	0.76	-2.61	-0.38	coiled-coil domain containing 155 (Ccdc155)
Ccdc77	3.6	-3.26	-3.66	coiled-coil domain containing 77 (Ccdc77)
Ccr6	3.22	-3.47	-3.43	chemokine (C-C motif) receptor 6 (Ccr6)
Cd33	1.55	3.28	-1.76	CD33 antigen (Cd33)
Cd53	3.16	3.01	-4.56	CD53 antigen (Cd53)
Cd68	1.29	2.63	-0.23	CD68 antigen (Cd68)
Cd74	1.51	-5.18	-0.38	CD74 antigen (invariant polypeptide of major histocompatibility complex, class II

				antigen-associated) (Cd74)
Cdh3	5.58	-6.62	-0.54	cadherin 3 (Cdh3)
Cdh6	4.53	3.32	-2.3	cadherin 6 (Cdh6)
Cdk8	2.65	-3.02	-2.92	cyclin-dependent kinase 8
Ces1g	3.57	-3.92	-3.79	carboxylesterase 1G (Ces1g)
Cfap46	4.85	-5.19	-5.08	cilia and flagella associated protein 46 (Cfap46)
Ch25h	2.17	-3.22	-2.49	cholesterol 25-hydroxylase (Ch25h)
Chrna1	1.11	8.56	-1.36	cholinergic receptor, nicotinic, alpha polypeptide 1 (muscle) (Chrna1)
Cilp	4.98	-7.22	-1.23	cartilage intermediate layer protein, nucleotide pyrophosphohydrolase (Cilp)
Clec4b1	1.94	-2.89	-2.76	C-type lectin domain family 4, member b1 (Clec4b1)
Clec5a	2.59	-2.92	-1.54	C-type lectin domain family 5, member a (Clec5a)
Cnga1	2.53	-2.9	-2.8	cyclic nucleotide gated channel alpha 1
Cnn1	2.43	-3.26	-0.11	calponin 1 (Cnn1)
Cobl	2.32	-3.02	0.44	cordon-bleu WH2 repeat (Cobl)
Col11a1	2.44	-4.71	-0.21	collagen, type XI, alpha 1 (Col11a1)
Col12a1	2.19	-7.75	0.39	collagen, type XII, alpha 1 (Col12a1)
Col1a1	0.89	-3.58	-0.46	collagen, type I, alpha 1 (Col1a1)
Col5a2	1.66	-4.21	-0.59	collagen, type V, alpha 2 (Col5a2)
Col6a4	3.81	-4.9	-4.08	collagen, type VI, alpha 4 (Col6a4)
Col8a2	3.72	-11.04	0.54	collagen, type VIII, alpha 2 (Col8a2)
Cox6a2	2.81	-2.88	0.05	cytochrome c oxidase subunit VIa polypeptide 2 (Cox6a2)
Cpvl	5.51	-5.84	-5.73	carboxypeptidase, vitellogenic-like (Cpvl)
Crabp1	0.86	4.52	-3.18	cellular retinoic acid binding protein I (Crabp1)
Crispld2	1.85	-4.01	1.51	cysteine-rich secretory protein LCCL domain containing 2 (Crispld2)
Csn3	1.5	6.27	-1.58	casein kappa
Cyp11a1	0.96	2.65	-1.16	cytochrome P450 side chain cleavage enzyme 11a1
Cyth2	4.34	-3.74	-4.73	cytohesin 2
Dact1	3.42	-6.59	-2.56	dishevelled-binding antagonist of beta-catenin 1 (Dact1)
Dapk2	2.41	-2.77	-2.65	death-associated protein kinase 2 (Dapk2)
Dennd1c	0.81	3.08	-0.85	den domain containing 1C (Dennd1c)
Dlg2	2.33	-2.66	0.75	discs, large homolog 2 (Drosophila) (Dlg2)
Dner	3.07	-3.76	1.01	delta/notch-like EGF repeat containing (Dner)
Dock8	0.78	5.09	-1.45	dedicator of cytokinesis 8 (Dock8)
Dppa2	0.69	2.61	-0.94	developmental pluripotency associated 2 (Dppa2)

Drc1	1.64	-3.35	0.69	dynein regulatory complex subunit 1 (Drc1)
Drd3	4	-2.85	-4.84	dopamine receptor D3 (Drd3)
Dusp5	0.95	2.69	-2.36	dual specificity phosphatase 5 (Dusp5)
Dusp5	1.4	2.71	-2.12	dual specificity phosphatase 5
Dyrk3	1.65	2.81	-0.14	dual-specificity tyrosine-(Y)-phosphorylation regulated kinase 3 (Dyrk3)
E2f8	1.06	2.87	-1.63	E2F transcription factor 8 (E2f8)
Edn1	1.67	-7.35	0.78	endothelin 1 (Edn1)
Ehf	3.19	-4.26	-4.15	ets homologous factor (Ehf)
Eif2ak4	1.26	2.97	0.91	GB
Eln	2.9	-3.39	-1.99	elastin (Eln)
Erc2	0.66	4	1.4	ELKS/RAB6-interacting/CAST family member 2 (Erc2)
Esrp2	2.62	-4.88	-0.03	epithelial splicing regulatory protein 2 (Esrp2)
Etl4	5.37	-5.72	-5.6	enhancer trap locus 4
Etv1	0.65	4.7	-1.13	ets variant 1 (Etv1)
Etv4	5.24	2.69	-3.86	ets variant 4 (Etv4)
Exoc6b	4.01	-3.25	-3.92	exocyst complex component 6B
Eya4	2.5	-2.85	-0.79	EYA transcriptional coactivator and phosphatase 4 (Eya4)
F2rl1	1.39	3.47	-1.38	coagulation factor II (thrombin) receptor-like 1 (F2rl1), mRNA [NM_007974]
F2rl2	0.75	2.6	-0.94	coagulation factor II (thrombin) receptor-like 2 (F2rl2)
F2rl3	1.02	3.53	-1.25	coagulation factor II (thrombin) receptor-like 3 (F2rl3)
Fam110c	0.65	2.83	-1.24	family with sequence similarity 110, member C (Fam110c)
Fam167b	4.97	-5.31	-5.17	family with sequence similarity 167, member B (Fam167b)
Fbxl20	5.19	-4.8	-5.42	f-box and leucine rich repeat protein 20 (Fbxl20)
Ffar1	2.41	-2.76	-2.63	free fatty acid receptor 1 (Ffar1)
Fgf16	5.78	-6.5	-6.37	fibroblast growth factor 16 (Fgf16)
Fmo3	1.13	2.96	0.93	flavin containing monooxygenase 3 (Fmo3)
Fmod	1.68	-3.67	-1.25	fibromodulin (Fmod)
Fndc1	3.07	-5.9	0.6	fibronectin type III domain containing 1 (Fndc1)
Focad	5.16	-5.49	-5.39	focadhesin
Foxr1	1.54	-2.6	-1.25	forkhead box R1 (Foxr1)
Frem2	5.66	-5.25	-5.9	Fras1 related extracellular matrix protein 2 (Frem2)
Frmd4b	3.41	-3.73	-3.63	FERM domain containing 4B (Frmd4b)
Fzd6	3.27	-2.71	0.2	frizzled class receptor 6 (Fzd6)
Gabrg1	4.31	-4.67	-4.55	gamma-aminobutyric acid (GABA) A receptor, subunit gamma 1 (Gabrg1)

Gadd45b	1.38	-2.77	-0.43	growth arrest and DNA-damage-inducible 45 beta (Gadd45b)
Gcfc2	0.65	2.68	-0.87	GC-rich sequence DNA binding factor 2
Gimap3	4.58	-4.93	-3.71	GTPase, IMAP family member 3 (Gimap3)
Gja4	0.86	2.95	0.14	gap junction protein, alpha 4 (Gja4)
Gjb2	4.43	-4.76	-3.28	gap junction protein, beta 2 (Gjb2)
Gli2	1.74	-3.71	-0.29	GLI-Kruppel family member GLI2 (Gli2)
Glrp1	3.7	2.96	-1.13	glutamine repeat protein 1 (Glrp1)
Glt8d2	4.95	-5.27	-5.15	glycosyltransferase 8 domain containing 2 (Glt8d2)
Gmeb1	2.18	-4.29	-4.17	glucocorticoid modulatory element binding protein 1
Gpr21	5.84	-6.17	-6.05	G protein-coupled receptor 21 (Gpr21)
Gpr6	2.77	-3.14	-3.02	G protein-coupled receptor 6
Gpr84	3.06	-2.8	-3.28	G protein-coupled receptor 84 (Gpr84)
Gprc5a	1.75	2.61	-0.47	G protein-coupled receptor, family C, group 5, member A (Gprc5a)
Gsto2	0.95	3.13	-1.14	glutathione S-transferase omega 2 (Gsto2)
Gsto2	1.49	3.47	-0.04	glutathione S-transferase omega 2 (Gsto2)
Hal	5.93	-5.77	-5.6	histidine ammonia lyase
Has1	1.05	2.81	-2.24	hyaluronan synthase1 (Has1)
Hbegf	3.7	2.62	-0.63	heparin-binding EGF-like growth factor (Hbegf)
Hephl1	1.2	-3.51	-3.39	hephaestin-like 1 (Hephl1)
Hhipl1	2.57	-2.8	0.31	hedgehog interacting protein-like 1
Hist1h3a	2.11	-3.78	-2.52	histone cluster 1, H3a (Hist1h3a)
Hmga1	0.83	4.88	-0.76	high mobility group AT-hook 1 (Hmga1)
Hoxb9	4.02	-2.94	-2.32	homeobox B9 (Hoxb9), mRNA [NM_008270]
Hs6st2	0.84	-4.43	0.53	heparan sulfate 6-O-sulfotransferase 2 (Hs6st2)
Hs6st2	1.2	-5.38	0.14	heparan sulfate 6-O-sulfotransferase 2 (Hs6st2)
Hsd3b6	6.32	-6.67	-6.56	hydroxy-delta-5-steroid dehydrogenase, 3 beta- and steroid delta-isomerase 6 (Hsd3b6)
Htr3b	3.43	-3.8	-3.7	5-hydroxytryptamine (serotonin) receptor 3B (Htr3b)
Ice1	5.2	-5.53	-3.67	interactor of little elongation complex ELL subunit 1
Igfbp5	0.67	-2.62	-2.49	insulin-like growth factor binding protein 5 (Igfbp5)
Il11	2.81	4.04	-1.42	interleukin 11 (Il11)
Il1rl1	1.03	4.83	-1.81	interleukin 1 receptor-like 1 (Il1rl1)
Itga11	3.25	-5.89	0.32	integrin alpha 11 (Itga11)
Itga11	3.06	-5.29	0.21	integrin alpha 11
Itga2	1.52	3.85	-0.03	integrin alpha 2 (Itga2)

Itgax	1.96	5.5	-2.15	integrin alpha X (Itgax)
Itgb3	1.07	2.84	-1.39	integrin beta 3 (Itgb3)
Itgb3	1.07	2.88	-0.95	integrin beta 3 (Itgb3)
Jph2	2.68	-3	0.93	junctophilin 2 (Jph2)
Kcnb1	3.26	-5.62	-5.5	potassium voltage gated channel, Shab-related subfamily, member 1 (Kcnb1)
Kcng1	4.77	-5.21	-5.08	potassium voltage-gated channel, subfamily G, member 1 (Kcng1)
Kcnh1	3.52	3.01	-0.42	potassium voltage-gated channel, subfamily H (eag-related), member 1 (Kcnh1)
Kcnip4	3.34	-2.92	-1.65	Kv channel interacting protein 4 (Kcnip4)
Kcnj5	2.41	-2.75	-2.64	potassium inwardly-rectifying channel, subfamily J, member 5 (Kcnj5)
Kcnma1	5.68	-6.04	-5.92	potassium large conductance calcium-activated channel, subfamily M, alpha member 1 (Kcnma1)
Kcnn4	4.52	3.12	-2.23	potassium intermediate/small conductance calcium-activated channel, subfamily N, member 4 (Kcnn4)
Kcnn4	5.11	3.29	-2.26	potassium intermediate/small conductance calcium-activated channel, subfamily N, member 4 (Kcnn4)
Kcnn4	4.81	3.33	-2.29	potassium intermediate/small conductance calcium-activated channel, subfamily N, member 4 (Kcnn4)
Kdm2a	3.78	-3.22	-3.14	lysine (K)-specific demethylase 2A
Kif21b	2.45	-2.72	-0.06	kinesin family member 21B (Kif21b)
Kif26b	1.43	-2.81	-0.06	kinesin family member 26B (Kif26b)
Kif26b	1.2	-2.86	0.04	kinesin family member 26B (Kif26b)
Klkb1	2.94	-3.17	-2.7	kallikrein B, plasma 1 (Klkb1)
Krt24	3.43	-3.76	-3.65	keratin 24 (Krt24)
Krt75	3.76	-5.86	-5.21	keratin 75 (Krt75)
Lama1	8.11	-8.47	1.24	laminin, alpha 1 (Lama1)
Lama2	1.99	-3.67	1.07	laminin, alpha 2
Lancl2	1.81	-2.79	-2.74	LanC (bacterial lantibiotic synthetase component C)-like 2 (Lancl2)
Lce1f	5.65	2.78	-5.87	late cornified envelope 1F (Lce1f)
Lce1m	2.22	3.86	-2.46	late cornified envelope 1M (Lce1m)
Lgals7	1.91	3.14	-1.33	lectin, galactose binding, soluble 7 (Lgals7)
Lgr5	4.11	-7.2	-1.76	leucine rich repeat containing G protein coupled receptor 5 (Lgr5)
Lgr6	2.8	3.2	-0.41	leucine-rich repeat-containing G protein-coupled receptor 6 (Lgr6)
Lgr6	2.8	3.52	-0.39	leucine-rich repeat-containing G protein-coupled receptor 6
Lmod2	3.27	-3.6	-3.48	leiomodin 2 (cardiac) (Lmod2)
Lrrc6	5.26	-5.33	-5.5	leucine rich repeat containing 6 (testis) (Lrrc6)

Lypd1	1.87	-3.18	-3.09	Ly6/Plaur domain containing 1 (Lypd1)
Mag	2.53	-2.63	-2.53	myelin-associated glycoprotein
Mageb4	3.62	-3.95	-3.84	melanoma antigen, family B, 4 (Mageb4)
Magi2	1.99	-3.44	-3.65	membrane associated guanylate kinase, WW and PDZ domain containing 2 (Magi2)
Mamdc2	4.93	-3.18	0.31	MAM domain containing 2 (Mamdc2)
Map3k19	2.87	-3.2	0.77	mitogen-activated protein kinase kinase kinase 19 (Map3k19)
Mapk6	5.84	-3.56	-6.07	mitogen-activated protein kinase 6
Matn3	4.38	-4.44	0.4	matrilin 3 (Matn3)
Mbp	2.16	-5.07	2.01	myelin basic protein (Mbp)
Mefv	3.82	-3.58	-3.62	Mediterranean fever (Mefv)
Meox1	5.47	-3.09	-2.7	mesenchyme homeobox 1 (Meox1)
Mid1	2	-3.63	-1.56	midline 1 (Mid1)
Mmp1b	4.02	-4.35	-3.6	matrix metallopeptidase 1b (interstitial collagenase) (Mmp1b)
Mpp3	0.72	-2.99	-0.49	membrane protein, palmitoylated 3 (MAGUK p55 subfamily member 3) (Mpp3)
Mrgprh	5.66	-5.87	-4.84	MAS-related GPR, member H (Mrgprh)
Mro	4.31	-4.67	-4.54	maestro (Mro)
Mro	5.05	-4.17	-5.67	maestro (Mro)
Msh5	0.85	3.17	0.39	mutS homolog 5
Mtmr7	5.07	-3.87	-5.13	myotubularin related protein 7
Mtus1	2.27	-2.65	0.85	mitochondrial tumor suppressor 1 (Mtus1)
Mtus1	3.05	-2.92	-0.13	mitochondrial tumor suppressor 1
Mup19	3.25	-3.99	-3.35	major urinary protein 19 (Mup19)
Myh7	3.04	-3.37	-3.26	myosin, heavy polypeptide 7, cardiac muscle, beta
Myl9	0.84	-5.36	0.91	myosin, light polypeptide 9, regulatory (Myl9)
Mylk3	4.76	-6.04	-6.06	myosin light chain kinase 3 (Mylk3)
Mylk4	3.11	-3.44	-3.33	myosin light chain kinase family, member 4 (Mylk4)
Myo3b	1.09	-3.41	-0.71	myosin IIIB (Myo3b)
Myo7a	3.47	-3.27	0.01	myosin VIIA (Myo7a)
Nap1l5	1.64	-3.36	-2.28	nucleosome assembly protein 1-like 5 (Nap1l5)
Nav2	0.94	2.83	-1.14	neuron navigator 2
Ncam1	2.46	-2.68	-0.43	neural cell adhesion molecule 1 (Ncam1)
Ncdn	2.67	-2.8	-2.24	neurochondrin (Ncdn)
Ndufa4l2	1.05	-3.68	-0.43	NADH dehydrogenase (ubiquinone) 1 alpha subcomplex, 4-like 2 (Ndufa4l2)
Necab1	4.33	-4.68	-4.56	N-terminal EF-hand calcium binding protein 1 (Necab1)

Ngef	2.66	3.29	-1.88	neuronal guanine nucleotide exchange factor (Ngef)
Nme6	5.64	-4.82	-4.66	NME/NM23 nucleoside diphosphate kinase 6
Nmu	1.85	3.62	-2.11	neuromedin U (Nmu)
Nmur1	2.93	-3.49	-3.74	neuromedin U receptor 1 (Nmur1)
Nox4	2.67	-3.06	1.25	NADPH oxidase 4 (Nox4), transcript variant 2
Nrg1	2.45	-2.62	-0.15	neuregulin 1
Nrg2	4.74	-5.08	-4.97	neuregulin 2
Nrip2	0.87	-4.34	0.82	nuclear receptor interacting protein 2 (Nrip2)
Nt5c1b	4.02	-4.35	-4.25	5'-nucleotidase, cytosolic IB
Ntng2	2.56	-2.77	-0.87	netrin G2 (Ntng2)
Oasl1	1.41	2.88	0.25	2'-5' oligoadenylate synthetase-like 1 (Oasl1)
Oasl1	1.21	2.93	0.36	2'-5' oligoadenylate synthetase-like 1 (Oasl1)
Olfr1106	2.25	-2.62	-2.49	olfactory receptor 1106 (Olfr1106)
Olfr1221	5.68	-6	-5.39	olfactory receptor 1221 (Olfr1221)
Olfr1271	4.82	-3.59	-5.03	olfactory receptor 1271
Olfr1275	5.62	-5.97	-5.85	olfactory receptor 1275 (Olfr1275)
Olfr1314	5.33	-5.68	-5.54	olfactory receptor 1314 (Olfr1314)
Olfr1362	3.59	-3.32	-3.83	olfactory receptor Olfr1362
Olfr1535	3.04	-3.36	-3.25	olfactory receptor 1535 (Olfr1535)
Olfr181	3.51	-3.83	-3.71	olfactory receptor 181 (Olfr181)
Olfr351	3.88	-4.24	-4.11	olfactory receptor 351 (Olfr351)
Olfr355	4.15	-3.6	-4.38	olfactory receptor 355 (Olfr355)
Olfr370	0.67	3.76	-0.88	olfactory receptor 370 (Olfr370)
Olfr52	4.58	-5.6	-5.49	olfactory receptor 52 (Olfr52)
Olfr586	2.67	-3	-1.88	olfactory receptor 586 (Olfr586)
Olfr610	4.54	-4.88	-4.75	olfactory receptor 610 (Olfr610)
Olfr891	4.32	-5.58	-5.47	olfactory receptor 891 (Olfr891)
Oscp1	2.36	-2.72	-2.34	organic solute carrier partner 1
Ostn	2.73	-6.91	1.03	osteocrin (Ostn)
Palm3	2.68	-3.01	-0.42	paralemmin 3
Palmd	2.54	-2.69	0.59	palmdelphin
Pam	4.39	-4.72	-4.61	peptidylglycine alpha-amidating monooxygenase
Pcgf3	2.58	-2.65	-2.59	polycomb group ring finger 3
Pdgfa	2.2	2.62	-0.89	platelet derived growth factor, alpha (Pdgfa)

Pdzd7	1.81	-4.24	1.61	PDZ domain containing 7 (Pdzd7)
Phykpl	1.39	-2.63	-2.5	5-phosphohydroxy-L-lysine phospholyase
Pid1	1.39	-3.67	0.84	phosphotyrosine interaction domain containing 1
Pid1	1	-3.11	0.6	phosphotyrosine interaction domain containing 1
Pik3c2g	4.96	-6.21	-5.25	phosphatidylinositol 3-kinase, C2 domain containing, gamma polypeptide (Pik3c2g)
Pir	3.44	-4.13	-3.92	pirin
Pitx2	1.31	-2.77	-0.99	paired-like homeodomain transcription factor 2
Pkp2	1.91	-2.7	0.55	plakophilin 2 (Pkp2)
Pkp2	1.87	-3.18	-0.08	plakophilin 2
Pkp2	0.86	-2.85	1.39	plakophilin 2
Pla2g7	1.21	3.73	-0.24	phospholipase A2, group VII (platelet-activating factor acetylhydrolase, plasma) (Pla2g7)
Pla2g7	1.49	3.4	-0.65	phospholipase A2, group VII (platelet-activating factor acetylhydrolase, plasma) (Pla2g7)
Pla2g7	1.43	3.46	-0.54	phospholipase A2, group VII (platelet-activating factor acetylhydrolase, plasma) (Pla2g7)
Plk5	2.1	-2.98	-2.86	polo-like kinase 5 (Plk5)
Plxdc1	0.93	3.14	-1.33	plexin domain containing 1 (Plxdc1)
Plxdc2	2.9	-2.68	-1.73	plexin domain containing 2 (Plxdc2)
Pomt1	5.12	-4.43	-5.13	protein O-mannosyltransferase 1
Ppef1	0.68	-4.46	1.13	protein phosphatase with EF hand calcium-binding domain 1 (Ppef1)
Ppip5k1	2.77	-3.06	-3.44	diphosphoinositol pentakisphosphate kinase 1
Ppp1r12b	2.85	-3.22	-2.92	protein phosphatase 1, regulatory (inhibitor) subunit 12B (Ppp1r12b)
Ppp1r14a	1.62	-3.56	1.82	protein phosphatase 1, regulatory (inhibitor) subunit 14A (Ppp1r14a)
Prodh	2.45	-2.8	-2.68	proline dehydrogenase
Ptpn18	0.97	-2.87	1.03	protein tyrosine phosphatase, non-receptor type 18 (Ptpn18)
Ptpru	4.72	-6.73	0.01	protein tyrosine phosphatase, receptor type, U (Ptpru)
Rab39b	1.41	-3.76	-0.55	RAB39B, member RAS oncogene family (Rab39b)
Ralgapa2	4.38	-4.71	-2.91	Ral GTPase activating protein, alpha subunit 2 (catalytic)
Ramp1	0.64	6.18	-1.03	receptor (calcitonin) activity modifying protein 1 (Ramp1)
Rasl11b	2.01	-4.94	0.33	RAS-like, family 11, member B (Rasl11b)
Rbfox1	3.38	-3.71	-2.08	RNA binding protein, fox-1 homolog (C. elegans) 1 (Rbfox1)
Rcor3	5.62	-5.98	-5.86	REST corepressor 3 (Rcor3)
Rgs17	0.85	3.85	0.76	regulator of G-protein signaling 17 (Rgs17)
Rgs4	1.93	-8.02	0.16	regulator of G-protein signaling 4 (Rgs4)
Rhox13	3.16	-3.51	-3.38	reproductive homeobox 13 (Rhox13)
Rita1	5.02	-5.34	-5.23	RBPJ interacting and tubulin associated 1

Rps6ka3	2.38	-2.74	-2.61	ribosomal protein S6 kinase polypeptide 3
Rxfp2	3.2	-3.53	-3.41	relaxin/insulin-like family peptide receptor 2 (Rxfp2)
Selp	0.82	4.95	-2.03	selectin, platelet (Selp)
Sema7a	4.54	2.7	-1.26	sema domain, immunoglobulin domain (Ig), and GPI membrane anchor, (semaphorin) 7A (Sema7a)
Serbp1	3.18	-2.91	-3.43	serpine1 mRNA binding protein 1
Sgcg	0.96	-2.91	-0.26	sarcoglycan, gamma (dystrophin-associated glycoprotein) (Sgcg)
Sh3tc2	1.44	-4.15	1.13	SH3 domain and tetratricopeptide repeats 2 (Sh3tc2)
Sla	2.27	-2.61	0.25	src-like adaptor (Sla), transcript variant 1
Slamf9	4.47	-2.83	-0.78	SLAM family member 9 (Slamf9)
Slc20a1	2.47	2.77	-1.14	solute carrier family 20, member 1 (Slc20a1)
Slc23a1	3.85	-3.59	-2.88	solute carrier family 23 (nucleobase transporters), member 1 (Slc23a1)
Slc25a42	1.31	-2.81	-2.74	solute carrier family 25, member 42
Slc27a6	2.74	-4.29	1.39	solute carrier family 27 (fatty acid transporter), member 6 (Slc27a6)
Slc28a3	1.62	3.66	-1.85	solute carrier family 28 (sodium-coupled nucleoside transporter), member 3
Slc4a10	2.85	-3.2	-2.94	solute carrier family 4, sodium bicarbonate cotransporter-like, member 10 (Slc4a10)
Slc51a	5.13	-4.62	-4.93	solute carrier family 51, alpha subunit
Slc7a13	2.96	-2.92	-2.36	solute carrier family 7, (cationic amino acid transporter, y+ system) member 13 (Slc7a13)
Slit2	1.18	-2.65	-1.96	slit homolog 2 (Drosophila) (Slit2)
Sltm	4.55	-4.89	-4.76	SAFB-like, transcription modulator
Snapc1	4.17	-5.44	-2.96	small nuclear RNA activating complex, polypeptide 1
Sp9	2.75	-3.06	-2.94	trans-acting transcription factor 9 (Sp9)
Spata19	3.52	-3.07	-3.73	spermatogenesis associated 19 (Spata19)
Spdye4b	2.63	-2.94	-2.83	speedy/RINGO cell cycle regulator family, member E4B (Spdye4b)
Speer3	1.65	-3.86	-3.11	spermatogenesis associated glutamate (E)-rich protein 3 (Speer3)
Spire1	3.15	-3.52	-3.39	spire homolog 1 (Drosophila)
Sppl2c	2.25	-2.66	-0.84	signal peptide peptidase 2C (Sppl2c)
Spry1	1.8	-4.78	0.55	sprouty homolog 1 (Drosophila) (Spry1)
Ssu72	4.44	-5.05	-4.23	Ssu72 RNA polymerase II CTD phosphatase homolog (yeast)
Stbd1	3.12	-2.75	-0.8	starch binding domain 1
Stpg3	5.33	-5.66	-1.85	sperm tail PG rich repeat containing 3 (Stpg3)
Styk1	2.35	-3.01	-0.15	serine/threonine/tyrosine kinase 1 (Styk1)
Tagln	1.91	-3.21	0.36	transgelin (Tagln)
Tarm1	2.29	3.69	-1.14	T cell-interacting, activating receptor on myeloid cells 1 (Tarm1)
Tbpl2	4.36	-4.7	-4.57	TATA box binding protein like 2 (Tbpl2)

Tbx4	4.78	-5.11	-3.19	T-box 4 (Tbx4)
Tcp10b	1.66	-2.63	-3.72	t-complex protein 10b (Tcp10b)
Tdrd9	2.4	4.51	-2.61	tudor domain containing 9 (Tdrd9)
Tec	0.66	3.55	-0.62	tec protein tyrosine kinase (Tec)
Tenm2	3.12	-3.77	-3.65	teneurin transmembrane protein 2 (Tenm2)
Tex11	5.1	-6.05	-5.93	testis expressed gene 11 (Tex11)
Tfpi2	2.34	3.43	-2.99	tissue factor pathway inhibitor 2 (Tfpi2)
Tfr2	2.19	-2.6	0.29	transferrin receptor 2 (Tfr2)
Tg	3.36	-4.74	0.59	thyroglobulin (Tg)
Them7	3.07	-3.09	0.19	thioesterase superfamily member 7 (Them7)
Tiam1	2.82	-2.8	-1.2	T cell lymphoma invasion and metastasis 1 (Tiam1)
Tln2	1.31	-2.64	-0.74	talin 2 (Tln2)
Tm4sf4	1.23	3.7	-0.26	transmembrane 4 superfamily member 4 (Tm4sf4)
Tmem56	4.58	-4.93	-4.81	transmembrane protein 56
Tnfrsf13c	2.84	-3.38	-0.53	tumor necrosis factor receptor superfamily, member 13c (Tnfrsf13c)
Tnnt2	6.09	4.16	-3.73	troponin T2, cardiac (Tnnt2)
Tpk1	4.03	-4.91	-4.99	thiamine pyrophosphokinase
Trappc8	3.57	-3.92	-3.78	trafficking protein particle complex 8
Trim30b	5.44	-5.79	-5.67	tripartite motif-containing 30B (Trim30b)
Trim56	2.28	-2.62	-2.51	tripartite motif-containing 56 (Trim56)
Triml2	3.01	-3.35	-3.24	tripartite motif family-like 2 (Triml2)
Tro	2.87	-2.96	-3.08	trophinin (Tro), transcript variant 6
Trp63	1.91	-3.12	-3.01	transformation related protein 63
Trpm1	5.56	-5.89	-5.77	transient receptor potential cation channel, subfamily M, member 1 (Trpm1)
Trpm3	4.1	-4.45	-4.33	transient receptor potential cation channel, subfamily M, member 3
Ttc6	2.56	-2.9	-2.58	tetratricopeptide repeat domain 6 (Ttc6)
Ttc9	3.12	2.68	-1.97	tetratricopeptide repeat domain 9 (Ttc9)
Ttc9	1.72	2.92	-1.92	tetratricopeptide repeat domain 9 (Ttc9)
Txnrd2	3.15	-3.16	-3.36	thioredoxin reductase 2
Ubr4	2.3	-2.65	-0.65	ubiquitin protein ligase E3 component n-recognin 4
Unc13c	1.29	2.94	-1.49	unc-13 homolog C (C. elegans) (Unc13c)
Upp1	1.7	5.88	-1.63	uridine phosphorylase 1 (Upp1)
Vgf	0.76	-3.94	1.17	VGF nerve growth factor inducible (Vgf)
Vmn1r36	2.63	-2.99	-2.88	vomeronasal 1 receptor 36 (Vmn1r36)

Vmn1r4	2.44	-2.63	-2.65	vomeronasal 1 receptor 4 (Vmn1r4)
Vmn1r78	2.91	-3.23	-3.12	vomeronasal 1 receptor 78 (Vmn1r78)
Vmn2r7	1.77	-2.75	0.08	vomeronasal 2, receptor 7
Vps37a	3.99	-4.32	-4.2	vacuolar protein sorting 37A
Vstm2b	4.03	-4.39	-4.26	V-set and transmembrane domain containing 2B
Wdr63	1.02	3.51	-2.62	WD repeat domain 63 (Wdr63)
Wdsub1	2.81	-3.17	-2.78	WD repeat, SAM and U-box domain containing 1
Wdsub1	2.48	-3	-2.57	WD repeat, SAM and U-box domain containing 1 (Wdsub1)
Wisp1	2.72	-3.19	0.06	WNT1 inducible signaling pathway protein 1 (Wisp1)
Wisp1	3	-3.54	-0.09	WNT1 inducible signaling pathway protein 1 (Wisp1)
Wnt10a	2.67	-2.98	-2.91	wingless-type MMTV integration site family, member 10A (Wnt10a)
Wnt7b	0.71	4.6	-0.18	wingless-type MMTV integration site family, member 7B (Wnt7b)
Wnt9a	5.18	-2.96	0.76	wingless-type MMTV integration site family, member 9A (Wnt9a)
Wt1	3.96	-5.35	-0.87	Wilms tumor 1 homolog (Wt1)
Zcchc12	3.77	-3.54	0.07	zinc finger, CCHC domain containing 12 (Zcchc12)
Zfp612	3.29	-3.66	-3.33	zinc finger protein 612 (Zfp612)
Zfp770	2.21	-2.77	-2.21	zinc finger protein 770
Znrf4	3.82	-3.25	-3.7	zinc and ring finger 4 (Znrf4)

Table S5. List of genes whose expression is downregulated by TGF-β2 and further modulated by Infigratinib in combination with TGF-β2

Gene Symbol	[control]vs [TGF-β2] log FC	[TGF-β2] vs [TGF-β2 +FGF2] log FC	[TGF-β2] vs [TGF-β2 +Infigratinib] log FC	Description
Abca9	-2.16	-4.81	0.83	ATP-binding cassette, sub-family A (ABC1), member 9 (Abca9)
Abcc3	-1.29	2.81	0.18	ATP-binding cassette, sub-family C (CFTR/MRP), member 3 (Abcc3)
Abl2	-1.28	3.02	0.84	v-abl Abelson murine leukemia viral oncogene 2 (arg, Abelson-related gene) (Abl2)
Adam23	-0.96	-3.04	-1.64	a disintegrin and metallopeptidase domain 23 (Adam23)
Adamts5	-1.98	-4.77	0.50	a disintegrin-like and metallopeptidase (reprolysin type) with thrombospondin type 1 motif, 5 (aggrecanase-2) (Adamts5)
Adamts9	-1.66	-4.35	1.07	a disintegrin-like and metallopeptidase (reprolysin type) with thrombospondin type 1 motif, 9 (Adamts9)
Adamtsl1	-2.00	-2.62	0.73	ADAMTS-like 1 (Adamtsl1)
Adgrd1	-1.87	-4.51	0.46	adhesion G protein-coupled receptor D1 (Adgrd1)
Adgrl3	-1.31	3.14	0.39	adhesion G protein-coupled receptor L3 (Adgrl3)
Adm	-2.99	-4.77	0.50	adrenomedullin (Adm)
Adrb3	-4.32	-5.41	1.06	adrenergic receptor, beta 3 (Adrb3)
Aff3	-0.68	-3.32	0.29	AF4/FMR2 family, member 3 (Aff3)
Agtr2	-9.33	4.57	-0.15	angiotensin II receptor, type 2 (Agtr2)
Aldh1a1	-1.17	-5.76	1.39	aldehyde dehydrogenase family 1, subfamily A1 (Aldh1a1)
Als2cr12	-3.58	2.59	-0.39	amyotrophic lateral sclerosis 2 (juvenile) chromosome region, candidate 12 (human) (Als2cr12)
Alx3	-2.24	-2.79	0.15	aristaless-like homeobox 3 (Alx3)
Amy1	-2.75	-3.04	1.22	amylase 1, salivary (Amy1)
Amy2a5	-2.68	-3.14	1.15	amylase 2a5 (Amy2a5)
Ang2	-0.91	3.29	0.90	angiogenin, ribonuclease A family, member 2 (Ang2)
Angptl2	-1.18	3.73	-0.13	angiopoietin-like 2
Ankrd37	-0.72	2.99	-2.48	ankyrin repeat domain 37 (Ankrd37)
Aoc3	-3.19	-4.67	0.60	amine oxidase, copper containing 3 (Aoc3)
Aqp3	-4.31	3.63	-0.16	aquaporin 3 (Aqp3)

Arhgap20	-1.18	-6.79	1.57	Rho GTPase activating protein 20
Arhgap6	-3.35	5.04	0.49	Rho GTPase activating protein 6 (Arhgap6)
Arid1b	-1.27	2.75	0.92	AT rich interactive domain 1B (SWI-like) (Arid1b)
Arsi	-0.68	-3.33	-0.12	arylsulfatase i (Arsi)
Aspn	-0.99	-9.54	0.77	asporin (Aspn)
Atoh8	-1.68	-3.93	-0.67	atonal bHLH transcription factor 8 (Atoh8)
Atp8b1	-3.52	2.65	1.37	ATPase, class I, type 8B, member 1 (Atp8b1)
Azi2	-1.81	5.15	-0.14	5-azacytidine induced gene 2
B3gnt5	-4.68	3.38	-0.16	UDP-GlcNAc:betaGal beta-1,3-N-acetylglucosaminyltransferase 5 (B3gnt5)
Bcas1	-0.75	2.84	-0.21	breast carcinoma amplified sequence 1 (Bcas1)
Bik	-2.04	5.29	-0.14	BCL2-interacting killer (Bik)
Bmp2	-1.59	8.24	1.46	bone morphogenetic protein 2 (Bmp2)
Bsn	-0.68	2.87	0.87	bassoon (Bsn)
C1ra	-3.55	-3.08	0.93	complement component 1, r subcomponent A (C1ra)
C1rb	-3.89	-2.77	0.84	complement component 1, r subcomponent B (C1rb)
C1s1	-6.24	-2.59	2.18	complement component 1, s subcomponent 1 (C1s1)
C1s2	-6.30	-2.65	2.15	complement component 1, s subcomponent 2 (C1s2)
C3	-8.12	-2.67	1.41	complement component 3 (C3)
Cables1	-1.90	-2.95	-0.02	CDK5 and Abl enzyme substrate 1 (Cables1)
Camp	-2.27	-2.65	-0.73	cathelicidin antimicrobial peptide (Camp)
Camsap3	-2.66	-3.09	0.35	calmodulin regulated spectrin-associated protein family, member 3 (Camsap3)
Car3	-10.14	-3.23	2.97	carbonic anhydrase 3 (Car3)
Cbfa2t3	-1.86	3.78	-0.16	core-binding factor, runt domain, alpha subunit 2, translocated to, 3 (human) (Cbfa2t3
Ccl8	-2.67	4.42	0.06	chemokine (C-C motif) ligand 8 (Ccl8)
Cd34	-3.98	3.06	-0.22	CD34 antigen (Cd34)
Cd55	-4.73	3.85	0.59	CD55 molecule, decay accelerating factor for complement (Cd55)
Cd55b	-3.63	2.85	0.12	CD55 molecule, decay accelerating factor for complement B (Cd55b)
Cd93	-1.13	2.69	1.84	CD93 antigen (Cd93)
Cd96	-1.03	5.34	0.87	CD96 antigen, partial
Cdh13	-1.51	5.53	1.51	cadherin 13 (Cdh13)
Cdkn1c	-0.79	-3.00	1.79	cyclin-dependent kinase inhibitor 1C (P57) (Cdkn1c)
Ceacam1	-0.96	6.49	-0.33	carcinoembryonic antigen-related cell adhesion molecule 1 (Ceacam1)
Cgnl1	-1.67	3.33	2.49	cingulin-like 1 (Cgnl1)
Chrm1	-2.36	3.70	0.27	cholinergic receptor, muscarinic 1, CNS (Chrm1)

Chrm5	-1.46	4.95	0.25	cholinergic receptor, muscarinic 5 (Chrm5)
Chst3	-1.01	3.05	-0.10	carbohydrate (chondroitin 6/keratan) sulfotransferase 3 (Chst3)
Clic5	-1.87	4.94	3.85	chloride intracellular channel 5 (Clic5)
Cnn1	-0.98	-3.00	-1.08	calponin 1 (Cnn1)
Col28a1	-1.34	-3.45	-4.27	collagen, type XXVIII, alpha 1 (Col28a1)
Col2a1	-1.13	-2.92	-0.10	collagen, type II, alpha 1 (Col2a1)
Col6a1	-1.86	-3.44	-0.05	collagen, type VI, alpha 1 (Col6a1)
Col6a2	-2.44	-3.50	-0.21	collagen, type VI, alpha 2 (Col6a2)
Cpb1	-5.09	4.10	1.74	carboxypeptidase B1 (tissue) (Cpb1)
Cspg5	-1.54	2.61	-0.50	chondroitin sulfate proteoglycan 5 (Cspg5)
Ctnnal1	-0.60	2.85	0.57	catenin (cadherin associated protein), alpha-like 1 (Ctnnal1)
Cyp7b1	-4.27	-4.08	1.55	cytochrome P450, family 7, subfamily b, polypeptide 1 (Cyp7b1)
Cys1	-0.67	-4.93	2.26	cystin 1 (Cys1)
Ddc	-0.81	-4.92	0.87	dopa decarboxylase (Ddc)
Disp2	-3.48	6.36	-0.14	dispatched RND tramsporter family member 2 (Disp2)
Dnhd1	-1.18	2.84	0.23	dynein heavy chain domain 1
E2f8	-4.50	3.50	-0.62	E2F transcription factor 8
Ear10	-1.03	2.99	0.16	eosinophil-associated, ribonuclease A family, member 10 (Ear10)
Ecm2	-1.17	-4.05	0.33	extracellular matrix protein 2, female organ and adipocyte specific (Ecm2)
Ecscr	-0.77	-3.82	0.03	endothelial cell surface expressed chemotaxis and apoptosis regulator (Ecscr)
Entpd2	-2.51	-3.00	0.06	ectonucleoside triphosphate diphosphohydrolase 2 (Entpd2)
Ephb2	-0.80	-3.10	-1.95	Eph receptor B2 (Ephb2)
Eps8	-0.99	2.70	0.70	epidermal growth factor receptor pathway substrate 8 (Eps8)
Eva1c	-1.00	2.69	0.77	eva-1 homolog C (C. elegans) (Eva1c)
F13a1	-1.14	5.96	-0.43	coagulation factor XIII, A1 subunit (F13a1)
F3	-1.47	-4.06	0.97	coagulation factor III (F3)
Fam26f	-0.76	-4.58	0.27	family with sequence similarity 26, member F (Fam26f)
Fam78b	-1.54	3.64	-0.37	family with sequence similarity 78, member B (Fam78b)
Fam84a	-1.61	4.82	1.78	family with sequence similarity 84, member A (Fam84a)
Fas	-0.99	-4.35	1.39	Fas (TNF receptor superfamily member 6) (Fas)
Fcrls	-0.64	5.29	-0.15	Fc receptor-like S, scavenger receptor (Fcrls)
Fgf11	-1.26	-2.73	0.71	PREDICTED: fibroblast growth factor 11 (Fgf11)
Flrt3	-2.44	2.68	0.48	fibronectin leucine rich transmembrane protein 3 (Flrt3)
Fyb	-0.59	3.34	0.23	FYN binding protein (Fyb)

Gabra3	-1.20	-4.42	1.44	gamma-aminobutyric acid (GABA) A receptor, subunit alpha 3 (Gabra3)
Gabra5	-2.09	2.76	-0.16	gamma-aminobutyric acid (GABA) A receptor, subunit alpha 5 (Gabra5)
Galnt16	-1.55	-5.35	-0.04	UDP-N-acetyl-alpha-D-galactosamine:polypeptide N-acetylgalactosaminyltransferase 16 (Galnt16)
Gas6	-2.45	-4.00	1.26	growth arrest specific 6 (Gas6)
Gdf10	-3.37	-6.05	0.41	growth differentiation factor 10 (Gdf10)
Gfra2	-6.80	6.37	3.83	glial cell line derived neurotrophic factor family receptor alpha 2 (Gfra2)
Ggt5	-2.97	-3.34	0.57	gamma-glutamyltransferase 5 (Ggt5)
Gprc5b	-1.99	2.68	0.47	G protein-coupled receptor, family C, group 5, member B (Gprc5b)
Gria4	-1.44	-2.78	1.14	glutamate receptor, ionotropic, AMPA4 (alpha 4) (Gria4)
H2-M1	-4.35	6.88	0.15	histocompatibility 2, M region locus 1 (H2-M1)
H2-T24	-2.66	3.51	-0.15	histocompatibility 2, T region locus 24 (H2-T24)
Hacd4	-1.01	-3.34	0.44	3-hydroxyacyl-CoA dehydratase 4 (Hacd4)
Hhip	-0.99	4.57	-0.15	Hedgehog-interacting protein (Hhip)
Igf1	-1.72	-7.20	-0.43	insulin-like growth factor 1 (Igf1)
Igf2	-2.89	5.60	0.83	insulin-like growth factor 2 (Igf2)
Igfbp3	-1.29	-2.87	1.25	insulin-like growth factor binding protein 3
Ikzf2	-1.35	-2.59	1.99	IKAROS family zinc finger 2 (Ikzf2)
Il33	-0.74	2.94	0.54	interleukin 33 (Il33)
Il34	-1.48	4.56	-0.45	interleukin 34 (Il34)
Itga6	-1.29	4.05	-0.08	integrin alpha 6 (Itga6)
Itgb8	-3.29	2.89	2.22	integrin beta 8
Itln1	-1.26	4.72	-0.25	intelectin 1 (galactofuranose binding) (Itln1)
Junos	-2.90	5.04	0.56	jun proto-oncogene, opposite strand (Junos)
Kcnk2	-0.59	3.96	-0.16	potassium channel, subfamily K, member 2 (Kcnk2)
Kcnk3	-5.77	4.40	-0.67	potassium channel, subfamily K, member 3 (Kcnk3)
Kitl	-2.79	2.66	0.69	kit ligand (Kitl)
Klra2	-1.18	2.61	0.12	killer cell lectin-like receptor, subfamily A, member 2 (Klra2)
Lama4	-2.65	-3.11	0.48	laminin, alpha 4 (Lama4)
Lamb3	-3.85	5.38	0.05	laminin, beta 3 (Lamb3)
Lancl3	-2.67	5.05	0.66	LanC lantibiotic synthetase component C-like 3 (bacterial) (Lancl3)
Lbp	-5.81	-3.86	1.22	lipopolysaccharide binding protein (Lbp)
Lce3a	-1.03	2.79	0.19	late cornified envelope 3A (Lce3a)
Lfng	-0.71	-3.29	-1.77	LFNG O-fucosylpeptide 3-beta-N-acetylglucosaminyltransferase (Lfng)
Lgi4	-2.69	3.87	-0.28	leucine-rich repeat LGI family, member 4 (Lgi4)

Lipg	-0.87	4.96	-0.14	lipase, endothelial (Lipg)
Lpl	-3.05	-3.78	0.70	lipoprotein lipase (Lpl)
Ltc4s	-1.22	-2.73	0.50	leukotriene C4 synthase (Ltc4s)
Lurap1l	-1.86	-4.58	0.97	leucine rich adaptor protein 1-like (Lurap1l)
Lyrm7	-0.80	4.27	0.98	LYR motif containing 7
Lyz1	-2.38	-2.75	0.50	lysozyme 1 (Lyz1)
Lyz2	-2.83	-5.32	1.00	lysozyme 2
Lzts1	-1.88	-2.69	2.20	leucine zipper, putative tumor suppressor 1
Maf	-3.72	-3.30	1.15	avian musculoaponeurotic fibrosarcoma oncogene homolog (Maf)
Mboat7	-0.69	3.02	-0.35	membrane bound O-acyltransferase domain containing 7
Mcoln2	-2.28	2.95	0.76	mucolipin 2 (Mcoln2)
Mctp1	-2.22	2.69	0.77	multiple C2 domains, transmembrane 1 (Mctp1)
Mfap3l	-1.87	3.40	1.40	microfibrillar-associated protein 3-like (Mfap3l)
Mgat5	-1.29	2.65	1.43	mannoside acetylglucosaminyltransferase 5
Mib2	-2.68	3.22	-0.03	Mib2 protein
Ms4a4b	-2.09	-4.66	-0.40	membrane-spanning 4-domains, subfamily A, member 4B
Ms4a4d	-2.40	-4.65	-0.74	membrane-spanning 4-domains, subfamily A, member 4D (Ms4a4d)
Musk	-2.70	2.66	-0.19	muscle, skeletal, receptor tyrosine kinase (Musk)
Nav2	-0.61	2.74	-0.14	neuron navigator 2
Nckap1l	-0.89	8.15	-0.41	NCK associated protein 1 like (Nckap1l)
Nefl	-2.85	4.97	0.93	neurofilament, light polypeptide (Nefl)
Nefm	-2.38	3.21	-0.15	neurofilament, medium polypeptide (Nefm)
Nkd1	-1.12	-3.20	0.81	naked cuticle 1 homolog (Drosophila) (Nkd1)
Nmnat2	-1.01	2.62	-0.72	nicotinamide nucleotide adenylyltransferase 2 (Nmnat2)
Nov	-2.66	-4.56	1.69	nephroblastoma overexpressed gene (Nov)
Npy	-0.95	4.78	-0.14	neuropeptide Y (Npy)
Nt5e	-1.01	7.76	-0.80	5' nucleotidase, ecto (Nt5e)
Nxph3	-0.93	3.34	0.18	neurexophilin 3 (Nxph3)
Ogn	-2.16	-4.60	1.34	osteoglycin (Ogn)
Olfr1248	-0.70	3.94	-0.16	olfactory receptor 1248 (Olfr1248)
Olfr982	-2.82	-2.95	-2.83	olfactory receptor 982 (Olfr982)
Omd	-3.85	-5.55	1.19	osteomodulin (Omd)
Osbpl6	-1.03	3.86	2.69	oxysterol binding protein-like 6 (Osbpl6)
Pawr	-0.77	-3.77	1.35	PRKC, apoptosis, WT1, regulator (Pawr)

Pcdh10	-2.38	-3.11	2.84	protocadherin 10 (Pcdh10)
Pcdh7	-0.64	2.85	-0.26	protocadherin 7 (Pcdh7)
Pcsk6	-0.82	-3.59	1.90	proprotein convertase subtilisin/kexin type 6 (Pcsk6)
Pde6h	-0.69	3.48	-0.77	phosphodiesterase 6H, cGMP-specific, cone, gamma (Pde6h)
Pi15	-4.75	2.60	2.02	peptidase inhibitor 15 (Pi15)
Plet1	-1.27	6.43	-1.40	placenta expressed transcript 1 (Plet1)
Plp1	-1.77	5.15	0.49	proteolipid protein (myelin) 1 (Plp1)
Plpp3	-4.25	3.23	0.63	phospholipid phosphatase 3 (Plpp3)
Plxnc1	-1.02	-2.72	0.99	plexin C1 (Plxnc1)
Postn	-0.60	-3.82	-0.62	periostin, osteoblast specific factor (Postn)
Ppil3	-1.04	2.71	2.23	peptidylprolyl isomerase (cyclophilin)-like 3
Ppp1r3c	-6.03	-3.07	2.83	protein phosphatase 1, regulatory (inhibitor) subunit 3C (Ppp1r3c)
Prelp	-4.11	-2.88	1.60	proline arginine-rich end leucine-rich repeat (Prelp)
Prl2c1	-3.85	6.39	-0.15	Prolactin family 2, subfamily c, member 1 (Prl2c1)
Prl2c3	-4.27	7.71	-0.14	prolactin family 2, subfamily c, member 3 (Prl2c3)
Prl2c5	-4.29	7.23	-0.42	prolactin family 2, subfamily c, member 5 (Prl2c5)
Ptchd4	-1.84	-2.99	-0.36	patched domain containing 4
Ptk2b	-0.79	3.49	-0.39	PTK2 protein tyrosine kinase 2 beta (Ptk2b)
Ptn	-1.32	-9.48	-1.31	pleiotrophin (Ptn)
Ptpn22	-2.20	4.37	0.37	protein tyrosine phosphatase, non-receptor type 22 (lymphoid) (Ptpn22)
Ptx3	-3.10	-8.92	0.70	pentraxin related gene (Ptx3)
Rab19	-2.04	-3.61	2.41	RAB19, member RAS oncogene family (Rab19)
Rab30	-1.00	-4.24	1.32	RAB30, member RAS oncogene family (Rab30)
Rasgrf1	-1.16	5.69	-2.07	RAS protein-specific guanine nucleotide-releasing factor 1 (Rasgrf1)
Rasl11a	-1.55	-3.52	-0.01	RAS-like, family 11, member A (Rasl11a)
Rcsd1	-1.20	-3.48	1.71	RCSD domain containing 1 (Rcsd1)
Rcvrn	-0.85	3.41	3.09	recoverin (Rcvrn)
Rgs16	-2.17	3.72	-1.37	regulator of G-protein signaling 16 (Rgs16)
Rims4	-2.63	3.09	-0.15	regulating synaptic membrane exocytosis 4 (Rims4)
Rnase10	-1.09	-2.96	0.52	ribonuclease, RNase A family, 10 (non-active) (Rnase10)
Rnf144a	-1.97	-5.87	-0.47	ring finger protein 144A (Rnf144a)
Rspo2	-3.98	-4.39	0.66	R-spondin 2 (Rspo2)
Rundc3a	-0.96	4.05	-0.88	RUN domain containing 3A (Rundc3a)
Runx1t1	-1.22	-4.56	0.68	runt-related transcription factor 1; translocated to, 1 (cyclin D-related) (Runx1t1)

S100a8	-1.08	3.70	-0.13	S100 calcium binding protein A8 (calgranulin A) (S100a8)
S100b	-1.65	-3.40	-0.72	S100 protein, beta polypeptide, neural (S100b)
S1pr1	-3.84	3.41	0.19	sphingosine-1-phosphate receptor 1 (S1pr1)
Sapcd1	-1.36	6.88	0.71	suppressor APC domain containing 1 (Sapcd1)
Scara5	-5.54	-3.90	2.12	scavenger receptor class A, member 5 (Scara5)
Sema3c	-0.65	-6.36	1.14	sema domain, immunoglobulin domain (Ig), short basic domain, secreted, (semaphorin) 3C (Sema3c)
Sema4b	-0.81	3.17	0.45	sema domain, immunoglobulin domain (Ig), transmembrane domain (TM) and short cytoplasmic domain, (semaphorin) 4B
Sema6d	-1.45	3.44	-0.23	sema domain, transmembrane domain (TM), and cytoplasmic domain, (semaphorin) 6D (Sema6d)
Serpina3g	-3.79	-3.12	0.52	serine (or cysteine) peptidase inhibitor, clade A, member 3G (Serpina3g)
Serpina3k	-1.21	4.37	-0.14	serine (or cysteine) peptidase inhibitor, clade A, member 3K (Serpina3k)
Serpinb1a	-2.46	-3.89	-1.57	serine (or cysteine) peptidase inhibitor, clade B, member 1a (Serpinb1a)
Serpinb2	-1.42	4.96	-2.11	serine (or cysteine) peptidase inhibitor, clade B, member 2 (Serpinb2)
Serpinb3c	-1.42	3.75	1.46	serine (or cysteine) peptidase inhibitor, clade B, member 3C (Serpinb3c)
Serpine2	-3.64	3.89	0.10	serine (or cysteine) peptidase inhibitor, clade E, member 2 (Serpine2)
Shank1	-1.09	2.82	-0.23	SH3/ankyrin domain gene 1 (Shank1)
Slc11a1	-1.25	2.80	0.66	solute carrier family 11 (proton-coupled divalent metal ion transporters), member 1 (Slc11a1)
Slc14a1	-1.63	5.04	1.56	solute carrier family 14 (urea transporter), member 1 (Slc14a1)
Slc16a9	-1.30	-2.61	1.08	solute carrier family 16 (monocarboxylic acid transporters), member 9 (Slc16a9)
Slc9a9	-0.81	-2.98	-0.13	solute carrier family 9 (sodium/hydrogen exchanger), member 9 (Slc9a9)
Slpi	-5.26	5.93	-1.19	secretory leukocyte peptidase inhibitor (Slpi)
Smad9	-1.80	-3.22	1.58	SMAD family member 9 (Smad9)
Smpd3	-2.15	-5.60	1.15	sphingomyelin phosphodiesterase 3, neutral (Smpd3)
Sod3	-1.09	-6.79	-1.22	superoxide dismutase 3, extracellular (Sod3)
Sorbs2	-4.50	-3.11	2.95	sorbin and SH3 domain containing 2 (Sorbs2)
Sox9	-2.33	2.98	-0.92	SRY (sex determining region Y)-box 9 (Sox9)
Sparcl1	-0.75	5.45	-0.83	SPARC-like 1 (Sparcl1)
Spon2	-0.66	-3.70	-2.36	spondin 2, extracellular matrix protein (Spon2)
Srpx	-0.86	-3.22	0.35	sushi-repeat-containing protein (Srpx)
Ssbp2	-1.06	-3.16	0.05	single-stranded DNA binding protein 2 (Ssbp2)
Stam2	-0.90	2.59	1.41	signal transducing adaptor molecule (SH3 domain and ITAM motif) 2
Synpo2	-2.18	-4.00	2.87	synaptopodin 2 (Synpo2)
Syt1	-2.69	-2.77	2.13	synaptotagmin I (Syt1)

Tbc1d9	-2.84	3.18	1.26	TBC1 domain family, member 9 (Tbc1d9)
Tbxa2r	-1.14	-6.02	1.05	thromboxane A2 receptor (Tbxa2r)
Thbd	-2.91	3.09	1.51	thrombomodulin (Thbd)
Tm4sf1	-1.20	3.71	-1.41	transmembrane 4 superfamily member 1 (Tm4sf1)
Tmem176a	-2.77	-3.04	-1.03	transmembrane protein 176A (Tmem176a)
Tmem176b	-2.70	-2.86	-0.29	transmembrane protein 176B (Tmem176b)
Tmem179	-2.68	4.91	-1.11	transmembrane protein 179 (Tmem179)
Tmem45a	-0.93	-2.92	1.19	transmembrane protein 45a (Tmem45a)
Tmod1	-2.92	5.12	1.10	tropomodulin 1 (Tmod1)
Tnfrsf9	-1.47	3.17	1.96	tumor necrosis factor receptor superfamily, member 9 (Tnfrsf9)
Tnmd	-4.75	-2.80	1.40	tenomodulin (Tnmd)
Tns4	-5.03	3.08	-0.36	tensin 4 (Tns4)
Trdn	-1.04	-3.08	-2.95	triadin (Trdn)
Trib1	-1.88	2.71	-0.61	tribbles pseudokinase 1 (Trib1)
Trim29	-0.91	2.87	-1.23	tripartite motif-containing 29 (Trim29)
Tsga10	-1.77	2.86	0.87	testis specific 10 (Tsga10)
Tspan11	-3.75	5.67	-0.74	tetraspanin 11 (Tspan11)
Tspear	-3.36	3.34	-0.16	thrombospondin type laminin G domain and EAR repeats
Tusc5	-0.87	-3.90	1.14	tumor suppressor candidate 5 (Tusc5)
Txnip	-1.98	-4.15	-1.28	thioredoxin interacting protein (Txnip)
Ube2l6	-3.65	3.61	0.17	ubiquitin-conjugating enzyme E2L 6
Unc5a	-2.43	3.81	-3.41	unc-5 netrin receptor A (Unc5a)
Vil1	-2.94	-2.68	-2.58	villin 1 (Vil1)
Vwa7	-1.42	3.30	-0.14	von Willebrand factor A domain containing 7 (Vwa7)

Table S6. List of genes whose expression is regulated by FGF2 and further modulated by TGF-β2 in combination with FGF2

Gene Symbol	[control] vs [FGF2] log FC	[control] vs [TGF-β2] log FC	[FGF2] vs [TGF-β2 +FGF2] log FC	Description
Abca15	5.31	-0.05	-4.04	ATP-binding cassette, sub-family A (ABC1), member 15 (Abca15)
Ackr4	5.07	-0.04	-4.16	atypical chemokine receptor 4 (Ackr4)
Acta2	-4.99	1.60	3.36	actin, alpha 2, smooth muscle, aorta (Acta2)
Adamts8	8.04	-1.08	-3.79	disintegrin-like and metallopeptidase (reprolysin type) with thrombospondin type 1 motif, 8 (Adamts8)
Adcy1	-3.16	0.91	3.58	adenylate cyclase 1 (Adcy1)
Adcyap1	4.76	-0.04	-3.86	adenylate cyclase activating polypeptide 1 (Adcyap1)
Adrb3	-5.62	-4.44	-3.65	adrenergic receptor, beta 3 (Adrb3)
Agxt	-3.65	0.73	4.47	alanine-glyoxylate aminotransferase (Agxt)
Anxa8	-3.59	3.79	7.05	annexin A8 (Anxa8)
Apod	-3.55	-8.63	-5.13	apolipoprotein D (Apod)
Arfgef3	5.46	-0.03	-4.55	ARFGEF family member 3
Arhegf6	4.24	0.39	-3.33	Rac/Cdc42 guanine nucleotide exchange factor (GEF) 6
Btbd16	5.80	0.06	-5.05	BTB (POZ) domain containing 16 (Btbd16)
Car3	-7.54	-10.19	-5.29	carbonic anhydrase 3 (Car3)
Ccl11	3.87	-5.67	-7.44	chemokine (C-C motif) ligand 11 (Ccl11)
Ccl25	5.38	0.41	-5.34	chemokine (C-C motif) ligand 25
Cdc14a	7.02	1.23	-6.25	CDC14 cell division cycle 14A (Cdc14a)
Chl1	5.61	0.01	-4.70	cell adhesion molecule L1-like (Chl1)
Clec5a	4.28	-0.03	-3.37	C-type lectin domain family 5, member a (Clec5a)
Cyp1b1	-3.19	-0.45	-6.96	cytochrome P450, family 1, subfamily b, polypeptide 1 (Cyp1b1)
Dmp1	5.14	-0.03	4.30	dentin matrix protein 1 (Dmp1)
Dnmt3a	4.69	-0.03	-3.78	DNA methyltransferase 3A
Dock8	3.36	1.68	3.50	dedicator of cytokinesis 8 (Dock8)
Ednrb	5.75	-0.05	-4.84	endothelin receptor type B (Ednrb)

Eif4enif1	5.43	-0.04	-4.53	eukaryotic translation initiation factor 4E nuclear import factor 1
Ep300	7.15	-0.05	-6.25	E1A binding protein p300
Fgd3	-3.86	1.46	4.08	FYVE, RhoGEF and PH domain containing 3
Fgf10	3.87	0.00	-3.38	fibroblast growth factor 10
Hbegf	3.13	3.88	3.33	heparin-binding EGF-like growth factor (Hbegf)
Hcar2	-3.15	-3.52	4.40	hydroxycarboxylic acid receptor 2 (Hcar2)
Iigp1	-3.30	-5.82	-4.40	interferon inducible GTPase 1 (Iigp1)
Il22ra1	7.22	1.62	-6.32	interleukin 22 receptor, alpha 1 (Il22ra1)
Il33	7.34	-2.60	-4.33	interleukin 33 (Il33)
Itga8	5.45	-1.74	-6.18	integrin alpha 8 (Itga8)
Lce1f	4.93	5.36	3.44	late cornified envelope 1F (Lce1f)
Lpl	-3.20	-3.35	-4.49	lipoprotein lipase
Ly6c1	-4.02	-5.56	-3.77	lymphocyte antigen 6 complex, locus C1 (Ly6c1)
Mmp8	4.05	0.06	-5.05	matrix metallopeptidase 8 (Mmp8)
Myog	7.32	-0.04	-6.42	myogenin (Myog)
Nppc	4.99	-2.13	-4.89	natriuretic peptide type C (Nppc)
Ntn1	4.63	0.10	-3.76	netrin 1 (Ntn1)
Palmd	-3.13	2.72	3.62	palmdelphin (Palmd)
Ptn	-4.68	-1.42	-6.73	pleiotrophin (Ptn)
Retn	-6.26	-12.81	-4.91	resistin (Retn)
Rnf144a	-3.24	-1.91	-3.48	ring finger protein 144A (Rnf144a)
Sfrp1	-4.03	-5.55	-3.42	secreted frizzled-related protein 1 (Sfrp1)
Sgk1	6.93	-0.05	-6.03	serum/glucocorticoid regulated kinase 1
Slc26a2	3.93	0.43	-4.20	solute carrier family 26 (sulfate transporter), member 2 (Slc26a2)
Smoc1	-5.44	-3.34	3.54	SPARC related modular calcium binding 1 (Smoc1)
Stmn2	-3.66	2.07	4.97	stathmin-like 2 (Stmn2)
Tagln	-4.43	1.99	3.41	transgelin (Tagln)
Tbx2	4.10	-0.29	-4.39	T-box 2 (Tbx2)
Tnnt2	3.93	5.67	6.16	troponin T2, cardiac (Tnnt2)
Trim7	5.79	3.01	-3.97	tripartite motif-containing 7 (Trim7)
Trpm1	5.18	-0.04	-4.28	transient receptor potential cation channel, subfamily M, member 1 (Trpm1)
Tspan13	3.49	0.23	-4.54	tetraspanin 13 (Tspan13)

Ucp1	7.34	-0.05	-3.65	uncoupling protein 1 (mitochondrial, proton carrier) (Ucp1)
Zfp2	5.63	-0.04	-4.73	zinc finger protein 2 (Zfp2)
