# Supplementary material for: Fibroblast growth factor signals regulate transforming growth factor‐β‐induced endothelial‐to‐myofibroblast transition of tumor endothelial cells via Elk1
Source: Mol Oncol. 2019 Jun 19;13(8):1706–24. doi: 10.1002/1878-0261.12504 (PMC6670013; doi:10.1002/1878-0261.12504)
Supplement: Supplementary file 3 [file MOL2-13-1706-s003.pdf]

Supplemental Information

Fibroblast growth factor signals regulate transforming growth factor-β-induced endothelial-to-myofibroblast transition of tumor endothelial cells via Elk1

Yuichi Akatsu[1,2,‡], Naoya Takahashi[3,‡], Yasuhiro Yoshimatsu[3,‡], Shiori Kimuro[3], Tomoki Muramatsu[4], Akihiro Katsura[1], Nako Maishi[5], Hiroshi I. Suzuki[1,6], Johji Inazawa[4], Kyoko Hida[5], Kohei Miyazono[1], and Tetsuro Watabe[3,*]

[1]Department of Molecular Pathology, Graduate School of Medicine, The University of Tokyo, Tokyo, Japan,
[2]Biomedicine Group, Pharmaceutical Research Laboratories, Pharmaceutical Group, Nippon Kayaku Co., Ltd., Tokyo, Japan,
[3]Department of Biochemistry, Graduate School of Medical and Dental Sciences, Tokyo Medical and Dental University (TMDU), Tokyo, Japan,
[4]Department of Molecular Cytogenetics, Medical Research Institute, Tokyo Medical and Dental University (TMDU), Tokyo, Japan,
[5]Department of Vascular Biology and Molecular Pathology, Graduate School of Dental Medicine, Hokkaido University, Sapporo, Japan,
[6]David H. Koch Institute for Integrative Cancer Research, Massachusetts Institute of Technology, Cambridge, MA, USA.
[‡]These authors contributed equally to this work.

***Corresponding author**: Tetsuro Watabe; Department of Biochemistry, Graduate School of Medical and Dental Sciences, Tokyo Medical and Dental University (TMDU), Tokyo, Japan. E-mail: t-watabe@umin.ac.jp

Supplemental Figure legends

Fig. S1. Two modes of TGF-β-induced mesenchymal transition of tumor endothelial cells (TECs). Heterogeneous populations of TECs consisting of at least two different subgroups undergo TGF-β-induced mesenchymal transition (Endothelial-to-Mesenchymal Transition: EndMT). One subpopulation of TECs (pink) gives rise to α-SMA positive (+) myofibroblastic cells (red), while the other (yellow) produces α-SMA negative (-) nonmyofibroblastic mesenchymal cells (yellow) via endothelial-to-myofibroblast transition (End-MyoT) and endothelial-to-nonmyofibroblast transition (End-N-MyoT), respectively.

Fig. S2. Expression of various endothelial and mesenchymal markers in multiple types of endothelial and mesenchymal cells. Endothelial (MS-1 and TECs) and mesenchymal (MC3T3 and NIH3T3) cells were subjected to qRT-PCR analysis for the expression of Tie2 (A), VEGFR2 (B), Endoglin (C), α-SMA (D), SM22α (E), and VEGF-A (F). Error bars represent standard deviation. Student's *t*-test with two biological independent replicates were used to determine statistical significance; $*P < 0.05$; N.D., not detectable.

Fig. S3. Effects of TGF-β2, FGF2 and Infigratinib on the expression of endothelial and myofibroblast markers in TECs. TECs were cultured in the absence (-) or presence (+) of TGF-β2 in combination with FGF2 or Infigratinib (a pan-inhibitor of FGF receptors) for 72 h, followed by immunocytochemical analysis for α-SMA (green) and Tie2 (red) (Nuclei: Blue). Scale bar: 100 μm.

Fig. S4. Effects of TGF-β2 and FGF2 on the tube forming ability of TECs. (A) TECs were preincubated with or without TGF-β2, FGF2 or combination of both factors for 72 h, followed by embedding on Matrigel and incubation for 1 h, and photographed by phase-contrast microscopy (A). Tube length was quantified using ImageJ (B). Experiments were performed in duplicate. Scale bar: 200 μm. Error bars represent standard deviation. Student's *t*-test with two biological independent replicates were used to determine statistical significance; $*P < 0.05$; N.S., not significant.

Fig. S5. Effects of TGF-β2 and VEGF-A on the expression of myofibroblast markers in TECs. (A) TECs were cultured in the absence (-) or presence (+) of TGF-β2 for 72 h, followed by qRT-PCR analysis for the expression of VEGF-A. (B, C) TECs were cultured in the absence (-) or presence (+) of TGF-β2 in combination with VEGF-A for 72 h, followed by qRT-PCR analysis for the expression of α-SMA (B) and SM22α (C). Error bars represent standard deviation. Student's *t*-test with two biological independent replicates were used to determine statistical significance; $*P < 0.05$.

Fig. S6. Differential effects of FGF2 on the TGF-β2-mediated expression of various markers in TECs. Heatmap and hierarchical clustering of gene expression in TECs treated with FGF2, TGF-β2 or combination of both factors and analyzed using the Agilent Expression Array data (A). Results were normalized and log-transformed. Genes were clustered using the hierarchical method (B). This figure is supplementary to Fig. 5A and summarizes the results of genes whose expression is downregulated by TGF-β2.

Fig. S7. Differential effects of FGF2 and Infigratinib on the TGF-β2-mediated expression of various markers in TECs. Heatmap and hierarchical clustering of gene expression in TECs cultured in the absence (Control) or presence of TGF-β2 in combination with FGF2 or Infigratinib using the Agilent Expression Array data (A). Results were normalized and log-transformed. Genes were clustered using the hierarchical method (B). Transcripts upregulated by TGF-β2 were selected and further modulated by FGF2 in combination with TGF-β2. Expression profiles of almost all of the genes, i.e. Hbegf, Acta2, Rgs4, and Tagln are also shown in Fig. 5.

Fig. S8. Effects of TGF-β2 and FGF2 on the expression of myofibroblast markers and FGF2 in TECs. TECs were cultured with or without TGF-β2, FGF2 or combination of both factors for various time periods, followed by qRT-PCR analysis for the expression of α-SMA (A), Rgs4 (B) and FGF2 (C). Error bars represent standard deviation.

Fig. S9. Roles of TGF-β2 and FGF2 signals in the regulation of End-MyoT and End-N-MyoT of TECs. (A) Heterogeneous populations of TECs consisting of at least

two different subgroups undergo TGF-β-stimulated End-MyoT and End-N-MyoT. One subpopulation of TECs (pink) gives rise to α-SMA positive (+) myofibroblast-like cells (red), while the other (yellow) produces α-SMA negative (-) fibroblastic cells (yellow). TGF-β induces the formation of transcription factor complexes of MRTF-A and SRF, which results in the activation of α-SMA expression. The stromal components become contractile, and are potent to promote tumor formation. (B) FGF2 suppresses the effects of TGF-β to induce End-MyoT by inducing the formation of transcription factor complexes of Elk1 and SRF, which suppresses the α-SMA expression. The stromal components will contain active fibroblastic cells (blue), and become less potent to promote tumor formation.

Fig. S10. Differential effects of TGF-β2 on the FGF2-mediated expression of various markers in TECs. Heatmap and hierarchical clustering of gene expression in TECs treated with FGF2, TGF-β2 or combination of both factors and analyzed using the Agilent Expression Array data (A). Results were normalized and log-transformed. Genes were clustered using the hierarchical method (B). Expression profiles of some of the genes, i.e. Hbegf, Acta2, and Tagln are also shown in Fig. 5.

Fig. S11. Effects of TGF-β2, FGF2, and SB431542 on the expression of α-SMA and HB-EGF in TECs. TECs were cultured in the absence (-) or presence (+) of TGF-β2 in combination with FGF2 or SB431542 (an inhibitor of TGF-β type I receptor) for 72 h, followed by qRT-PCR analysis for the expression of SM22α (A) and HB-EGF (B). Error bars represent standard deviation. Student's *t*-test with two biological independent replicates were used to determine statistical significance; $*P < 0.05$; N.S., not significant.
